# Would restricting firearm purchases due to alcohol- and drug-related misdemeanor offenses reduce firearm homicide and suicide? An agent-based simulation

**DOI:** 10.1186/s40621-022-00381-x

**Published:** 2022-06-09

**Authors:** Magdalena Cerdá, Ava D. Hamilton, Melissa Tracy, Charles Branas, David Fink, Katherine M. Keyes

**Affiliations:** 1 Center for Opioid Epidemiology and Policy, Division of Epidemiology, Department of Population Health, New York University Grossman School of Medicine, New York, NY USA; 2 Department of Epidemiology, Mailman School of Public Health, Columbia University, New York, NY USA; 3 Department of Epidemiology and Biostatistics, School of Public Health, University at Albany, State University of New York, Rensselaer, NY USA; 4 Department of Population Health, Grossman School of Medicine, NYU Langone Health, 180 Madison Avenue, Room 416, New York, NY 10016 USA

**Keywords:** Firearm violence, Simulation, Substance use, Misdemeanor, Agent-based model

## Abstract

**Background:**

Substance-related interactions with the criminal justice system are a potential touchpoint to identify people at risk for firearm violence. We used an agent-based model to simulate the change in firearm violence after disqualifying people from owning a firearm given prior alcohol- and drug-related misdemeanors.

**Methods:**

We created a population of 800,000 agents reflecting a 15% sample of the adult New York City population.

**Results:**

Disqualification from purchasing firearms for 5 years after an alcohol-related misdemeanor conviction reduced population-level rates of firearm homicide by 1.0% [95% CI 0.4–1.6%] and suicide by 3.0% [95% CI 1.9–4.0%]. Disqualification based on a drug-related misdemeanor conviction reduced homicide by 1.6% [95% CI 1.1–2.2%] and suicide by 4.6% [95% CI 3.4–5.8%]. Reductions were generally 2 to 8 times larger for agents meeting the disqualification criteria.

**Conclusions:**

Denying firearm access based on a history of drug and alcohol misdemeanors may reduce firearm violence among the high-risk group. Enactment of substance use-related firearms denial criteria needs to be balanced against concerns about introducing new sources of disenfranchisement among already vulnerable populations.

**Supplementary Information:**

The online version contains supplementary material available at 10.1186/s40621-022-00381-x.

## Background

In 2019, 39,707 people in the USA died from firearm injuries: Six out of every 10 were suicide deaths, and 3 out of every 10 were homicide deaths. This translates to about 109 people dying from firearm injuries every day (Centers for Disease Control and Prevention (CDC) (2020)). Deaths due to firearms are only one outcome of firearm violence: It is estimated that about 120,000 people experience a non-fatal firearm injury per year (Gani et al. 2017; Gani 2017; Kaufman et al. 2021). The burden of morbidity and mortality related to firearms is a public health crisis in the USA and calls for effective, evidence-based legislation to stem the tide of firearm injury are growing (Barry et al. 2013).

Prohibiting access to firearms by groups of people at high risk of committing violence against themselves or others is one of the leading strategies to prevent firearm violence (Siegel et al. 2017). Federal statutes prohibit the purchase, ownership, and possession of firearms by persons convicted of a felony or a domestic violence misdemeanor, persons who were issued a domestic violence restraining order, those found to be an “unlawful user or addicted to any controlled substance,” those “committed to any mental institution,” among others (Wintemute 2015). Notably, federal law does not prohibit people who misuse alcohol from obtaining firearms (Branas et al. 2016; Carr et al. 2010). While limited data are available on the effectiveness of specific firearms denial criteria, and implementation of denial criteria varies across states, prior research suggests denying access to firearms to persons convicted of violent misdemeanors (Wintemute et al. 2001; Zeoli et al. 2017) and persons convicted or arrested for a felony (Wright et al. 1999) are associated with fewer firearm crimes and homicides. Our previous simulation study indicates that denying firearm purchases based on psychiatric disorders would reduce suicide among those denied firearms but would have varying impacts on population rates of firearm suicide depending on the prevalence of disqualification criteria (Keyes et al. 2019).

Heavy substance use is one of the strongest predictors of future firearm violence (Friedman 1998; Goldstein et al. 1989; Grann and Fazel 2004; McMillen et al. 1992; Swanson 1996; Dorn et al. 2012). A study examining substance misuse and violent crime found 20–25% of violence could be attributed to alcohol and drug use disorders (Grann and Fazel 2004). The relationship seems to be especially strong for alcohol and firearm violence: A systematic review and meta-analysis found that 34% of firearm homicide perpetrators, 30.2% of firearm homicide victims, and 25.7% of firearm suicide victims were acutely intoxicated at the time of the event (Branas et al. 2016).

While many states prohibit the purchase, ownership, and possession of firearms by people who exhibit problematic behaviors related to alcohol or drug use, the stated denial criteria are often vague and unenforceable, such as being treated for alcohol-related reasons and “alcoholism.” Substance-related interactions with the criminal justice system are a touchpoint when people with problematic substance use become “visible” and may offer an opportunity to identify and intervene on a group at high risk of firearm violence.

In the absence of actual cases of implementation of specific alcohol- and controlled substance-related denial criteria, we simulated the potential impact that a range of firearms denial criteria based on substance-related interactions with the criminal justice system could have on rates of firearm homicide and suicide. We used agent-based models and the best available data to simulate firearm purchase restrictions based on alcohol- and controlled substance-related misdemeanors in New York City (NYC). Simulated criteria included: (1) at least one alcohol-related misdemeanor conviction; (2) at least one alcohol-related arrest; (3) at least one drug misdemeanor conviction; and (4) at least one drug arrest. Examining this range of criteria allowed us to ask how expansive criteria would have to be to reduce population-level rates of firearm violence.

## Methods

We developed an agent-based model (ABM) simulating the dynamic processes contributing to firearm homicide and suicide among adults in NYC, including calibrating firearm ownership and carrying, drug and alcohol use and use disorders, substance and violent-related arrests and convictions, and the broader range of firearm-related outcomes such as assault. Additional file 1: Figure A1 in the Online Appendix illustrates the relations included in the model, which builds on our previous ABMs of violence in NYC (Cerdá et al. 2014, 2015, 2018a; Keyes et al. 2019). Data from NYC sources were used to parameterize and calibrate the model when possible; when NYC data were unavailable, national or other community-based data were used (see data sources in Additional file 1: Table A1). Key components of the model are summarized below. Additional details about model parameters and processes, including a description of the model following the ODD (Overview, Design concepts, Details) and ODD + D protocols (Additional file 1: Appendix 1) (Müller et al. 2013; Grimm et al. 2010), initialization parameters and default values (Additional file 1: Table A2), and flowcharts illustrating steps in the model (Figures A2–A3), as well as final calibration formulae for key model parameters, are included in the online Appendices.

**Table 1:** Annualized estimates of homicide and suicide, substance-related arrests and misdemeanors, and firearm carrying and ownership from the agent-based model and empirical data sources

	Model estimates (95% CI)^a^	Empirical estimates
Firearm-related homicide (rate/100,000)	4.11 (4.09, 4.14)	3.96^b^
Firearm-related suicide (rate/100,000)	1.01 (1.00, 1.02)	0.98^b^
Alcohol-related misdemeanor (rate/100,000)	115.1 (114.9, 115.2)	116.1^c^
Alcohol-related arrest (rate/100,000)	134.7 (134.6, 134.7)	134.9^c^
Drug-related misdemeanor (rate/100,000)	525.0 (524.6, 525.4)	525.7^c^
Drug-related arrest (rate/100,000)	1,329.0 (1,328.1, 1,329.9)	1.329.1^c^
Firearm ownership (%)	21.94 (21.91, 21.95)	22.03^d^
Firearm carrying status (%)	3.92 (3.91, 3.92)	3.94^d^

^a^Mean and 95% CI from 100 runs of ABM

^b^CDC WONDER. Underlying Cause of Death (2008–2014)

^c^New York State Division of Criminal Justice Services (DCJS, 2011–2014)

^d^National Comorbidity Survey Replication (NCS-R, 2001–2003)

**Table 2:** Simulation of gun-related homicide and suicide in New York City after implementation of substance-related ownership disqualifications, among the total agent population

Intervention	Gun-Related Homicide						Gun-Related Suicide					
	Rate/100,000 (95% CI)			% Decrease (95% CI)			Rate/100,000 (95% CI)			% Decrease (95% CI)		
	Mean	LL	UL	Mean	LL	UL	Mean	LL	UL	Mean	LL	UL
Baseline	4.11	4.09	4.14				1.01	1.00	1.02			
DUI misdemeanor conviction Last yr, 5 yr duration	4.07	4.05	4.10	0.95	0.35	1.56	0.98	0.97	0.99	2.96	1.89	4.03
DUI misdemeanor conviction Last yr, 10 yr duration	4.06	4.04	4.09	1.28	0.67	1.88	0.96	0.95	0.97	4.63	3.47	5.80
2 + DUI mis conviction Last 5 yrs, 10 yr duration	4.08	4.05	4.11	0.84	0.19	1.48	0.98	0.97	0.99	2.84	1.67	4.00
Any DUI last yr, 5 yr duration	4.07	4.04	4.10	1.08	0.43	1.74	0.97	0.96	0.98	3.68	2.39	4.96
Any DUI last yr, 10 yr duration	4.04	4.01	4.06	1.84	1.22	2.45	0.96	0.95	0.97	4.78	3.41	6.14
2 + DUI last 5 yrs, 10 yr duration	4.08	4.05	4.11	0.84	0.17	1.51	1.00	0.99	1.01	0.69	− 0.54	1.93
Drug misdemeanor conviction Last yr, 5 yr duration	4.05	4.02	4.07	1.63	1.08	2.17	0.96	0.95	0.97	4.58	3.36	5.81
Drug misdemeanor conviction Last yr, 10 yr duration	4.00	3.98	4.03	2.66	2.05	3.27	0.95	0.94	0.96	5.90	4.70	7.11
2 + Drug misdemeanor conviction Last 5 yrs, 10 yr duration	4.09	4.06	4.11	0.70	0.06	1.33	0.99	0.98	1.00	2.09	0.98	3.21
Any Drug arrest last yr, 5 yr duration	3.92	3.90	3.95	4.65	4.07	5.23	0.92	0.91	0.93	8.52	7.33	9.72
Any Drug arrest last yr, 10 yr duration	3.92	3.89	3.95	4.75	4.10	5.40	0.90	0.89	0.91	10.76	9.45	12.07
2 + Drug arrest last 5 yrs, 10 yr duration	4.09	4.06	4.12	0.62	− 0.06	1.30	0.98	0.96	0.99	3.23	2.03	4.43

### Agent population and neighborhoods

The baseline ABM used for initial parameterization has been described elsewhere (Cerdá et al. 2015, 2018a). Briefly, the population of 800,000 agents (maximum population due to computational limitation) was initialized to approximate a 15% sample of the NYC adult population aged 18–84 years in 2010. Agent attributes included age, sex, race/ethnicity, income, education, marital and relationship statuses, and agent behaviors including alcohol and drug use, alcohol and drug use disorder, a range of psychiatric disorders, gun carrying and purchasing, violence perpetration, and violence victimization. Agents were assigned to neighborhoods proportionate to size of the adult population, so that distributions of age, sex, race/ethnicity, and household income matched US Census data of the adult population for each of the 59 community districts within the five boroughs of NYC for the year 2010 (Census Bureau 2010). The year 2010 was chosen because the data used to parameterize agent behaviors were collected from the early-2000s through 2017. Individual behaviors were influenced by neighborhood characteristics and vice versa (see “Social networks and neighborhood influences”). When deriving probabilities from national data, we limited the data sample to those living in Metropolitan Statistical Areas to best represent the NYC population.

### Social network and neighborhood influences

Each agent was assigned a target number of close social ties, randomly selected from a uniform distribution ranging from 1–9, for an average of five social network members, equivalent to “close friends” based on the General Social Survey (Marsden 1987). Agents were matched based on age, sex, race/ethnicity, education, firearm ownership status, drinking status, drug use, and spatial proximity, such that agents who were more similar in terms of agent characteristics and geographically closer to each other were more likely to become social ties (Marsden 1987; McPherson et al. 2006). For simplicity, social network members matched to a particular agent at baseline remained part of that agent’s social network for the duration of the model run. Based on empirical social network literature (Tracy et al. 2016), having ties to other agents who were involved in gun violence informed prison sentence length, homicide, and suicide risk probabilities.

Agents were also embedded in neighborhoods with characteristics of their own, which influenced agent behavior and vice versa. These other neighborhood components (e.g., police officers, mental health treatment, incarceration) are described in Additional file 1: Online Appendix and our previous publications (Keyes et al. 2019; Cerdá et al. 2015, 2018b; Keyes et al. 2019) as they affected the dynamics of the model and resulting estimates.

### Process overview and scheduling

The model proceeded in discrete annual timesteps. Within each timestep, a series of modules processed in the following order: (1) aging, (2) death and rebirth, (3) recalculations of agent characteristic variables, including firearm ownership and substance use, (4) movement to a new location, (5) potential violent victimization and perpetration, homicide and suicide, (6) actual violent incidents, homicides, and suicides, and (7) arrests and sentencing (see Additional file 1: Figure A3 in Appendix 1 for a flow diagram depicting the processes at each step of the model). Within each module, agents and neighborhoods were processed in sequential order, except for the occurrence of actual violent incidents, for which potential perpetrators were randomly ordered when seeking out potential victims to ensure that all potential perpetrators were given an opportunity to commit violence throughout the model run. The pertinent information for these analyses is described below. A more in-depth description can be found in Additional file 1: Online Appendix.

#### (1) Aging and (2) mortality

At each timestep, agents aged by one year, and some agents died consistent with 2010 NYC adult all-cause mortality rates (New York City Department of Health and Mental Hygiene (2010)). Agents who died were replaced with adult agents.

#### (3) Agent characteristics

##### Gun carrying and ownership

Agents owned and/or carried firearms at each timestep. The model defined firearm ownership as the legal purchase of a gun and carrying represented access to a legally owned or illicit firearm that can be carried. In this model, carrying a firearm included illegal firearm ownership, thus firearm carrying did not solely depend on ownership but was positively associated with it. Once an agent was convicted of a felony, they could no longer own a firearm. If they owned a firearm at the time of conviction, it was removed. However, they could still carry a firearm.

Probabilities of firearm ownership and carrying were calibrated from the National Comorbidity Study Replication (NCS-R), a nationally representative survey of US adults carried out between 2001 and 2003 (Kessler and Merikangas 2004). Ownership and carrying were calibrated separately. Firearm carrying in the absence of ownership was used to represent the illicit firearms market as well as trading firearms outside of the legal market. Probabilities for each parameter were calculated based on sociodemographic characteristics, current drug use and alcohol use, alcohol and drug use disorders, history of violent victimization and perpetration, seven mental health disorders (antisocial behavior (ASB) disorder, generalized anxiety disorder (GAD), intermittent explosive disorder (IED), major depressive disorder (MDD), mania, posttraumatic stress disorder (PTSD), and psychosis), overnight hospitalization for a mental health disorder, and suicidal ideation and attempt. An agent's history of ownership and carrying also predicted current ownership, and current ownership also predicted carrying status.

The third option for gun possession was through an agent’s social network. An agent could use a firearm for a homicide or suicide if they had a friend who owned or carried a firearm.

##### Substance use and use disorder

Agents could be alcohol and drug users as well as diagnosed with a substance use disorder. Probabilities for alcohol and drug use and disorder were calculated from NCS-R (Kessler and Merikangas 2004).

Each agent was assigned a drinking status: non-drinker, light/moderate drinker, or heavy drinker. Men who had 5 + drinks a day or 21 + drinks a week and women who had 4 + drinks a day or 14 + drinks a week were considered heavy drinkers. At each timestep, the probability of being a light/moderate drinker and a heavy drinker was updated, and the agent could change their drinking status. Probabilities were calculated based on sociodemographic characteristics, current firearm ownership and carrying statuses, current drug use, and history of alcohol use, drug use, alcohol and drug use disorders, violent victimization and perpetration, and the seven mental health disorders listed above. Next, agents were given a probability of being a non-drinker by subtracting their probability of being a light/moderate and a heavy drinker from 1. Each agent was then assigned to be a non-drinker, light/moderate, or heavy drinker.

Agents were also assigned a probability of having an alcohol use disorder. Criteria for the probability calculations were the same as for drinking status. Only agents with a light/moderate or heavy drinking status could also have alcohol use disorder.

Agents were assigned a probability of using drugs based on NCS-R data (Kessler and Merikangas 2004), reflecting the use of cannabis, cocaine (any form), prescription drugs without being prescribed (i.e., tranquilizers, stimulants, pain killers, or other prescription drugs), or another drug (i.e., heroin, opium, glue, LSD, peyote, or any other drug) within the past 12 months. Probabilities were calculated based on the same criteria as drinking status. Similarly, agents were assigned a probability of having drug use disorder and were assigned to have a drug use disorder only if they also used drugs.

#### (4) Movement

A proportion of agents moved to a new neighborhood at each timestep in the model, and agents’ probabilities of moving were based on their income, duration of residence in their current neighborhood, and experiences of violent victimization at the last timestep, calibrated using data from longitudinal studies in urban areas (Goldmann et al. 2011) and the Panel Study of Income Dynamics (Sharkey 2013).

#### (5) Probabilities and 6) incidents of suicide, violence, and other health outcomes related to firearms

Suicide was influenced by history of suicide ideation and suicide attempt. Probabilities of suicide ideation and attempt were calculated from NCS-R (Kessler and Merikangas 2004). Probabilities of suicidal ideation and attempt were dependent on sociodemographic characteristics, current firearm ownership and carrying statuses, current alcohol and drug use (Blanco et al. 2013), history of: violent victimization and perpetration (Latalova et al. 2014), alcohol and drug use and? Disorders (Blanco et al. 2013), each of the seven mental disorders listed above, and mental health treatment (Blanco et al. 2013; Olfson et al. 2016). A suicide death was determined to be firearm-related if the agent owned, carried, or had access to a firearm through their social network.

At each timestep, agents were able to be violent victims or violent perpetrators. Probabilities of violent victimization and perpetration were calculated from NCS-R (Kessler and Merikangas 2004) dependent on sociodemographic characteristics, prior history of violent perpetration and victimization, history of mental disorder and mental health treatment, drug and alcohol use and use disorders, firearm ownership and carrying statuses, and neighborhood characteristics, and applied to the agents in the agent-based model. Violent perpetration was also based on history of any arrest based on NCS-R data (Kessler and Merikangas 2004). Similarly, probabilities were calculated from NCS-R for victimization and perpetration of intimate partner violence (IPV), however these probabilities were partially determined by history of IPV. The rates were calibrated to data from the National Intimate Partner and Sexual Violence Survey 2010 report (Black et al. 2011).

At each timestep, agents also died by homicide or suicide with or without a firearm based on Office of Chief Medical Examiner (OCME) data (Messner et al. 2007) and rates were calibrated to the average of 2008–2014 rates from CDC WONDER (Centers for Disease Control and Prevention, National Center for Health Statistics 1999). Probabilities were calculated based on race, sex, age, drug use, and heavy drinking status (Galea et al. 2008; Kaplan et al. 2012).

Potential victims and perpetrators (both IPV and non-IPV) were identified at each timestep, and violent incidents occurred, where a subset of these incidents were homicides. If the violent victim was also a potential homicide victim, based on OCME data (Messner et al. 2007), the violent incident would also become a homicide. If the victim or perpetrator owned, carried, or had access to a firearm through their social network, the homicide was determined to be firearm-related.

#### (7) Arrests

Four types of arrests and seven types of convictions were modeled. Agents were assigned probabilities at each timestep of having a violent arrest, an alcohol-related arrest, a drug-related arrest, or another type of arrest. Probabilities for each of these types of arrests were calculated from National Survey on Drug Use and Health (NSDUH) data (Substance Abuse and Mental Health Services Administration 2019), however, rates for these arrests were calibrated to New York State Division of Criminal Justice Services (DCJS) data on average arrest rates in 2011–2014 (New York State Division of Criminal Justice Services 2019). Probabilities were dependent on sociodemographic characteristics (i.e., age, sex, race/ethnicity, and household income), history of: suicidal ideation and attempt, MDD, GAD, mental health treatment, each of the arrest types, and current alcohol and drug use and disorder. Based on these probabilities, agents were set to have any of the four types of arrests.

Based on DCJS data (New York State Division of Criminal Justice Services 2019), an agent’s arrest had a probability of being a misdemeanor versus a felony. Once it was determined if an agent was arrested on a felony or misdemeanor charge, they were assigned a probability of conviction based on DCJS data, and specific to their race, sex, age, and borough. Agents were assigned a felony conviction, a misdemeanor conviction, or no conviction. Agents with a felony conviction were assigned a probability of incarceration based on NYC Department of Corrections (Department of Correction 2016) and Justice Atlas data (Cardora 2010). Details on incarceration are found in Additional file 1: Appendix 1.

### Model dynamics, emergent properties, and spatial characteristics

The model implemented several critical features of agent-based models, including collectives, emergence, sensing, decision making, interaction, adaptation, and stochasticity. *Collectives* were present in the model in the form of agents grouped in social networks, neighborhoods, and police patrol areas. Certain events, such as violent incidents or incarceration, *emerged* from the behaviors and interactions of agents, which in turn were influenced by the characteristics of their neighborhoods and the presence of police officers nearby. Agents *sensed* their own experiences, their friends’ experiences, and the characteristics of their neighborhood. Agent *decision making*, including enacting violence and moving, depended on their past experiences and own characteristics.

Violence only occurred in the model if two agents directly *interacted* in the physical space. Consistent with our prior work (Keyes et al. 2019), potential perpetrators (i.e., those with a high predicted probability of perpetrating violence) searched a 15-cell radius around their location for potential victims (i.e., those with a high probability of being victimized). Agents who had not already been victimized at that timestep were matched to a perpetrator unless a police officer was present within a 2-cell radius of the victim, in which case the potential victim was protected from violence.

*Adaptation* was modeled through an agent’s experience of violent or intimate partner perpetration and victimization. An agent involved in a violent incident had increased probabilities of being involved in future violent incidents, experiencing mental health issues, and inflicting self-harm in subsequent timesteps, which exemplifies the influence of violent involvement on re-victimization re-perpetration, and future psychological distress (Keyes et al. 2019; Norris 1992; Kennedy 1997).

Lastly, *stochasticity* was used in assigning agent characteristics and behaviors at model initialization and throughout the model runs. All agent demographic and behavioral parameters were interpreted as probabilities, then assigned by drawing a random number between 0 and 1 and comparing the selected number to the agent’s calculated probability. As a result, the population composition varied slightly across model runs, but population patterns of movement, drinking status, and violence matched expected estimates.

### Model calibration and intervention scenarios

The ABM estimates were derived using a well-established two-step process. First, during model calibration, ABM estimates were compared to empirical data on total and neighborhood-specific population composition. An iterative process (Jaffe et al. 2019) was then used to adjust predictive equations and initial conditions in the model until estimates closely matched the empirical data (see Table 1).

After a burn-in period to stabilize estimates, each model scenario was run 100 times for 30 years each and the mean across runs was reported for outcomes of interest; 95% confidence intervals (CI) reflect variation across the 100 runs. The model was developed using Recursive Porous Agent Simulation Toolkit for Java (RepastJ, version 3.0), and implemented in Eclipse (version 4.2).

Second, during the intervention scenarios, ownership prohibitions were implemented based on four criteria: (1) alcohol-related misdemeanor conviction, (2) alcohol-related arrest, (3) drug-related misdemeanor conviction, and (4) drug-related arrest. Each year, an agent could meet disqualification criteria, and a 5 or 10 year prohibition would begin. Agents meeting criteria for each prohibition were restricted from gun ownership, their firearm would be removed, and they could not legally purchase a gun for the prohibition period; gun carrying remained possible for prohibited agents and was calibrated in the model as described above. For each of the four criteria, three interventions were implemented: (a) Firearms were removed for five years after one event, (b) firearms were removed for ten years after one event, and (c) firearms were removed for ten years after two or more of the same event happened within a 5-year period.

### Sensitivity analysis

To examine the robustness of our results, we repeated the analyses in two ways, (1) decreasing the base rate of firearm ownership and (2) adjusting the social network influence on violence. First, the national estimate from NCS-R (21.9%) was likely an overestimate of firearm ownership in NYC. While the average ownership rate in New York State between 2007 and 2016 was 14% (Windrum et al. 2007), firearm ownership laws were stricter in NYC than the state overall. Therefore, we decreased the ABM rate of ownership in half from 22 to 11%, to test the sensitivity of results to variations in the rate of firearm ownership. Second, to test the robustness of our assumptions on the impact of social networks on agent behavior we varied the influence social networks had on them from 10 to 25%.

## Results

### Model calibration

Table 1 describes the distribution of demographics and key parameters of interest from the model at baseline and compares these distributions to empirical data sources. The simulated estimates were generally comparable to those observed in the empirical data.

### Effects of firearm disqualification on population rates of firearm homicide and suicide

We first tested whether substance-related firearm ownership disqualifications reduced firearm-related homicide and suicide rates in the general population (see Table 2). The base rate of firearm-related homicide was 4.11 per 100,000 agents [95% CI 4.09–4.14], and firearm-related suicide as 1.01 per 100,000 agents [95% CI 1.00–1.02]. Across the four disqualification criteria, a 10-year intervention was more effective at decreasing firearm-related homicide and suicide than a 5-year intervention after a single event.

When restricting firearm ownership based on an alcohol-related misdemeanor conviction, firearm-related homicide decreased by 1.0% [95% CI 0.4–1.6%] and 1.3% [95% CI 0.7–1.9%] for 5- and 10-year intervention scenarios respectively. Under this same restriction, firearm-related suicide decreased by 3.0% [95% CI 1.9–4.0%] and 4.6% [95% CI 3.5–5.8%] respectively. Disqualification based on any alcohol-related arrest produced a similar reduction in homicide than restrictions based on a misdemeanor conviction. Firearm-related homicide decreased by 1.1% [95% CI 0.4–1.7%] and 1.8% [95% CI 1.2–2.5%] for 5- and 10-year intervention scenarios respectively. Under this same restriction firearm-related suicide decreased by 3.7% [95% CI 2.4–5.0%] and 4.8% [95% CI 3.4–6.1%] respectively.

At the population level, drug-related firearm disqualification showed larger decreases in firearm-related homicide and suicide than alcohol-related restrictions. Firearm disqualification based on a drug-related misdemeanor conviction reduced firearm-related homicide by 1.6% [95% CI 1.1–2.2%] and 2.7% [95% CI 2.1–3.3%] for 5- and 10-year intervention scenarios respectively. Under this same restriction, firearm-related suicide decreased by 4.6% [95% CI 3.4–5.8%] and 5.9% [95% CI 4.7–7.1%] respectively. When the criteria expanded to any drug-related arrest, the decrease in homicide and suicide almost doubled. Firearm-related homicide decreased by 4.7% [95% CI 4.1–5.2%] and 4.8% [95% CI 4.1–5.4%] for 5- and 10-year intervention scenarios respectively. Under this same restriction, firearm-related suicide decreased by 8.5% [95% CI 7.3–9.7%] and 10.8% [95% CI 9.5–12.1%] respectively.

Implementing a 10-year firearms disqualification after the occurrence of two events in 5 years was less effective at reducing firearm-related homicide and suicide at the population level than restricting firearms after a single event. There was minimal evidence of a change in firearm-related homicide, as it decreased by less than 1% among the four event scenarios. Firearm-related suicide decreased by 2.8% [95% CI 1.7–4.0%] if firearms were restricted following two alcohol-related misdemeanor convictions in under 5 years, but suicide did not decrease if firearms were restricted after two alcohol-related arrests in 5 years. Firearm disqualification after two drug misdemeanor convictions in 5 years reduced firearm-related suicide by 2.1% [95% CI 1.0–3.2%], while disqualification after any two drug arrests decreased firearm-related suicide by 3.2% [95% CI 2.0–4.4%].

### Effects of firearm disqualification on rates of firearm homicide and suicide among high-risk groups

We examined the impact these interventions could have on homicide and suicide rates within the high-risk groups that were disqualified from purchasing firearms (Table 3). Base rates were determined by calculating firearm-related homicide and suicide rates among agents who had ever had a misdemeanor conviction or an arrest or had ever had two events in a 5-year period. Similar to the impact at the population level, disqualifications after a single event were more effective with a 10-year intervention than a 5-year intervention.

**Table 3:** Simulation of gun-related homicide and suicide in New York City after implementation of substance-related ownership disqualifications, among high-risk groups

Intervention	Gun-Related Homicide						Gun-Related Suicide					
	Rate/100,000 (95% CI)			% Decrease (95% CI)			Rate/100,000 (95% CI)			% Decrease (95% CI)		
	Mean	LL	UL	Mean	LL	UL	Mean	LL	UL	Mean	LL	UL
Alcohol Misdemeanor												
Base: Alcohol misdemeanor conviction ever	8.45	8.29	8.62				2.07	1.99	2.15			
Alcohol misdemeanor conviction ever, 5 yr duration	7.78	7.64	7.91	8.01	6.39	9.62	1.57	1.50	1.64	24.02	20.78	27.27
Alcohol misdemeanor conviction ever, 10 yr duration	7.72	7.60	7.84	8.70	7.25	10.15	1.52	1.45	1.58	26.63	23.35	29.92
Base: 2 + Alcohol misdemeanor conviction in 5 years, ever	8.22	7.25	9.20				5.31	4.73	5.89			
2 + Alcohol misdemeanor conviction in 5 years, ever	5.91	5.31	6.51	28.09	20.80	35.39	3.25	2.79	3.72	38.75	30.02	47.48
Alcohol Arrest												
Base: Any Alcohol ever	6.05	5.85	6.25				2.92	2.80	3.05			
Any Alcohol ever, 5 yr duration	4.63	4.50	4.77	23.43	21.22	25.63	1.90	1.79	2.01	35.01	31.38	38.64
Any Alcohol ever, 10 yr duration	4.39	4.23	4.56	27.40	24.69	30.11	1.90	1.79	2.00	35.18	31.73	38.63
Base: 2 + Alcohol in 5 years, ever	8.40	7.47	9.32				4.99	4.45	5.53			
2 + Alcohol in 5 years, ever	5.74	5.05	6.44	31.59	23.32	39.86	3.15	2.67	3.62	36.96	27.42	46.51
Drug Misdemeanor												
Base: Drug misdemeanor conviction ever	9.48	9.40	9.55				1.60	1.57	1.63			
Drug misdemeanor conviction ever, 5 yr duration	9.23	9.16	9.30	2.64	1.92	3.35	1.43	1.40	1.46	10.53	8.50	12.56
Drug misdemeanor conviction ever, 10 yr duration	9.16	9.09	9.23	3.31	2.56	4.05	1.39	1.36	1.42	13.32	11.34	15.30
Base: 2 + Drug misdemeanor conviction in 5 years, ever	12.36	11.89	12.83				3.11	2.87	3.34			
2 + Drug misdemeanor conviction in 5 years, ever	11.80	11.39	12.22	4.50	1.16	7.84	2.56	2.35	2.77	17.72	10.96	24.47
Drug Arrest												
Base: Any Drug arrest ever	8.31	8.22	8.40				1.86	1.82	1.89			
Any Drug arrest ever, 5 yr duration	7.40	7.33	7.48	10.93	10.04	11.82	1.42	1.38	1.45	23.73	21.73	25.73
Any Drug arrest ever, 10 yr duration	7.28	7.19	7.37	12.42	11.37	13.47	1.35	1.32	1.39	27.13	25.27	28.98
Base: 2 + Drug arrest in 5 years, ever	13.23	12.99	13.48				2.34	2.25	2.44			
2 + Drug arrest in 5 years, ever	12.54	12.31	12.77	5.24	3.52	6.97	2.06	1.96	2.15	12.14	8.17	16.12

Among agents who were ever convicted of an alcohol-related misdemeanor, firearm-related homicide decreased by 8.0% [95% CI 6.4–9.6%] and 8.7% [95% CI 7.3–10.2%] after 5- and 10-year interventions, respectively, and firearm-related suicide decreased by 24.0% [95% CI 20.8–27.3%] and 26.7% [95% CI 23.4–29.9%]. Among agents who ever had an alcohol-related arrest, firearm-related homicide decreased by 23.4% [95% CI 21.2–25.6%] and 27.4% [95% CI 24.7–30.1%] after 5- and 10-year interventions, respectively, and firearm-related suicide decreased by 35.0% [95% CI 31.4–38.6%] and 35.2% [95% CI 31.7–38.6%].

At the subpopulation level, drug-related restrictions were not as effective as alcohol-related interventions. Among agents who were ever convicted of a drug-related misdemeanor, firearm-related homicide decreased by 2.6% [95% CI 1.9–3.4%] and 3.3% [95% CI 2.6–4.1%] after 5- and 10-year interventions, respectively, and firearm-related suicide decreased by 10.5% [95% CI 8.5–12.6%] and 13.3% [95% CI 11.3–15.3%]. Among agents who ever had a drug-related arrest, firearm-related homicide decreased by 10.9% [95% CI 10.0–11.8%] and 12.4% [95% CI 11.4–13.5%] after 5- and 10-year interventions, respectively, and firearm-related suicide decreased by 23.7% [95% CI 21.7–25.7%] and 27.1% [95% CI 25.3–29.0%].

When examining alcohol-related subpopulations, 10-year firearm restrictions after two events in under 5 years were more effective than restrictions after a single event at reducing firearm-related homicide and suicide at the subpopulation level. Among agents who ever had two alcohol-related misdemeanor convictions in under 5 years, firearm-related homicide decreased by 28.1% [95% CI 20.8–35.4%] and firearm-related suicide decreased by 38.8% [95% CI 30.0–47.5%]. Among agents who ever had two alcohol-related arrests in under 5 years, firearm-related homicide decreased by 31.6% [95% CI 23.3–39.9%] and firearm-related suicide decreased by 37.0% [95% CI 27.4–46.5%].

Among agents who ever had two drug-related misdemeanor convictions in under 5 years, firearm-related homicide decreased by 4.5% [95% CI 1.2–7.8%] and firearm-related suicide decreased by 17.7% [95% CI 11.0–24.5%]. Among agents who ever had two drug-related arrests in under 5 years, firearm-related homicide decreased by 5.2% [95% CI 3.5–7.0%] and firearm-related suicide decreased by 12.1% [95% CI 8.2–16.1%].

### Sensitivity Analysis, reduced firearm ownership

Decreasing the ABM rate of firearm ownership in half, from 22 to 11%, reduced firearm ownership by 50%. As we found in our main results, drug-related interventions were more effective than alcohol-related interventions at reducing firearm-related homicide and suicide at the population level (Additional file 2: eTable 1). The effects at the subpopulation level among high-risk groups also replicated our main results, where firearm-related homicide and suicide had larger decreases in the alcohol-related subgroups than the drug-related subgroups (Additional file 2: eTable 2).

### Sensitivity analysis, varied social network influence

When we increased or decreased the impact of social network influence on violence from 15 to 25% or 10%, the results were similar to our main analyses. As can be seen in Additional file 2: eTables 3–6, drug-related interventions were more effective than alcohol-related interventions among the total population, and in subgroup analyses there were larger decreases in the alcohol-related subgroups than the drug-related subgroups.

## Discussion

Findings from our agent-based model suggest that denial of firearm purchases based on an alcohol- and drug-related misdemeanor conviction may produce a small reduction in population-level rates of firearm homicide and suicide, with the largest reduction resulting from firearm purchase denials due to drug-related misdemeanor convictions. At the same time, denial of firearm purchases may have a sizable impact on rates of firearm homicide and suicide among the subgroup of people meeting the denial criteria. Within these subpopulations, the greatest reduction in firearm homicide and suicide was found among people with a prior history of alcohol misdemeanors. Overall, denial of firearm purchases based on alcohol- and drug-related misdemeanor convictions produced a markedly larger reduction in rates of firearm suicide rates compared to firearm homicide. Expansion of denial criteria to people with a history of drug-related arrests could double the reduction in rates of firearm homicide and suicide among the general population, as well as among people with a history of drug misdemeanor arrests.

Firearms denial criteria based on a history of alcohol-related misdemeanor convictions are projected to produce small reductions in population-level rates of firearm homicide and suicide. However, disqualifications based on alcohol-related misdemeanor convictions have the potential to produce a larger decrease among those convicted, particularly in the risk of suicide. The substantially reduced risk of suicide within the high-risk group is supported by prior research. In particular, the firearm denial criteria raises barriers to the lethal means needed for a firearm suicide, likely overcoming the particularly strong relationship between heavy alcohol use and suicide (Branas et al. 2016). The minimal shift in population-level rates of firearm homicide following the simulated denial of the right to purchase firearms based on a prior history of alcohol-related misdemeanor convictions may be due to the small size of this high-risk group: In our population, only 116 per 100,000 had been convicted of an alcohol-related misdemeanor in the past year. The small size of this group is borne out in US data. In a study of legal handgun purchasers in California, for example, only 2% of 80,000 had a prior alcohol misdemeanor conviction (Schell et al. 2020; Kagawa et al. 2020).

As with alcohol-related misdemeanor convictions, the study suggested that disqualifying those with drug-related misdemeanor convictions was also associated with a slight reduction in population-level rates of suicide but sizable declines in suicide among those in the high-risk group. In contrast, expanding denials to those with a history of any drug-related arrests would reduce both population-level rates of firearm-related homicide and suicide and produce substantial reductions in firearm violence among those with a history of drug-related arrests. Drug use (Kaufman et al. 2020; Gilchrist et al. 2019; Atkinson et al. 2009), buying and selling drugs are associated with victimization and perpetration of interpersonal violence (Cafferky et al. 2018; Bellair and Mcnulty 2009), and existing literature also confirms a link between substance use disorders and suicide (Cerdá et al. 2010; Yen et al. 2003; Allebeck and Allgulander 1990). Our model simulated a simplified representation of the processes through which drug and alcohol use could increase the risk for violence by allowing the probability of violent perpetration and victimization to be partly predicted by an alcohol- or drug-related misdemeanor conviction or arrest. Further, alcohol and drug-related misdemeanor convictions and arrest, as well as firearm ownership, predicted which agents would be connected in a social network, and the prior history of violence among members in the network predicted the probability of victimization of each agent, thus simulating the confluence of substance-related interactions with law enforcement and firearm ownership in creating violent social contexts. At a broader level, however, our study findings suggest that regardless of whether there is a causal relationship between substance-related law enforcement interaction and firearm violence, a history of drug-related arrests may function as a useful touchpoint to identify individuals at high risk for firearm violence and that limiting the right to purchase firearms within this group may actually shift population-level rates of firearm violence.

### Applications of this study

Based on the existing evidence reviewed above, the Federal Consortium on Risk Based Firearms Policy recommends disqualifying those with a second drug- or alcohol-related misdemeanor offense from purchasing or possessing firearms. Our simulation study suggests that larger population-level shifts in firearm violence could be produced by disqualifying people from having firearms after the first drug- or alcohol-related conviction or arrest. However, such findings need to be considered alongside potential concerns with such high-risk approaches to addressing firearm violence, including whether targeting such groups may deter them from seeking treatment for substance use disorders, and whether this type of approach limits the rights of already disenfranchised groups. This is a particularly important concern given the documented racial/ethnic discrimination by police, and thus racial/ethnic inequalities in the probability of being arrested or convicted for a substance-related incident.

### Strengths and limitations

This study used a simulation approach, agent-based modeling, to predict the types of firearms denial criteria with the greatest potential to reduce rates of firearm violence. This approach efficiently combined a wide variety of data sources to provide quantitative estimates that can inform policy. ABMs were particularly suitable for the simulation of firearm violence, as the models incorporated spatial and situational dynamics that lead to the perpetration of violence, and considered how the intersection of law enforcement, social networks, and neighborhood context can shape firearm access and the spread of violent behaviors.

The study findings should be considered in light of several limitations. First, the validity of the simulation results depends on the assumptions made about the dynamics of violent behavior in our model, as well as the data we used to calibrate the model. We used NYC 2010 Census data, as well as data on NYC convictions, arrests, and mortality, among others, but also had to rely on national data to calibrate firearm ownership and carrying dynamics. The firearm homicide and suicide rates are much lower in NYC than in the USA as a whole (e.g., the rate of firearm-related mortality in 2011 4.3% in NYC vs 10% in the USA (Nilsson et al. 2014)). However, sensitivity analyses where we reduced firearm ownership by 50% showed our findings were robust to variations in this assumption. Future research will have to test whether findings generalize to different contexts, although our findings do indicate that among the small group of individuals that we could disqualify from purchasing firearms in our model, the reduction in firearm violence was very high. We would expect higher numbers of deaths prevented in places with a higher rate of firearm ownership. Second, this model assumes that all agents who met the disqualification criteria could be identified and disarmed. In reality, identification and enforcement of firearms denial criteria are often low, and legal loopholes reduce the efficacy of these measures. Hence, study findings are likely an upper bound of the potential impact of such firearms denial criteria in our study context. Third, we calibrated the illegal firearm marketplace by allowing agents to carry firearms they did not purchase; as data become available on the illegal marketplace dynamics, these simulations will become more robust.

## Conclusions

Firearm violence is one of the leading public health problems facing the USA today. Prohibiting access to firearms by high-risk groups is one of the main prevention strategies implemented to address this problem. While federal and state statute language is either absent or can be vague (with notable exceptions (Carr et al. 2010)) on the prohibition of firearm purchase and possession by individuals with a history of alcohol and controlled substance use, this study suggests that concrete indicators such as a prior history of convictions or arrest for drug- and alcohol-related crimes may be a promising strategy to produce small reductions in population rates of firearm violence. However, given concerns about stigma, disenfranchisement of historically marginalized groups, and racial/ethnic discrimination in policing, attempts to enact these types of denial criteria should be considered alongside population-level approaches that also address the root causes of firearm violence.

## Supplementary Information

**Additional file 1**. **Appendix 1** ODD+D Protocol. **Figure A1** Diagram of relations between agent, social network, and neighborhood characteristics in the agent-based model, Diagram of relationships in ABM. **Figure A2** Flow diagram illustrating processes occurring at each step of the model, Diagram of processes in model. **Figure A3**, Flow diagram illustrating steps in model initialization, Diagram of model initialization. **Table A1** Agent, social network, and neighborhood parameters, values, data sources, and update rules, Model parameters, values, data sources, and update rules. **Table A2** Agent-based model initialization parameters and default values, ABM initialization parameters

**Additional file 2**. **eTable 1** Sensitivity analysis reducing firearm ownership: simulation of gun-related deaths among the total agent population. **eTable 2** Sensitivity analysis reducing firearm ownership: simulation of gun-related deaths among the high-risk groups. **eTable 3** Sensitivity analysis decreasing social network influence: simulation of gun-related deaths among the total agent population. **eTable 4** Sensitivity analysis decreasing social network influence: simulation of gun-related deaths among the high-risk groups. **eTable 5** Sensitivity analysis increasing social network influence: simulation of gun-related deaths among the total agent population. **eTable 6** Sensitivity analysis increasing social network influence: simulation of gun-related deaths among the high-risk groups

## Data Availability

All data generated or analyzed during this study are included in Additional file 1: Appendix.

## Abbreviations

ABM: Agent-based model
ASB: Anti-social behavior disorder
CDC: Centers for Disease Control and Prevention
CI: Confidence intervals
DCJS: New York State Division of Criminal Justice Services
GAD: Generalized anxiety disorder
IED: Intermittent explosive disorder
IPV: Intimate partner violence
MDD: Major depressive disorder
NYC: New York City
NCS-R: National Comorbidity Study Replication
NSDUH: National Survey on Drug Use and Health
OCME: Office of Chief Medical Examiner
PTSD: Posttraumatic stress disorder
US: United States
