# Supplementary material for: Would restricting firearm purchases due to alcohol- and drug-related misdemeanor offenses reduce firearm homicide and suicide? An agent-based simulation
Source: Inj Epidemiol. 2022 Jun 9;9:17. doi: 10.1186/s40621-022-00381-x (PMC9185952; doi:10.1186/s40621-022-00381-x)
Supplement: Supplementary file 1 — Additional file 1. Appendix 1 ODD+D Protocol. Figure A1 Diagram of relations between agent, social network, and neighborhood characteristics in the agent-based model, Diagram of relationships in ABM. Figure A2 Flow diagram illustrating processes occurring at each step of the model, Diagram of processes in model. Figure A3, Flow diagram illustrating steps in model initialization, Diagram of model initialization. Table A1 Agent, social network, and neighborhood parameters, values, data sources, and update rules, Model parameters, values, data sources, and update rules. Table A2 Agent-based model initialization parameters and default values, ABM initialization parameters [file 40621_2022_381_MOESM1_ESM.docx]

**Appendix 1. Description of ABM using the ODD protocol**

A detailed description of the ABM is provided below, following the ODD (Overview, Design concepts, Details) and ODD + D protocols[1–3].

*Purpose*

The objectives of this ABM were to simulate hypothetical policy enactments of firearm disqualification criteria related to drug and alcohol abuse, and compare the impacts of the various interventions aimed at reducing gun-related homicide and suicide. The firearm disqualification interventions chosen were for four groups, a) alcohol-related misdemeanor conviction, b) any alcohol-related arrest, c) drug-related misdemeanor conviction, and d) any drug-related arrest.

*Entities, state variables, and scales*

The model consists of four types of entities: *agents*, *neighborhoods*, *police officers,* and *police patrol areas*. Individual *agents* are characterized by the static and time-varying variables listed in Table A1, including age, sex, race/ethnicity, educational attainment, household income, duration of residence, drinking status, firearm ownership and carrying statuses, and mental health diagnoses as well as variables indicating their location in the physical space. Individual behaviors include aging, dying, moving to a new neighborhood, going to prison, violent perpetration and victimization, and firearm and non-firearm related homicide and suicide. Agents are connected to each other in a social network, created by linking agents with similar characteristics who are also in close spatial proximity to each other. The relationships between these entities are seen in Figure A1.

The model physical environment consists of a rectangular 400 × 625 grid of cells divided into 59 *neighborhoods* representing the New York City (NYC) community districts[4]. Each neighborhood is characterized by its x- and y- boundaries, location on the grid, and list of resident agents.

*Police officers* are characterized by their location on the grid and the distance over which they can prevent violence; their only behavior is preventing the occurrence of violence when a potential perpetrator encounters a potential victim. *Police patrol areas* are 5 × 5 cell squares characterized by their x- and y- boundaries, coordinates of their center cell, and presence of a police officer.

Each time step of the model represents one year. Simulations were run for 140 years, with the first 110 years discarded. The first ten are a “burn-in period,” during which the agent population does not age or die, including from homicide or suicide. The next 100 years are also discarded to allow all agents to cycle through a lifespan, as well as accumulate a history of violence, and other agent characteristics. After the burn-in period, interventions are implemented on a settled population.

*Process overview and scheduling*

The model proceeded in discrete annual time steps. Within each time step, eight modules were processed in the following order: (1) aging, (2) death and rebirth, (3) recalculations of agent characteristic variables, including mental health diagnoses and treatments, (4) movement to a new location and reset locations of police officers, (5) potential violent victimization and perpetration, homicide and suicide, (6) actual violent incidents, homicides, and suicides, (7) arrests and sentencing, and (8) updates to neighborhood characteristics (see Figure A2 for a flow diagram depicting the processes at each step of the model, and below for pseudo-code for the model). Within each module, agents and neighborhoods were processed in sequential order, except for the occurrence of actual violent incidents, for which potential perpetrators were randomly ordered when seeking out potential victims to ensure that all potential perpetrators were given an opportunity to commit violence throughout the model run.

*Design concepts*

The model implemented several key features of agent-based models, including emergence, learning, sensing, interaction, stochasticity, and collectives. Specifically, *emergence* was present in that population levels of violence, depression, drug use, suicidal ideation and attempt, and incarceration emerged from the behaviors and interactions of agents, which in turn were influenced by the characteristics of their neighborhoods and the presence of police officers nearby. *Adaptation* was modeled in that one’s own mental health, arrest and prison record, drinking behaviors and violent experiences, as well as exposure to drinking behaviors and violence in one’s social network, modified an agent’s probability of these behaviors at future time steps; decisions about moving were also based on experiences of violence.

Regarding *sensing*, individual agents were assumed to know their own characteristics (e.g., age, sex) as well as their firearm ownership status, drinking status, and violent experiences of their friends, which influenced their own behaviors. They were also assumed to know the characteristics of the neighborhoods in the model, which could influence their behaviors and guide their selection of a new neighborhood when moving. Agents with the potential to perpetrate violence could also detect if potential victims were nearby, whereas police officers could sense when potential victims and perpetrators were in close proximity to each other and thus in danger of actual violence.

*Interaction* was critical to the model dynamics and outcomes, in that violence occurred through the direct interaction of a potential victim and potential perpetrator in the physical space. Interactions between police officers and potential victims and perpetrators were also capable of preventing violence from occurring. Furthermore, agents were assumed to interact with other agents in their social network with the experiences of violent victimization and incarceration of these other agents capable of influencing the agent’s own risks for homicide and incarceration.

*Stochasticity* was used in assigning agent characteristics and behaviors, at model initialization and throughout the model runs. Specifically, all agent demographic and behavioral parameters were interpreted as probabilities, with characteristics and behaviors assigned by drawing a random number between 0 and 1 and comparing the selected number to the agent’s calculated probability; this allowed the model population’s characteristics and behaviors to match expected distributions. Parameters that were not probabilities (e.g., influence of social network on homicide) were drawn from normal distributions so that average values for the population matched expected estimates but some variability existed in the population. As a result, the population composition varied slightly across model runs but population patterns of movement, drinking status, and violence demonstrated expected frequencies and correlates.

*Collectives* were present in the model in the form of agents grouped together in social networks, neighborhoods, and police patrol areas. Characteristics of all agents located within the boundaries of each neighborhood or patrol area were averaged to derive the average levels of income, violence, depression, drug use, suicide ideation and attempt, and firearm ownership in each area.

Finally, to allow *observation* for model testing, the values of agent and neighborhood parameters were recorded for each unit at each time step. For model analysis, only population-level variables were recorded at each time step (e.g., percent of agents who were victimized). To account for the stochastic nature of the model, each model scenario was run 20 times, with the mean, 2.5^th^ percentile, and 97.5^th^ percentile reported from across the 20 runs.

*Initialization*

At model initialization, the agent population consisted of 800,000 individuals aged 18-84 years with socio-demographic characteristics assigned to match distributions of the adult population of NYC according to the 2010 U.S. Census[5] (see Tables A1 and A2 and Figure A3).

The grid representing the physical space was divided into 59 areas reflecting the NYC community districts, with sizes proportional to community district land areas and locations consistent with adjoining community district borders[4]. Agents were assigned to each neighborhood at initialization on the basis of age, sex, race/ethnicity, and household income so that the composition of the area matched the American Community Survey data for the respective community district, including proportionate population size [6,7]. Neighborhood residence was then used to assign additional socio-demographic characteristics based on American Community Survey data surrounding 2010 [6,7], including unemployment, employment in managerial or professional occupations, foreign-born status, and status as a female-headed household with children under the age of 18 years. These characteristics were used to specify neighborhood-level influences on risk of violence, homicide, and suicide, and remained unchanged throughout the model run.

Agents were grouped into social networks as follows. First, each agent was assigned a target number of social network members, randomly selected from a uniform distribution ranging from 1-9, for an average of five social network members, equivalent to “close friends” based on the General Social Survey (GSS), a national survey of non-institutionalized U.S. adults repeated every two years[8]. Second, agents were matched with other agents in the population, based on spatial proximity (within 100 cells on the grid), age within 10 years, same sex, same race/ethnicity, same education level, same drinking status, and same firearm ownership status, with probabilities of matches for all but firearm-ownership status adjusted so that variability in the composition of social network members reproduced observed patterns in GSS data[8]. Agents had up to 10,000 attempts to find suitable matches. This iterative process was repeated until as many agents as possible were matched to their target number of social network members.

The total number of police officers in the model was based on a 5% sample of the average police force in New York City from 1990-1993, the years before the police force was increased as part of the order-maintenance policing strategy championed by Police Commissioner William Bratton and Mayor Rudolph Guiliani in 1994. At initialization, the number of police officers assigned to each neighborhood was proportional to the neighborhood population size; within the neighborhood, police officers were assigned to random locations.

Other parameters set during the initialization of the model are listed in Table A2.

Given previous evidence for the influence of neighborhood characteristics on drinking and exposure to violence[9–12], we allowed ten percent of individual agents’ probabilities of homicide, suicide, and violent victimization and perpetration to be determined by their neighborhood characteristics. Furthermore, given documented evidence for the influence of social networks on violence exposure and violent behaviors[13], we allowed fifteen percent of agents’ probabilities of homicide, suicide, and violent victimization and perpetration to be determined by the characteristics of the agents in their social network. The radius within which perpetrators searched for victims was set at initialization to 15 cells and the radius within which police officers could prevent violence was set at 2 cells.

*Input data*

The environment is assumed to be constant, so no input data were needed to represent time-varying processes.

*Submodels*

Each of the nine modules implemented every time step are described in greater detail below to include specific equations and data sources used to calculate behavioral probabilities.

1. Aging: Following the burn-in period, each agent aged by one year at each time step.
2. Death and rebirth: After the initial “burn-in period” of ten years, some agents died at each

time step. Probabilities of all-cause mortality were assigned to agents based on their age, sex, and race/ethnicity so that mortality rates in the agent population matched those in the NYC adult population in the year 2010[14]. Each agent who died was replaced with an 18-year-old agent with the same sex, race/ethnicity and neighborhood location as the deceased agent. Probabilities were re-run for marital status and income as they may have changed during the agent’s previous life. This procedure maintained a constant population size and composition in the model, except for age structure.

1. Recalculations of agent characteristic variables: Each agent recalculates many

characteristics at each time step. The probabilities are estimated using their statuses from the previous time step of other attributes. The values of the parameters are chosen so that the rates match rates estimated in survey data and other published estimates.

Probabilities were calculated for substance use and abuse. First, individual-level probabilities were calculated for drinking status (light drinker and heavy drinker) and drug use from a multinomial logistic regression model based on data from National Comorbidity Survey Replication (NCS-R):

1. logit(P_LIGHTDRK1) = -1.8407+ (0.7772*MALE)+ (-0.288*AGE1)+ (-0.1755*AGE3)+ (-0.277*AGE4)+ (-0.746*AGE5)+ (-1.109*AGE6)+ (0.3105*WHITE)+ (-0.4232*HISP)+ (-0.3307*OTHER_RACE)+ (-0.3794*HS)+ (0.0904*MOREHS)+ (0.2474*INC2)+ (0.4052*INC3)+ (0.4971*INC4)+ (-0.3568*MARRIED)+ (-0.3779*DIVSEPWID)+ (0.0152*HISTVIOLPERP)+ (0.0106*HISTVIOLVICT)+ (0.1172*LASTOWN)+ (0.2845*LASTCARRY)+ (0.0488*HISTALCAB)+ (1.2287*HISTMDD)+ (-0.3352*HISTGAD)+ (0.5909*HISTMANIA)+ (-0.1307*HISTASB)+ (-0.4382*HISTPSYCHO)+ (-0.0736*HISTPTSD)+ (-0.0648*LASTIPVVICT)+ (0.1645*LASTIPVPERP)+ (0.0154*HISTONHOSP)+ (0.3208*HISTIED)+ (-0.1986*HISTDRUGAB)+ (0.1447*LASTCURDRUGUSER)+ (0.4529*HISTLIGHTDRK)+ (0.1889*HISTHEAVYDRK)
2. logit(P_HEAVYDRK1) = -3.500+ (1.018*MALE)+ (1.8526*AGE1)+ (0.7681*AGE2)+ (-0.926*AGE4)+ (-0.7610*AGE5)+ (-7.6692*AGE6)+ (-0.0722*BLACK)+ (0.4770*HISP)+ (-0.2684*OTHER_RACE)+ (0.1072*HS)+ (0.2048*MOREHS)+ (-0.0912*INC2)+ (-0.1605*INC3)+ (-0.002*INC4)+ (0.0212*MARRIED)+ (0.3338*DIVSEPWID)+ (0.1727*HISTVIOLPERP)+ (0.2063*HISTVIOLVICT)+ (0.1402*LASTOWN)+ (0.1487*LASTCARRY)+ (1.0411*LASTCURDRUGUSER)+ (-0.4059*HISTDRUGAB)+ (1.4851*HISTALCAB)+ (0.0542*HISTMDD)+ (-0.0378*HISTGAD)+ (0.1056*HISTMANIA)+ (-0.2774*HISTASB)+ (0.2242*HISTIED)+ (-0.8866*HISTPSYCHO)+ (-0.1627*HISTPTSD)+ (-0.2472*LASTIPVVICT)+ (0.6996*LASTIPVPERP)+ (0.3528*HISTONHOSP)+ (-0.9777*HISTLIGHTDRK)+ (0.7118*HISTHEAVYDRK)
3. P_LIGHTDRK1 = exp(logit(P_LIGHTDRK1))/(1 + exp(logit(P_LIGHTDRK1))

+ exp(logit(P_HEAVYDRK1)))

1. P_HEAVYDRK1 = exp(logit(P_HEAVYDRK1))/(1 + exp(logit(P_LIGHTDRK1)) + exp(logit(P_HEAVYDRK1)))
2. P_NONDRK1 = 1 – P_LIGHTDRK1 – P_HEAVYDRK1
3. logit(P_DRUGUSER1) = -4.1189 + (0.4603*MALE)+ (1.905*AGE1)+ (0.8011*AGE2)+ (-0.7042*AGE4)+ (-1.3790*AGE5)+ (-4.937*AGE6)+ (-0.4698*WHITE)+ (-0.404*HISP)+ (0.2406*OTHER_RACE)+ (0.217*HS)+ (0.2314*MOREHS)+ (0.0243*INC2)+ (0.0269*INC3)+ (-0.0885*INC4)+ (-0.458*MARRIED)+ (0.1922*DIVSEPWID)+ (0.615*HISTVIOLPERP)+ (0.4852*HISTVIOLVICT)+ (1.9331*LASTCURLIGHTDRK)+ (2.1411*LASTCURHEAVYDRK)+ (-0.091*LASTOWN)+ (-0.5414*LASTCARRY)+ (0.2825*HISTALCAB)+ (1.1619*HISTDRUGAB)+ (0.1321*HISTMDD)+ (0.2387*HISTGAD)+ (-0.0435*HISTMANIA)+ (-0.3017*HISTASB)+ (0.1242*HISTIED)+ (0.3528*HISTPSYCHO)+ (0.0107*HISTPTSD)+ (0.1175*LASTIPVVICT)+ (0.1966*LASTIPVPERP)+ (0.1914*HISTONHOSP)
4. P_DRUGUSER1 = exp(logit(P_ DRUGUSER1))/(1 + exp(logit(P_DRUGUSER1)))

where

P_NONDRK1, P_LIGHTDRK1, P_HEAVYDRK1 = probability of being a given drinking

status at current time step

P_DRUGUSER1 = probability of being a drug user at current time step

MALE = dummy variable indicating male gender (female is referent)

WHITE, BLACK, HISP, OTHER_RACE = dummy variables indicating race (reference is

different in each equation)

AGE1-AGE6 = dummy variables indicating age 18-24(AGE1), 25-34 (AGE2), 35-44 (AGE3),

45-54 (AGE4), 55-64 (AGE5), and ≥ 65 (AGE6) years (reference is different in each equation)

MARRIED, DIVSEPWID = dummy variables indicating marital status, married (MARRIED) or

divorced/separated/widowed (DIVSEPWID) (never married is referent)

HS, MOREHS = dummy variables indicating high school degree or equivalent (HS) or

more than high school education (MOREHS) (less than high school is referent)

INC2-INC4 = dummy variables indicating household income of $20,000-$39,999 (INC2),

$40,000-$74,999 (INC3), and ≥ $75,000 (INC4) (< $20,000 is referent)

HISTVIOLPERP = dummy variable indicating if agent was a perpetrator at a previous time step

HISTVIOLVICT = dummy variable indicating if agent was victimized at a previous time step

LASTOWN = dummy variable indicating if agent owned a firearm at the last time step

LASTCARRY = dummy variable indicating if agent carried a firearm at the last time step

HISTMDD = dummy variable indicating if agent had a diagnosis for depression (MDD) at a

previous time step

HISTGAD = dummy variable indicating if agent had a diagnosis for anxiety (GAD) at a previous

time step

HISTMANIA = dummy variable indicating if agent had a diagnosis for mania at a previous time

step

HISTASB = dummy variable indicating if agent had a diagnosis for antisocial behavior (ASB) at

a previous time step

HISTIED = dummy variable indicating if agent had a diagnosis for intermittent explosive

disorder (IED) at a previous time step

HISTPSYCHO = dummy variable indicating if agent had a diagnosis for psychosis (PSYCHO)

at a previous time step

HISTPTSD = dummy variable indicating if agent had a diagnosis for post-traumatic stress

disorder (PTSD) at a previous time step

HISTALCAB = dummy variable indicating if agent had a diagnosis for alcohol abuse (ALCAB)

at a previous time step

HISTDRUGAB = dummy variable indicating if agent had a diagnosis for drug abuse

(DRUGAB) at a previous time step

HISTONHOSP = dummy variable indicating if agent had an overnight hospitalization to treat a

mental health disorder (ONHOSP) at a previous time step

LASTIPVVICT = dummy variable indicating if agent was a victim of intimate partner violence

(IPV) at the last time step

LASTIPVPERP = dummy variable indicating if agent was a perpetrator of intimate partner

violence (IPV) at the last time step

LASTDRUGUSER = dummy variable indicating if agent was a drug user at last time step

HISTLIGHTDRK = dummy variable indicating if agent has been a light drinker at a previous

time step

HISTHEAVYDRK = dummy variable indicating if agent has been a heavy drinker at a previous

time step

LASTLIGHTDRK = dummy variable indicating if agent was a light drinker at last time step

LASTHEAVYDRK = dummy variable indicating if agent was a heavy drinker at last time step

Next, neighborhood-level conditions influenced the probability of drinking status and drug use based on the World Trade Center study (WTC) data according to the following equations:

1. [logit(P_LIGHTDRK2) = 2.7799+ (-0.2058*HOODINC1)+ (0.9102*

HOODINC2)+ (-1.1312*PBLACK)+ (0.7093*PHISP)+ (1.0049* PHOODVIOL)+ (1.1555*PHOODMDD)+ (-0.1692*PHOODDRGUSE())+ (5.6767*PLIGHTDRK)+ (3.0991*PHEAVYDRK)+ (0.0341*PHOODHOM)+ (-0.0215*PHOODSUIC)+ (0.0171* PHOODOWN)+ (-15.8291* PHOODSUICATT)+ (6.037*PHOODSUICTHT)+ (-0.0885* HOODINC2* PHOODHOM)+ (-10.8297*PBLACK*PHOODVIOL)+ (-0.2093*PHISP* PHOODHOM)+ (0.406*PBLACK*PHOODSUIC)+ (-6.1838*HOODINC2* PHOODMDD)

1. logit(P_HEAVYDRK2) = -11.998+ (-82.2406*HOODINC1)+ (-31.0135*

HOODINC2)+ (144.5664*PBLACK)+ (11.6796*PHISP)+ (38.1817* PHOODVIOL)+ (-154.789*PHOODMDD)+ (-76.223*PHOODDRGUSE())+ (27.1769*PLIGHTDRK)+ (225.7925*PHEAVYDRK)+ (-1.0734* PHOODHOM)+ (-0.5681*PHOODSUIC)+ (0.2623*PHOODOWN)+ (148.1979* PHOODSUICATT)+ (274.575*PHOODSUICTHT)+ (410.1704*HOODINC1* PHOODVIOL)+ (99.7974*HOODINC2*PHOODVIOL)+ (-342.895*PBLACK *PHOODVIOL)+ (-5.5424*HOODINC1*PHOODHOM)+ (-1.8592* HOODINC2*PHOODHOM)+ (5.5545*PBLACK*PHOODHOM)+ (13.5014* PHISP*PHOODHOM)+ (1.8794*HOODINC1*PHOODSUIC)+ (-2.012* HOODINC2*PHOODSUIC)+ (-9.5888*PBLACK*PHOODSUIC)+ (607.6363* HOODINC1*PHEAVYDRK)+ (476.2707*HOODINC2*PHEAVYDRK)+ (-800.366*PBLACK*PHEAVYDRK)+ (-1328.5*PHISP*PHEAVYDRK)+ (8.5202*HOODINC1*PHOODOWN)+ (2.8394*HOODINC2*PHOODOWN)+ (-9.1667*PBLACK*PHOODOWN)+ (-9.966*PHISP*PHOODOWN)+ (195.5097*HOODINC1*PHOODMDD)+ (255.5237*HOODINC2* PHOODMDD)+ (-371.917*PBLACK*PHOODMDD)+ (-381.948*HOODINC1* PHOODDRGUSE)+ (-317.23*HOODINC2*PHOODDRGUSE)+ (294.5218* PBLACK*PHOODDRGUSE)+ (759.4591*PHISP*PHOODDRGUSE)

1. P_LIGHTDRK2 = exp(logit(P_LIGHTDRK2))/(1 + exp(logit(P_LIGHTDRK2))

+ exp(logit(P_HEAVYDRK2)))

1. P_HEAVYDRK2 = exp(logit(P_LIGHTDRK2))/(1 + exp(logit(P_LIGHTDRK2))

+ exp(logit(P_HEAVYDRK2)))

1. P_NONDRK2 = 1 – PLIGHTDRK2 – PHEAVYDRK2
2. logit(P_DRUGUSER2) = -4.8821+ (-0.0151*HOODINC1)+ (0.3313* HOODINC2)+ (1.4114*PBLACK)+ (0.169*PHISP)+ (-2.7371*PHOODVIOL)+ (-1.2966* PHOODMDD)+ (16.6962*PHOODDRGUSE())+ (-0.1982*PLIGHTDRK)+ (-4.6328* PHEAVYDRK)+ (-0.0982*PHOODHOM)+ (0.0968*PHOODSUIC)+ (0.0676*PHOODOWN)+ (23.7101*PHOODSUICATT)+ (-13.7515*PHOODSUICTHT)
3. P_DRUGUSER2 = exp(logit(P_ DRUGUSER2))/(1 + exp(logit(P_DRUGUSER2)))

where

P_NONDRK2, P_LIGHTDRK2, P_HEAVYDRK2 = probability of being a given drinking

status at current time step

P_DRUGUSER2 = probability of being a drug user at current time step

HOODINC1, HOODINC2 = dummy variables indicating average neighborhood income

< $40,000 (HOODINC1) or $40,000-$59,999 (HOODINC2) (≥ $60,000 is referent)

PHOODVIOL = proportion of agents residing in neighborhood who were victims of violence at

last time step

PHOODMDD = proportion of agents residing in neighborhood who had a depression diagnosis

at last time step

PLIGHTDRK = proportion of agents residing in neighborhood who were light/moderate drinkers

at last time step

PHEAVYDRK = proportion of agents residing in neighborhood who were heavy drinkers at last

time step

PHOODDRGUSE = proportion of agents residing in neighborhood who were drug users at last

time step

PHOODHOM = proportion of agents residing in neighborhood who were killed in a homicide at

last time step

PHOODSUIC = proportion of agents residing in neighborhood who committed suicide at last

time step

PHOODSUICATT = proportion of agents residing in neighborhood who attempted suicide at

last time step

PHOODSUICTHT = proportion of agents residing in neighborhood who thought about

committing suicide at last time step

PHOODOWN = proportion of agents residing in neighborhood who owned a firearm at last

time step

The final probabilities for each substance use status at the current time step are calculated as:

1. P_LIGHTDRK3 = (0.90*P_LIGHTDRK1) + (0.1*P_LIGHTDRK2)
2. P_HEAVYDRK3 = (0.90*P_ HEAVYDRK1) + (0.1*P_ HEAVYDRK2)
3. P_NONDRK3 = 1 – P_LIGHTDRK3 - P_HEAVYDRK3
4. P_DRUGUSER3 = (0.90*P_ DRUGUSER1) + (0.1*P_ DRUGUSER2)

After probabilities of substance use were calculated and agents were assigned a drinking status and a drug use status, substance abuse probabilities were calculated. If an agent was assigned to be a light drinker or a heavy drinker, a probability of having an alcohol abuse diagnosis was calculated. If an agent was assigned to be a drug user, a probability of having a drug abuse diagnosis was calculated. Both substance abuse probabilities were calculated from NCS-R data[15].

1. logit(P_ALCAB) = -4.733 + (0.0823*MALE)+ (1.3051*AGE1)+ (-0.1893*AGE2)+ (-0.731*AGE4)+ (-1.06*AGE5)+ (-14.387*AGE6)+ (-0.3828*BLACK)+ (-0.131*HISP)+ (0.3286*OTHER_RACE)+ (-0.8097*HS)+ (-0.9164*MOREHS)+ (0.6505*INC2)+ (0.3678*INC3)+ (0.5178*INC4)+ (-1.4982*MARRIED)+ (-0.7492*DIVSEPWID)+ (0.022*HISTVIOLPERP)+ (0.2439*HISTVIOLVICT)+ (-0.2731*LASTOWN)+ (0.1456*LASTCARRY)+ (1.6192*CURHEAVYDRK)+ (0.2844*CURDRUGUSER)+ (0.529*HISTDRUGAB)+ (2.7377*HISTALCAB)+ (0.1029*HISTMDD)+ (0.7973*HISTGAD)+ (-0.2885*HISTMANIA)+ (0.1199*HISTASB)+ (0.4234*HISTPSYCHO)+ (0.2521*HISTPTSD)+ (1.462*LASTIPVVICT)+ (-0.362*LASTIPVPERP)+ (0.249*HISTONHOSP)+ (0.5987*HISTIED)
2. logit(P_DRUGAB) = -1.5306+ (0.30*MALE)+ (-0.381*AGE1)+ (-1.428*AGE2)+ (-3.096*AGE4)+ (-17.5457*AGE5)+ (-16.4029*AGE6)+ (-0.0585*WHITE)+ (-0.8356*BLACK)+ (-1.41*OTHER_RACE)+ (0.4548*HS)+ (-0.7283*MOREHS)+ (-0.4734*INC2)+ (-0.5101*INC3)+ (-0.973*INC4)+ (0.2263*MARRIED)+ (0.0653*DIVSEPWID)+ (-0.3818*HISTVIOLPERP)+ (-0.7114*HISTVIOLVICT)+ (-0.4126*LASTOWN)+ (0.8354*CARRY)+ (-0.5987*CURLIGHTDRK)+ (0.3195*CURHEAVYDRK)+ (0.9289*HISTMDD)+ (0.5794*HISTGAD)+ (1.6376*HISTMANIA)+ (1.0744*HISTASB)+ (-0.363*HISTPSYCHO)+ (-1.9332*HISTPTSD)+ (0.144*HISTIED)+ (2.5239*IPVVICT)+ (-4.7904*IPVPERP)+ (0.1344*HISTONHOSP)+ (3.9168*AGE1*HISTDRUGAB)+ (2.9265*AGE2*HISTDRUGAB)+ (3.2902*AGE4*HISTDRUGAB )+ (18.9005*AGE5*HISTDRUGAB)+ (0.6839*AGE6*HISTDRUGAB)+ (0.6327*LASTOWN*LASTIED)
3. P_ ALCAB = exp(logit(P_ ALCAB))/(1 + exp(logit(P_ ALCAB)))
4. P_ DRUGAB = exp(logit(P_ DRUGAB))/(1 + exp(logit(P_ DRUGAB)))

Where

CURLIGHTDRK = dummy variable indicating if agent is a light drinker at current time step

CURHEAVYDRK = dummy variable indicating if agent is a heavy drinker at current time step

CURDRUGUSER = dummy variable indicating if agent is a drug user at current time step

After all substance use variables were set, the rest of the agent characteristics were calculated using the current time step’s drinking and drug use statuses, starting with firearm ownership and carrying statuses. The probability of firearm ownership and carrying was estimated from the National Comorbidity Study Replication (NCS-R), a nationally representative survey of US adults carried out between 2001 and 2003[15]. We restricted the calibration sample to respondents living in Census Metropolitan Statistical Areas to more accurately represent a city population. Note that ownership and carrying were calibrated separately; agents who did not own firearms could still carry a firearm in the model, representing the illicit firearms market as well as trading firearms outside of the legal market.

Probability of firearm ownership was re-estimated at each time step, based on sociodemographic characteristics, and history of: carrying status, violent victimization and perpetration, alcohol and drug use and abuse[16], each of the seven mental health disorders listed below, mental health treatment, and suicidal ideation and attempt[17,18]. Ownership at each timestep was also influenced by previous lifetime ownership, calibrated based on data from a survey conducted among US adults 18 years or older in 2013 by YouGov (http:// [www.yougov.com](http://www.yougov.com)). YouGov is a nonpartisan research firm that conducts nationally representative online surveys, often used for research[19,20]. History of a DUI/DWI arrest also influenced an agent’s probability of owning a firearm[21].

1. logit(P_OWN) = -3.245+ (0.5204*MALE)+ (1.8303*AGE1)+ (-0.3289*AGE2)+ (0.168*AGE3)+ (2.292*AGE5)+ (-0.7501*AGE6)+ (0.0245*WHITE)+ (-0.7501*HISP)+ (-0.555*OTHER_RACE)+ (0.5103*HS)+ (0.5516*MOREHS)+ (0.2989*INC2)+ (0.5264*INC3)+ (0.4768*INC4)+ (0.8079*MARRIED)+ (0.5529*DIVSEPWID)+ (0.2001*HISTVIOLPERP)+ (0.3022*HISTVIOLVICT)+ (0.0901*LASTCARRY)+ (0.0729*CURLIGHTDRK)+ (3.9186*CURHEAVYDRK)+ (-0.1088*CURDRUGUSER)+ (0.1749*HISTMDD)+ (0.0664*HISTGAD)+ (0.1033*HISTMANIA)+ (-0.1114*HISTASB)+ (0.4729*HISTIED)+ (0.2409*HISTPSYCHO)+ (-0.189*HISTPTSD)+ (-0.7744*HISTONHOSP)+ (0.1382*HISTALCAB)+ (-0.2274*HISTDRUGAB)+ (-0.7412*HISTSUICTHT)+ (0.1723*HISTSUICATT)+ (0.1408*BLACK*HISTASB)+ (0.5347*HISP*HISTASB)+ (-2.4526*OTHER_RACE*HISTASB)+ (0.3627*BLACK*HISTPSYCHO)+ (-2.2609*HISP*HISTPSYCHO)+ (-2.0108*OTHER_RACE*HISTPSYCHO)+ (-0.2676*MARRIED*HISTIED)+ (-1.6947*DIVSEPWID*HISTIED)+ (1.0371*MARRIED*HISTSUICTHT)+ (0.1169*DIVSEPWID*HISTSUICTHT)+ (-2.2877*HS*LASTCARRY)+ (-0.2633*MOREHS*LASTCARRY

INCREASEPROB ~ N(14.99, 4.37) = increase in probability if owned a firearm at last time step

DECREASEPROB ~ N(0.067, 0.02) = decrease in probability if did not own a firearm at last

time step

DUIOWN ~ N(1.79, 0.32) = increase in probability if had a DUI/DWI in the last year

1. PROB_OWN = [exp(logit(P_ OWN))/(1 + exp(logit(P_ OWN)))] × INCREASEPROB × DECREASEPROB × DUIOWN

A probability for carrying a firearm was then calculated using the current time step’s firearm ownership category. Firearm carrying status was based on sociodemographic characteristics, current firearm ownership status, history of: violent victimization and perpetration, alcohol and drug use[21], alcohol and drug abuse[16,21], each of the seven mental health disorders listed below, mental health treatment, and suicidal ideation and attempt[17,18]. If an agent was a young (18-24 years old) drug user[22] or if an agent had a history of a DUI/DWI arrest[21], the probability of carrying a firearm was increased.

1. logit(P_CARRY) = -5.9624+ (0.870*MALE)+ (-0.282*AGE1)+ (0.0585*AGE3)+ (-0.6159*AGE4)+ (-0.003*AGE5)+ (-1.0535*AGE6)+ (0.819*BLACK)+ (0.575*HISP)+ (0.3869*OTHER_RACE)+ (0.0721*HS)+ (-0.1154*MOREHS)+ (0.1902*INC2)+ (-0.0278*INC3)+ (0.368*INC4)+ (-0.6444*MARRIED)+ (-0.2799*DIVSEPWID)+ (0.6704*HISTVIOLPERP)+ (0.3696*HISTVIOLVICT)+ (0.5072*CURLIGHTDRK)+ (0.322*CURHEAVYDRK)+ (2.7341*CUROWN)+ (-0.5484*CURDRUGUSER)+ (0.1368*HISTMDD)+ (-0.1552*HISTGAD)+ (-0.0626*HISTMANIA)+ (-0.0298*HISTASB)+ (0.1383*LASTIED)+ (-0.0842*HISTPSYCHO)+ (0.1214*HISTPTSD)+ (-0.2299*HISTONHOSP)+ (0.174*CURALCAB)+ (0.2243*CURDRUGAB)+ (0.691*HISTSUICTHT)+ (-0.6069*HISTSUICATT)

YOUNGDRUGCAR ~ N(1.63, 0.5) = increase in probability if a young drug user

DUICARRY ~ N(2.1, 1.1) = increase in probability if had a DUI/DWI in the last year

1. PROB_CARRY = exp(logit(P_ CARRY))/(1 + exp(logit(P_ CARRY))) ×

YOUNGDRUGCAR × DUICARRY

Where

MALE = dummy variable indicating male gender (female is referent)

WHITE, BLACK, HISP, OTHER_RACE = dummy variables indicating race (reference is

different in each equation)

AGE1-AGE6 = dummy variables indicating age 18-24(AGE1), 25-34 (AGE2), 35-44 (AGE3),

45-54 (AGE4), 55-64 (AGE5), and ≥ 65 (AGE6) years (reference is different in each equation)

MARRIED, DIVSEPWID = dummy variables indicating marital status, married (MARRIED) or

divorced/separated/widowed (DIVSEPWID) (never married is referent)

HS, MOREHS = dummy variables indicating high school degree or equivalent (HS) or

more than high school education (MOREHS) (less than high school is referent)

INC2-INC4 = dummy variables indicating household income of $20,000-$39,999 (INC2),

$40,000-$74,999 (INC3), and ≥ $75,000 (INC4) (< $20,000 is referent)

HISTVIOLPERP = dummy variable indicating if agent was a perpetrator at a previous time step

HISTVIOLVICT = dummy variable indicating if agent was victimized at a previous time step

LASTVIOLPERP = dummy variable indicating if agent was a perpetrator at the last time step

LASTVIOLVICT = dummy variable indicating if agent was victimized at the last time step

CUROWN = dummy variable indicating if agent owns a firearm at current time step

CURCARRY = dummy variable indicating if agent carries a firearm at current time step

LASTCARRY = dummy variable indicating if agent carried a firearm at the last time step

HISTMDD = dummy variable indicating if agent had a diagnosis for depression (MDD) at a

previous time step

HISTGAD = dummy variable indicating if agent had a diagnosis for anxiety (GAD) at a previous

time step

HISTMANIA = dummy variable indicating if agent had a diagnosis for mania at a previous time

step

HISTASB = dummy variable indicating if agent had a diagnosis for antisocial behavior (ASB) at

a previous time step

HISTIED = dummy variable indicating if agent had a diagnosis for intermittent explosive

disorder (IED) at a previous time step

HISTPSYCHO = dummy variable indicating if agent had a diagnosis for psychosis (PSYCHO)

at a previous time step

HISTPTSD = dummy variable indicating if agent had a diagnosis for post-traumatic stress

disorder (PTSD) at a previous time step

HISTALCAB = dummy variable indicating if agent had a diagnosis for alcohol abuse (ALCAB)

at a previous time step

HISTDRUGAB = dummy variable indicating if agent had a diagnosis for drug abuse

(DRUGAB) at a previous time step

HISTONHOSP = dummy variable indicating if agent had an overnight hospitalization to treat a

mental health disorder (ONHOSP) at a previous time step

LASTMED= dummy variable indicating if agent was medicated (MED) for a mental health

disorder at the last time step

LASTOTREAT= dummy variable indicating if agent had some other treatment (OTREAT) for a

mental health disorder at the last time step

LASTIPVVICT = dummy variable indicating if agent was a victim of intimate partner violence

(IPV) at the last time step

LASTIPVPERP = dummy variable indicating if agent was a perpetrator of intimate partner

violence (IPV) at the last time step

CURDRUGUSER = dummy variable indicating if agent is a drug user at current time step

CURLIGHTDRK = dummy variable indicating if agent has been a light drinker at current time

step

CURHEAVYDRK = dummy variable indicating if agent has been a heavy drinker at current

time step

Agents also had the possibility of having one or more psychiatric disorders at each time step. Probabilities of having Major Depressive Disorder (MDD), General Anxiety Disorder (GAD), Antisocial Behavior (ASB), Intermittent Explosive Disorder (IED), Post Traumatic Stress disorder (PTSD), Mania, and Psychosis were also calculated from NCS-R[15]. Probability calculations were based on sociodemographic characteristics, current firearm ownership and carrying statuses, current alcohol and drug use[20], history of: violent victimization and perpetration[23], alcohol and drug abuse[20], each of the seven mental health disorders listed above, mental health treatment, and suicidal ideation and attempt[20,24]. Based on the probabilities, agents could be set to have one or more of any of the mental health disorders.

At each time-step, if an agent had a mental health disorder, they were also able to be in one or more treatments. The treatments were: overnight hospitalization, medication, and other treatments. Probabilities are also calculated from NCS-R[15]. and are based on sociodemographic characteristics, current firearm ownership and carrying statuses, current alcohol and drug use[20], history of: violent victimization and perpetration[23], alcohol and drug abuse[20], each of the seven mental health disorders listed above, mental health treatment, and suicidal ideation and attempt[20,24]. Based on the probabilities, agents who had at least one mental health disorder could be set to have one or more of any of the mental health disorders.

Lastly, some agents were set to be involuntary inpatients and outpatients. Probability of involuntary hospitalization was calculated based on NY State Office of Mental Health (OMH) data, and based on having an overnight hospitalization at that time step, race, age, and borough[25]. An agent’s probability of being an involuntary outpatient (involuntarily medicated or having another treatment) was calculated based on NY State OMH data, and based on having one of three conditions commonly associated with mental illness-related hospitalization (psychosis, mania, or MDD) at the current time step, race and age[26]. Based on these probabilities agents who had mental health treatment were set to be either involuntarily or voluntarily treated.

1. logit(P_MDD) = -3.008 + (-0.3051*MALE)+ (-0.8456*AGE1)+ (-1.2842*AGE2)+ (-2.2808*AGE3)+ (-2.442*AGE4)+ (-3.39*AGE6)+ (-0.0853*WHITE)+ (-0.3415*BLACK)+ (0.4954*OTHER_RACE)+ (0.0508*HS)+ (0.0789*MOREHS)+ (-0.2589*INC2)+ (-0.5051*INC3)+ (-0.7028*INC4)+ (-0.1309*MARRIED)+ (-0.0487*DIVSEPWID)+ (0.2729*HISTVIOLPERP)+ (0.3276*HISTVIOLVICT)+ (0.054*CURLIGHTDRK)+ (0.2626*CURHEAVYDRK)+ (0.217*CUROWN)+ (0.7032*CARRY)+ (0.3735*HISTALCAB)+ (0.2638*CURDRUGUSER)+ (-0.5167*HISTDRUGAB)+ (0.1088*HISTONHOSP)+ (0.3356*LASTMED)+ (1.5491*LASTOTREAT)+ (0.8189*HISTGAD)+ (-0.4759*HISTASB)+ (-17.5167*HISTMANIA)+ (0.4994*HISTPSYCHO)+ (0.496*HISTPTSD)+ (5.0288*AGE1*HISTMDD)+ (3.205*AGE2*HISTMDD)+ (3.2897*AGE3*HISTMDD)+ (2.7952*AGE4*HISTMDD)+ (2.4484*AGE6*HISTMDD)
2. PROB_ MDD = exp(logit(P_ MDD))/(1 + exp(logit(P_ MDD)))
3. logit(P_GAD) = -3.6337+ (-0.3825*MALE)+ (-1.6717*AGE1)+ (-3.457*AGE3)+ (-3.401*AGE4)+ (-2.801*AGE5)+ (-2.2751*AGE6)+ (-0.0051*WHITE)+ (-0.3299*BLACK)+ (-0.7251*HISP)+ (0.2631*HS)+ (0.0597*MOREHS)+ (-0.614*INC2)+ (-0.2119*INC3)+ (-1.0477*INC4)+ (0.4846*MARRIED)+ (1.0616*DIVSEPWID)+ (0.0850*HISTVIOLPERP)+ (0.0687*HISTVIOLVICT)+ (0.1322*CURLIGHTDRK)+ (0.3304*CURHEAVYDRK)+ (0.1111*CUROWN)+ (-0.1357*CARRY)+ (-0.08*CURDRUGUSER)+ (0.7227*HISTALCAB)+ (-0.1248*HISTDRUGAB)+ (0.0213*HISTMDD)+ (-0.6308*HISTMANIA)+ (0.3178*HISTASB)+ (-0.1131*HISTPSYCHO)+ (-0.7004*HISTPTSD)+ (-0.161*HISTONHOSP)+ (-0.23*LASTMED)+ (0.7986*LASTOTREAT)+ (-0.1403*HISTSUICTHT)+ (0.2587*HISTSUICATT)+ (5.6771*AGE1*HISTGAD)+ (4.9962*AGE3*HISTGAD)+ (4.8237*AGE4*HISTGAD)+ (4.2143*AGE5*HISTGAD)+ (2.8204*AGE6*HISTGAD)
4. PROB_ GAD = exp(logit(P_ GAD))/(1 + exp(logit(P_ GAD)))
5. logit(P_MANIA) = -8.41+ (-0.2558*MALE)+ (3.801*AGE1)+ (1.4769*AGE2)+ (1.063*AGE3)+ (-0.4890*AGE5)+ (-1.4636*AGE6) +(-0.18*WHITE)+ (0.1634*BLACK)+ (-0.0231*HISP)+ (0.0351*HS)+ (-0.496*MOREHS)+ (-0.0135*INC1)+ (0.0729*INC2)+ (0.1536*INC3)+ (-0.1643*MARRIED)+ (0.5137*DIVSEPWID)+ (0.4611*HISTVIOLPERP)+ (0.3873*HISTVIOLVICT)+ (0.1412*CURLIGHTDRK)+ (0.8715*CURHEAVYDRK)+ (0.38*CUROWN)+ (-0.7699*CARRY)+ (-0.00232*CURDRUGUSER)+ (0.0787*HISTALCAB)+ (0.188*HISTDRUGAB)+ (0.4949*HISTMDD)+ (0.7003*HISTGAD)+ (0.2432*HISTASB)+ (0.3686*HISTIED)+ (-0.8045*HISTPSYCHO)+ (0.4566*HISTPTSD)+ (0.4245*HISTSUICTHT)+ (0.0989*HISTSUICATT)+ (-0.5039*HISTONHOSP)+ (0.485*LASTMED)+ (1.2207*LASTOTREAT) + (3.4057*HISTMANIA)
6. PROB_ MANIA = exp(logit(P_ MANIA))/(1 + exp(logit(P_ MANIA)))
7. logit(P_ASB) = -22.5983+ (-0.1853*MALE)+ (16.5317*AGE1)+ (1.1035*AGE2)+ (-1.3144*AGE4)+ (0.2250*AGE5)+ (-1.8860*AGE6)+ (-0.065*WHITE)+ (-0.725*BLACK)+ (0.0514*OTHER_RACE)+ (0.5144*HS)+ (0.4365*MOREHS)+ (-0.4162*INC2)+ (0.8505*INC3)+ (-0.2378*INC4)+ (-0.6823*MARRIED)+ (0.0982*DIVSEPWID)+ (0.4671*HISTVIOLPERP)+ (0.0796*HISTVIOLVICT)+ (-1.471*CURLIGHTDRK)+ (0.3386*CURHEAVYDRK)+ (0.7541*CURDRUGUSER)+ (19.5544*HISTASB)
8. PROB_ ASB = exp(logit(P_ ASB))/(1 + exp(logit(P_ ASB)))
9. logit(P_IED) = -3.4804+ (-0.0222*MALE)+ (-0.350*AGE1)+ (-6.8402*AGE3)+ (-17.5448*AGE4)+ (-18.0705*AGE5)+ (-19.7037*AGE6)+ (-0.2612*WHITE)+ (-0.2107*HISP)+ (0.6691*OTHER_RACE)+ (-0.5009*HS)+ (-0.5924*MOREHS)+ (-0.1915*INC2)+ (-0.2099*INC3)+ (-0.2741*INC4)+ (0.8841*MARRIED)+ (0.3789*DIVSEPWID)+ (1.3369*HISTVIOLPERP)+ (0.672*HISTVIOLVICT)+ (0.1832*CURLIGHTDRK)+ (0.2729*CURHEAVYDRK)+ (-0.1611*CUROWN)+ (0.2167*CARRY)+ (0.068*CURDRUGUSER)+ (0.7559*HISTALCAB)+ (-0.3538*HISTDRUGAB)+ (0.5034*HISTMDD)+ (0.565*HISTGAD)+ (-1.0679*HISTMANIA)+ (-0.2366*HISTASB)+ (-0.4378*HISTPSYCHO)+ (-0.0514*HISTPTSD)+ (-0.9536*HISTONHOSP)+ (0.1319*HISTSUICTHT)+ (-0.3621*HISTSUICATT)+ (0.9219*LASTMED)+ (0.052*LASTOTREAT)+ (21.3177*AGE1*HISTIED)+ (6.6878*AGE3*HISTIED)+ (16.5317*AGE4*HISTIED)+ (16.8063*AGE5*HISTIED)+ (14.8929*AGE6*HISTIED)
10. PROB_ IED = exp(logit(P_ IED))/(1 + exp(logit(P_ IED)))
11. logit(P_PTSD) = -6.9155 + (-1.2706*MALE)+ (2.1254*AGE1)+ (0.631*AGE2)+ (0.6649*AGE4)+ (0.372*AGE5)+ (-3.6662*AGE6)+ (-0.1058*WHITE)+ (-0.145*BLACK)+ (-0.031*OTHER_RACE)+ (0.7289*HS)+ (-0.0205*MOREHS)+ (0.3095*INC2)+ (0.1461*INC3)+ (0.0599*INC4)+ (-0.6319*MARRIED)+ (-0.391*DIVSEPWID)+ (0.9468*HISTVIOLPERP)+ (0.0206*HISTVIOLVICT)+ (0.3667*CURLIGHTDRK)+ (0.4383*CURHEAVYDRK)+ (-0.1106*CUROWN)+ (1.8353*CARRY)+ (0.1581*CURDRUGUSER)+ (0.3518*HISTALCAB)+ (-0.363*HISTDRUGAB)+ (0.2977*HISTMDD)+ (0.8829*HISTGAD)+ (0.2809*HISTMANIA)+ (-0.1612*HISTASB)+ (-0.0661*HISTIED)+ (-0.2109*HISTPSYCHO)+ (-0.4431*HISTONHOSP)+ (0.738*HISTSUICTHT)+ (-0.2752*HISTSUICATT)+ (0.4063*LASTMED)+ (0.5035*LASTOTREAT)+ (3.8729*HISTPTSD)
12. PROB_ PTSD = exp(logit(P_ PTSD))/(1 + exp(logit(P_ PTSD)))
13. logit(P_PSYCHO) = -5.7520+ (0.4305*MALE)+ (0.3860*AGE1)+ (-1.927*AGE3)+ (-2.34*AGE4)+ (-3.4704*AGE5)+ (-0.9354*AGE6)+ (0.1337*WHITE)+ (0.7071*BLACK)+ (0.1324*OTHER_RACE)+ (-0.6234*HS)+ (-0.4306*MOREHS)+ (0.9929*INC1)+ (0.0509*INC2)+ (0.3903*INC3)+ (-1.1165*MARRIED)+ (-0.4994*DIVSEPWID)+ (0.332*LASTVIOLPERP)+ (0.5382*LASTVIOLVICT)+ (-0.6539*CURLIGHTDRK)+ (-1.1432*CURHEAVYDRK)+ (-0.852*CUROWN)+ (-0.8221*CARRY)+ (0.9989*HISTPTSD)+ (0.4339*HISTONHOSP)+ (-2.1385*LASTMED)+ (0.4287*CURDRUGUSER)+ (-0.2875*HISTMDD)+ (-0.4252*HISTGAD)+ (-1.3078*HISTASB)+ (-0.7623*HISTIED)+ (-1.6707*HISTMANIA)+ (5.7216*HISTPSYCHO)+ (0.3721*HISTSUICTHT)+ (-0.6746*HISTSUICATT)+ (0.819*LASTOTREAT)
14. PROB_ PSYCHO = exp(logit(P_ PSYCHO))/(1 + exp(logit(P_ PSYCHO)))
15. logit(P_ONHOSP) = -22.038+ (-1.9099*MALE)+ (-0.20*AGE2)+ (-0.727*AGE3)+ (-1.826*AGE4)+ (1.058*AGE5)+ (-17.0162*AGE6)+ (0.6076*BLACK)+ (-0.2581*HISP)+ (-16.9113*OTHER_RACE)+ (0.0787*HS)+ (-0.4737*MOREHS)+ (0.3181*INC2)+ (-1.1296*INC3)+ (-1.3494*INC4)+ (0.3078*MARRIED)+ (0.5716*DIVSEPWID)+ (0.7885*HISTVIOLPERP)+ (-0.9356*HISTVIOLVICT)+ (-0.2739*CURLIGHTDRK)+ (2.4456*CURHEAVYDRK)+ (-1.8357*CUROWN)+ (2.7991*CARRY)+ (-0.7281*CURDRUGUSER)+ (-0.7017*HISTALCAB)+ (1.4275*HISTDRUGAB)+ (0.7514*HISTMDD)+ (0.5081*HISTGAD)+ (1.0151*HISTMANIA)+ (-0.5093*HISTASB)+ (-1.1633*HISTIED)+ (-0.2551*HISTPSYCHO)+ (-0.0471*HISTPTSD)+ (-0.0406*HISTSUICATT)+ (-1.0145*LASTMED)+ (19.0763*LASTOTREAT)
16. PROB_ ONHOSP = exp(logit(P_ ONHOSP))/(1 + exp(logit(P_ ONHOSP)))
17. logit(P_MED) = -3.1022+ (0.4751*MALE)+ (-0.58*AGE1)+ (-0.84*AGE2)+ (-0.54*AGE3)+ (0.9389*AGE5)+ (2.3313*AGE6)+ (0.001*WHITE)+ (0.0067*BLACK)+ (-0.3225*HISP)+ (-0.8451*HS)+ (-1.0372*MOREHS)+ (0.6409*INC2)+ (0.5218*INC3)+ (0.742*INC4)+ (-0.9195*MARRIED)+ (-0.5427*DIVSEPWID)+ (-0.9633*HISTVIOLPERP)+ (0.3408*HISTVIOLVICT)+ (0.0374*CURLIGHTDRK)+ (-0.6184*CURHEAVYDRK)+ (-0.6278*CUROWN)+ (0.5483*CARRY)+ (1.4459*CURDRUGUSER)+ (0.5272*HISTMDD)+ (-0.0759*HISTGAD)+ (1.0165*HISTMANIA)+ (0.0374*HISTASB)+ (0.4183*HISTIED)+ (0.7204*HISTPSYCHO)+ (0.3385*HISTPTSD)+ (0.619*HISTALCAB)+ (0.3002*HISTDRUGAB)+ (-0.6795*HISTSUICATT)+ (-0.2681*HISTONHOSP)+ (0.6256*LASTOTREAT)
18. PROB_ MED = exp(logit(P_ MED))/(1 + exp(logit(P_ MED)))
19. logit(P_OTREAT) = -0.0035+ (-0.5003*MALE)+ (-0.6705*AGE1)+ (1.6503*AGE3)+ (2.7070*AGE4)+ (0.2011*AGE5)+ (1.8343*AGE6)+ (2.6040*WHITE)+ (-0.3503*BLACK)+ (-1.3914*OTHER_RACE)+ (0.2109*HS)+ (0.4624*MOREHS)+ (-0.1038*INC2)+ (-0.0918*INC3)+ (-0.1558*INC4)+ (-0.1899*MARRIED)+ (0.2575*DIVSEPWID)+ (0.0832*HISTVIOLPERP)+ (0.1546*HISTVIOLVICT)+ (-0.1535*CURLIGHTDRK)+ (0.00989*CURHEAVYDRK)+ (-0.2686*CUROWN)+ (0.0287*CARRY)+ (-0.0135*CURDRUGUSER)+ (0.1176*HISTMDD)+ (0.209*HISTGAD)+ (0.7969*HISTMANIA)+ (0.0241*HISTASB)+ (-0.0275*HISTIED)+ (-0.0797*HISTPSYCHO)+ (0.5028*HISTPTSD)+ (-0.3588*HISTALCAB)+ (0.4368*HISTDRUGAB)+ (0.00453*HISTSUICATT)+ (1.07*HISTONHOSP)+ (0.6367*LASTMED)
20. PROB_ OTREAT = exp(logit(P_ OTREAT))/(1 + exp(logit(P_ OTREAT)))

The following variables were calculated using current time-step drinking, drug use, firearm ownership and carrying statuses.

1. logit(P_IPVVICT) = -3.9726+ (-0.7533*MALE)+ (1.3069*AGE1)+ (1.90*AGE2)+ (-0.3362*AGE4)+ (-2.1776*AGE5)+ (-1.808*AGE6)+ (-0.6355*WHITE)+ (0.523*BLACK)+ (-0.187*OTHER_RACE)+ (0.1887*HS)+ (0.1959*MOREHS)+ (0.8257*INC2)+ (1.3368*INC3)+ (1.6631*INC4)+ (0.0959*HISTVIOLPERP)+ (0.1535*HISTVIOLVICT)+ (0.1924*CURLIGHTDRK)+ (0.4409*CURHEAVYDRK)+ (0.0631*CUROWN)+ (0.5109*CARRY)+ (-0.3914*CURDRUGUSER)+ (-0.2268*HISTALCAB)+ (0.0328*HISTDRUGAB)+ (-0.3889*HISTMDD)+ (0.3197*HISTGAD)+ (0.2412*HISTMANIA)+ (0.3154*HISTASB)+ (1.0514*HISTPSYCHO)+ (0.3642*HISTPTSD)+ (-0.254*HISTONHOSP)+ (0.6677*HISTIPVVICT)+ (1.0045*HISTIPVPERP)
2. PROB_ IPVVICT = exp(logit(P_ IPVVICT))/(1 + exp(logit(P_ IPVVICT)))
3. logit(P_IPVPERP) = -4.023+ (-0.9074*MALE)+ (2.382*AGE1)+ (1.3303*AGE2)+ (-0.266*AGE4)+ (-1.774*AGE5)+ (-0.78*AGE6)+ (0.6069*BLACK)+ (0.525*HISP)+ (0.2838*OTHER_RACE)+ (0.6053*HS)+ (0.4021*MOREHS)+ (0.6777*INC2)+ (1.4282*INC3)+ (1.825*INC4)+ (0.0607*HISTVIOLPERP)+ (0.1439*HISTVIOLVICT)+ (-0.2103*CURLIGHTDRK)+ (0.3546*CURHEAVYDRK)+ (0.0493*CUROWN)+ (-0.019*CARRY)+ (-0.1663*CURDRUGUSER)+ (0.142*HISTALCAB)+ (-0.1317*HISTDRUGAB)+ (-0.3464*HISTMDD)+ (0.1019*HISTGAD)+ (0.1708*HISTMANIA)+ (0.1481*HISTASB)+ (1.0239*HISTPSYCHO)+ (0.2514*HISTPTSD)+ (0.1934*HISTONHOSP)+ (0.3756*HISTIPVVICT)+ (0.8833*HISTIPVPERP)+ (0.5025*AGE1*HISTIPVVICT)+ (0.464*AGE2*HISTIPVVICT)+ (-0.0815*AGE4*HISTIPVVICT)+ (-0.0067*AGE5*HISTIPVVICT)+ (-2.1026*AGE6*HISTIPVVICT)+ (0.853*AGE1*HISTIPVPERP)+ (0.42*AGE2*HISTIPVPERP)+ (-0.2762*AGE4*HISTIPVPERP)+ (0.102*AGE5*HISTIPVPERP)+ (-0.0285*AGE6*HISTIPVPERP)
4. PROB_ IPVPERP = exp(logit(P_ IPVPERP))/(1 + exp(logit(P_ IPVPERP)))

Where

MALE = dummy variable indicating male gender (female is referent)

WHITE, BLACK, HISP, OTHER_RACE = dummy variables indicating race (reference is

different in each equation)

AGE1-AGE6 = dummy variables indicating age 18-24(AGE1), 25-34 (AGE2), 35-44 (AGE3),

45-54 (AGE4), 55-64 (AGE5), and ≥ 65 (AGE6) years (reference is different in each equation)

MARRIED, DIVSEPWID = dummy variables indicating marital status, married (MARRIED) or

divorced/separated/widowed (DIVSEPWID) (never married is referent)

HS, MOREHS = dummy variables indicating high school degree or equivalent (HS) or

more than high school education (MOREHS) (less than high school is referent)

INC2-INC4 = dummy variables indicating household income of $20,000-$39,999 (INC2),

$40,000-$74,999 (INC3), and ≥ $75,000 (INC4) (< $20,000 is referent)

HISTVIOLPERP = dummy variable indicating if agent was a perpetrator at a previous time step

HISTVIOLVICT = dummy variable indicating if agent was victimized at a previous time step

LASTVIOLPERP = dummy variable indicating if agent was a perpetrator at the last time step

LASTVIOLVICT = dummy variable indicating if agent was victimized at the last time step

CUROWN = dummy variable indicating if agent owns a firearm at current time step

CURCARRY = dummy variable indicating if agent carries a firearm at current time step

LASTCARRY = dummy variable indicating if agent carried a firearm at the last time step

HISTMDD = dummy variable indicating if agent had a diagnosis for depression (MDD) at a

previous time step

HISTGAD = dummy variable indicating if agent had a diagnosis for anxiety (GAD) at a previous

time step

HISTMANIA = dummy variable indicating if agent had a diagnosis for mania at a previous time

step

HISTASB = dummy variable indicating if agent had a diagnosis for antisocial behavior (ASB) at

a previous time step

HISTIED = dummy variable indicating if agent had a diagnosis for intermittent explosive

disorder (IED) at a previous time step

HISTPSYCHO = dummy variable indicating if agent had a diagnosis for psychosis (PSYCHO)

at a previous time step

HISTPTSD = dummy variable indicating if agent had a diagnosis for post-traumatic stress

disorder (PTSD) at a previous time step

HISTALCAB = dummy variable indicating if agent had a diagnosis for alcohol abuse (ALCAB)

at a previous time step

HISTDRUGAB = dummy variable indicating if agent had a diagnosis for drug abuse

(DRUGAB) at a previous time step

HISTONHOSP = dummy variable indicating if agent had an overnight hospitalization to treat a

mental health disorder (ONHOSP) at a previous time step

LASTMED= dummy variable indicating if agent was medicated (MED) for a mental health

disorder at the last time step

LASTOTREAT= dummy variable indicating if agent had some other treatment (OTREAT) for a

mental health disorder at the last time step

LASTIPVVICT = dummy variable indicating if agent was a victim of intimate partner violence

(IPV) at the last time step

LASTIPVPERP = dummy variable indicating if agent was a perpetrator of intimate partner

violence (IPV) at the last time step

CURDRUGUSER = dummy variable indicating if agent is a drug user at current time step

CURLIGHTDRK = dummy variable indicating if agent has been a light drinker at current time

step

CURHEAVYDRK = dummy variable indicating if agent has been a heavy drinker at current

time step

1. Movement to a new location: At each time step, each agent had a certain probability of

moving to a new neighborhood. Probabilities of moving over a one-year period were calculated from the Detroit Neighborhood Health Study (DNHS)[27] and calibrated so that patterns of mobility from/to high- and low-income neighborhoods by race/ethnicity were similar to patterns observed in the Panel Study of Income Dynamics[28]. The following logistic regression equation was used to calculate the probability of moving:

1. logit(P_MOVE) = -4.25 + (1.834*DURATION1) + (0.782*DURATION2) + (-0.147*DURATION3) + (0.855*INC1) + (0.706*INC2) + (0.597*INC3) + (1.307*LASTVICT)

Where

P_MOVE = probability of moving to a new neighborhood

DURATION1-DURATION3 = dummy variables indicating duration of residence in

current neighborhood of 0-5 years (DURATION1), 6-10 years (DURATION2), and 11-20 years (DURATION3) (>20 years is the referent)

INC1-INC3 = dummy variables indicating household income of <$20,000 (INC1), $20,000-

$39,999 (INC2), $40,000-$74,999 (INC3) (≥$75,000 is the referent)

LASTVICT = dummy variable indicating if agent was victimized at last time step

The calculated probability was further reduced (by half, on average) if the agent had committed violent perpetration at the last time step. The final probability of moving was thus calculated as follows:

REDUCEPROB ~ N(0.50, 0.025) = reduction in probability if perpetrated violence at last

time step

1. PROB_MOVE = [exp(logit(P_MOVE))/(1 + exp(logit(P_MOVE)))] ×

REDUCEPROB

Agents selected to move were then assigned to a new neighborhood based on their age, sex, race/ethnicity, and household composition.

Once agents moved around the grid, police officers were assigned to new locations in the physical space. Police officers were assigned to a random location within the neighborhood to which they were assigned at baseline (as described above in the Initialization section).

1. Potential violent victimization and perpetration, homicide and suicide: At each time

step, agents were calibrated to be violent victims or violent perpetrators. Probabilities of violent victimization and perpetration were calculated from NCS-R[15] based on sociodemographic characteristics, prior history of violent perpetration and victimization, history of mental health disorders (MDD, GAD, ASB, PTSD, IED, mania, psychosis) and mental health treatments (overnight hospitalizations)[23,29], drug and alcohol use and abuse[16,30,31], firearm ownership and carrying statuses[32], and neighborhood characteristics[33], and applied to the agents in the agent-based model. Violent perpetration is also based on history of any arrest based on NCS-R data[15].

Potential victims and perpetrators were identified at each time step. Potential perpetrators (i.e., those with a high predicted probability of perpetrating violence) searched a 15-cell radius around their location for potential victims (i.e., those with a high probability of being victimized); any such agents who had not already been victimized at that time step were matched to a perpetrator, unless a police officer was present within a 2-cell radius of the victim, in which case the potential victim was protected from violence.

A subset of violent incidents in the model were homicides, and a subset of those were deemed firearm related. Homicide probabilities were informed by data from the Underlying Cause of Death data available on CDC WONDER in NYC from 2008-2014[34]. Homicide and firearm related homicide probabilities were calculated based on race, sex, age, drug use, and heavy drinking status.[33] Firearm involvement was based on OCME data, calibrated based on race, sex, age, drug use, and heavy drinking status[33].

Agent’s died by suicide with or without a firearm based on OCME data[35]. Probabilities were calculated based on race, sex, age, drug use, and heavy drinking status[33,36]. Suicide was also influenced by history of suicide ideation and suicide attempt. Probabilities of suicide ideation and attempt are also calculated from NCS-R[15]. Probabilities are based on sociodemographic characteristics, current firearm ownership and carrying statuses, current alcohol and drug use[20], history of: violent victimization and perpetration[23], alcohol and drug abuse [20], each of the seven mental health disorders listed above, and mental health treatment [20,24].

Final probabilities were estimated from individual-, social network, and neighborhood-level characteristics, according to the procedure outlined below:

1. logit(P_VIOLVICT1) = -2.5151 + (-0.288*MALE)+ (0.9954*AGE1)+ (-1.3332*AGE2)+ (-15.6242*AGE4)+ (-2.47*AGE5)+ (-3.9974*AGE6)+ (-0.235*BLACK)+ (0.241*HISP)+ (-0.9554*OTHER_RACE)+ (-0.3094*HS)+ (-0.7514*MOREHS)+ (-0.1157*INC2)+ (-0.0406*INC3)+ (-0.3849*INC4)+ (-0.2677*MARRIED)+ (-0.2358*DIVSEPWID)+ (0.4726*CURLIGHTDRK)+ (0.3402*CURHEAVYDRK)+ (-0.1143*CUROWN)+ (0.1418*CARRY)+ (0.007*CURDRUGUSER)+ (0.2927*HISTMDD)+ (0.4271*HISTGAD)+ (0.3373*HISTMANIA)+ (0.3915*HISTASB)+ (-0.0631*LASTIED)+ (0.3851*HISTPSYCHO)+ (1.1302*HISTPTSD)+ (-0.265*HISTONHOSP)+ (-0.0271*HISTALCAB)+ (0.0584*HISTDRUGAB)+ (0.3158*AGE1*HISTVIOLPERP)+ (0.5058*AGE2*HISTVIOLPERP)+ (-0.5585*AGE4*HISTVIOLPERP)+ (0.1566*AGE5*HISTVIOLPERP)+ (-14.782*AGE6*HISTVIOLPERP)+ (1.1014*AGE1*HISTVIOLVICT)+ (2.1882*AGE2*HISTVIOLVICT)+ (15.8341*AGE4*HISTVIOLVICT)+ (2.2563*AGE5*HISTVIOLVICT)+ (3.135*AGE6*HISTVIOLVICT)
2. logit(P_VIOLPERP1) = -6.0701+ (0.3514*MALE)+ (1.722*AGE1)+ (-1.9207*AGE3)+ (-3.345*AGE4)+ (-0.8280*AGE5)+ (-15.0076*AGE6)+ (-0.426*WHITE)+ (0.2304*BLACK)+ (-0.489*OTHER_RACE)+ (0.2656*HS)+ (0.533*MOREHS)+ (-0.1661*INC2)+ (0.1439*INC3)+ (-0.3011*INC4)+ (0.00228*MARRIED)+ (0.2036*DIVSEPWID)+ (0.2727*CURLIGHTDRK)+ (0.1538*CURHEAVYDRK)+ (-0.0418*CUROWN)+ (0.4254*CARRY)+ (0.2011*CURDRUGUSER)+ (0.276*HISTMDD)+ (-0.2641*HISTGAD)+ (0.8836*HISTMANIA)+ (0.661*HISTASB)+ (1.2076*LASTIED)+ (-0.1159*HISTPSYCHO)+ (0.563*HISTPTSD)+ (-0.5451*HISTONHOSP)+ (0.5603*HISTALCAB)+ (0.3112*HISTDRUGAB)+ (1.4944*AGE1*HISTVIOLPERP)+ (1.0549*AGE3*HISTVIOLPERP)+ (3.7031*AGE4*HISTVIOLPERP)+ (0.6538*AGE5*HISTVIOLPERP)+ (15.4223*AGE6*HISTVIOLPERP)+ (0.3274*AGE1*HISTVIOLVICT)+ (1.2644*AGE3*HISTVIOLVICT)+ (0.0947*AGE4*HISTVIOLVICT)+ (0.8677*AGE5*HISTVIOLVICT)+ (-18.1121*AGE6*HISTVIOLVICT) +(0.4837*HISTANYARREST)
3. logit(P_SUICTHOUGHT) = -5.0751+ (-0.0701*MALE)+ (2.115*AGE1)+ (0.320*AGE2)+ (-0.138*AGE3)+ (-1.2712*AGE5)+ (-3.182*AGE6)+ (0.1388*WHITE)+ (-0.4167*BLACK)+ (0.4098*OTHER_RACE)+ (-0.1635*HS)+ (-0.0414*MOREHS)+ (0.00928*INC2)+ (-0.4942*INC3)+ (-0.4114*INC4)+ (-0.9099*MARRIED)+ (-0.4862*DIVSEPWID)+ (0.3532*HISTVIOLPERP)+ (-0.4121*HISTVIOLVICT)+ (0.1242*CURLIGHTDRK)+ (0.00627*CURHEAVYDRK)+ (-0.3953*CUROWN)+ (0.7845*CARRY)+ (0.5672*CURDRUGUSER)+ (-0.0945*HISTALCAB)+ (0.1302*HISTDRUGAB)+ (1.329*HISTMDD)+ (0.3655*HISTGAD)+ (0.6717*HISTMANIA)+ (0.3482*HISTASB)+ (0.0724*HISTPSYCHO)+ (0.4296*HISTPTSD)+ (2.6024*IPVVICT)+ (-0.767*IPVPERP)+ (-0.0178*HISTIED)+ (2.2732*HISTSUICATT)+ (-0.6577*AGE1*HISTSUICATT)+ (-0.5721*AGE2*HISTSUICATT)+ (-0.5179*AGE3*HISTSUICATT)+ (0.2568*AGE5*HISTSUICATT)+ (-0.3003*AGE6*HISTSUICATT)
4. PROB_ SUICTHOUGHT = exp(logit(P_ SUICTHOUGHT))/(1 + exp(logit(P_ SUICTHOUGHT)))
5. logit(P_SUICATTEMPT) = -10.3769+ (-0.0248*MALE)+ (2.1068*AGE1)+ (0.200*AGE3)+ (-3.076*AGE4)+ (-1.117*AGE5)+ (-1.4967*AGE6)+ (-0.3289*WHITE)+ (-0.2204*HISP)+ (0.4198*OTHER_RACE)+ (0.7464*HS)+ (0.4507*MOREHS)+ (0.4937*INC2)+ (0.4798*INC3)+ (-0.3092*INC4)+ (0.2459*MARRIED)+ (1.0006*DIVSEPWID)+ (1.1156*HISTVIOLPERP)+ (-0.49*HISTVIOLVICT)+ (0.7802*CURLIGHTDRK)+ (1.4256*CURHEAVYDRK)+ (-2.863*CUROWN)+ (1.1142*CARRY)+ (-0.3267*CURDRUGUSER)+ (-0.304*HISTALCAB)+ (0.8273*HISTDRUGAB)+ (0.0735*HISTMDD)+ (0.8131*HISTGAD)+ (0.2476*HISTMANIA)+ (-0.1391*HISTASB)+ (-0.3027*LASTIED)+ (-0.2811*HISTPSYCHO)+ (0.8814*HISTPTSD)+ (0.7769*IPVVICT)+ (-2.7876*IPVPERP)+ (1.8161*SuicAttcat1)+ (1.3242*SuicAttcat2)+ (3.0035*SuicAttcat3)+ (0.0257*HISTONHOSP)+ (6.5228* LASTSUICTHT)
6. logit(P_HOM1) = -10.093+ (-0.5552*WHITE)+ (1.354*BLACK)+ (-0.09*OTHER_RACE)+ (1.19*MALE)+ (0.220*AGE1)+ (-0.2295*AGE2)+ (0.081*AGE4)+ (0.4571*AGE5)+ (1.0082*AGE6)+ (2.0302*CURHEAVYDRK)+ (0.8045*CURDRUGUSER)
7. logit(P_GUNHOM1) = -0.2933+ (-0.3076*WHITE)+ (1.0355*BLACK)+ (-0.0908*OTHER_RACE)+ (1.9552*MALE)+ (1.6531*AGE1)+ (1.0033*AGE2)+ (-0.9957*AGE4)+ (-1.2134*AGE5)+ (-1.2592*AGE6)+ (-0.3241*CURHEAVYDRK)+ (0.2869*CURDRUGUSER)
8. logit(P_SUIC1) = -13.160+ (-0.194*WHITE)+ (-1.06*BLACK)+ (-1.4075*HISP)+ (1.9805*MALE)+ (-2.27*AGE1)+ (-0.6474*AGE2)+ (1.6816*AGE4)+ (0.9832*AGE5)+ (2.3529*AGE6)+ (2.0773*CURHEAVYDRK)+ (0.5008*CURDRUGUSER)
9. logit(P_ GUNSUIC1) = -5.2488+ (-0.8543*WHITE)+ (-0.851*HISP)+ (-2.2046*OTHER_RACE)+ (1.4033*MALE)+ (0.3483*AGE2)+ (0.6516*AGE3)+ (0.7421*AGE4)+ (0.2695*AGE5)+ (0.9113*AGE6)+ (0.3152*CURHEAVYDRK)+ (0.0116*CURDRUGUSER)

Where

MALE = dummy variable indicating male gender (female is referent)

WHITE, BLACK, HISP, OTHER_RACE = dummy variables indicating race (reference is

different in each equation)

AGE1-AGE6 = dummy variables indicating age 18-24(AGE1), 25-34 (AGE2), 35-44 (AGE3),

45-54 (AGE4), 55-64 (AGE5), and ≥ 65 (AGE6) years (reference is different in each equation)

MARRIED, DIVSEPWID = dummy variables indicating marital status, married (MARRIED) or

divorced/separated/widowed (DIVSEPWID) (never married is referent)

HS, MOREHS = dummy variables indicating high school degree or equivalent (HS) or

more than high school education (MOREHS) (less than high school is referent)

INC2-INC4 = dummy variables indicating household income of $20,000-$39,999 (INC2),

$40,000-$74,999 (INC3), and ≥ $75,000 (INC4) (< $20,000 is referent)

HISTVIOLPERP = dummy variable indicating if agent was a perpetrator at a previous time step

HISTVIOLVICT = dummy variable indicating if agent was victimized at a previous time step

LASTVIOLPERP = dummy variable indicating if agent was a perpetrator at the last time step

LASTVIOLVICT = dummy variable indicating if agent was victimized at the last time step

CUROWN = dummy variable indicating if agent owns a firearm at current time step

CURCARRY = dummy variable indicating if agent carries a firearm at current time step

LASTCARRY = dummy variable indicating if agent carried a firearm at the last time step

HISTMDD = dummy variable indicating if agent had a diagnosis for depression (MDD) at a

previous time step

HISTGAD = dummy variable indicating if agent had a diagnosis for anxiety (GAD) at a previous

time step

HISTMANIA = dummy variable indicating if agent had a diagnosis for mania at a previous time

step

HISTASB = dummy variable indicating if agent had a diagnosis for antisocial behavior (ASB) at

a previous time step

HISTIED = dummy variable indicating if agent had a diagnosis for intermittent explosive

disorder (IED) at a previous time step

HISTPSYCHO = dummy variable indicating if agent had a diagnosis for psychosis (PSYCHO)

at a previous time step

HISTPTSD = dummy variable indicating if agent had a diagnosis for post-traumatic stress

disorder (PTSD) at a previous time step

HISTALCAB = dummy variable indicating if agent had a diagnosis for alcohol abuse (ALCAB)

at a previous time step

HISTDRUGAB = dummy variable indicating if agent had a diagnosis for drug abuse

(DRUGAB) at a previous time step

HISTONHOSP = dummy variable indicating if agent had an overnight hospitalization to treat a

mental health disorder (ONHOSP) at a previous time step

LASTMED= dummy variable indicating if agent was medicated (MED) for a mental health

disorder at the last time step

LASTOTREAT= dummy variable indicating if agent had some other treatment (OTREAT) for a

mental health disorder at the last time step

LASTIPVVICT = dummy variable indicating if agent was a victim of intimate partner violence

(IPV) at the last time step

LASTIPVPERP = dummy variable indicating if agent was a perpetrator of intimate partner

violence (IPV) at the last time step

CURDRUGUSER = dummy variable indicating if agent is a drug user at current time step

CURLIGHTDRK = dummy variable indicating if agent has been a light drinker at current time

step

CURHEAVYDRK = dummy variable indicating if agent has been a heavy drinker at current

time step

HISTNUMTTEMPT1, HISTNUMTTEMPT2, HISTNUMTTEMPT3 = dummy variables if

agent has attempted suicide in a previous time step 1 time, 2 times or three or more times respectively

HISTSUICTHOUGHT = dummy variable if agent has thought about committing suicide in a

previous time step

HISTANYARREST = dummy variable if agent has ever had a preious arrest

The probabilities of homicide and suicide were further altered if the agent was in prison at the current time step, or had been in prison in the past [37–39]. If an agent was in prison the probability of homicide was decreased by 99%, and probability of suicide was increased by 250%. Once out of prison agents’ probabilities of homicide and suicide both increased as follows:

OUTPRISONHOM ~ N(5.65, 3.67) = increase in probability if agent was ever in prison.

OUTPRISONSUIC ~ N(2.4, 1.11) = increase in probability if agent was ever in prison.

Suicide and suicide attempt are also influenced by history of DUI/DWI, drug, and violent arrests based on NSDUH data[40].

DUIONSUICATT ~ N(1.3, 0.5) = increase in probability if agent had a DUI/DWI.

DRUGARRONSUICATT ~ N(2.7, 1.1) = increase in probability if agent had a drug arrest.

VIOLARRONSUICATT ~ N(3.5, 1.2) = increase in probability if agent had a violent arrest.

Suicide and firearm related suicide probabilities were increased for agents who had a current or past year increase of suicide attempt[41]. However, probability of firearm related suicde was decreased if there was a non-recent history of suicide attempt or any current mental health treatment.

SUICATTSUICINC ~ N(64.0, 2.0) = increase in probability if agent has recent history of suicide

attempt.

SUICATTSUICDEC ~ N(0.82, 0.10) = decrease in probability if agent has no recent history of

suicide attempt.

CURSUICATT_GUNSUIC ~ N(15.86, 4.0) = increase in probability if agent has recent history

of suicide attempt.

EVERSUICATT_GUNSUIC ~ N(0.62, 0.38) = decrease in probability if agent has history, but

not recent history, of suicide attempt.

CURMHTREAT_GUNSUIC ~ N(0.70, 0.10) = decrease in probability if agent has current

treatment for a mental health disorder.

The probability of suicide was also increased if the agent had an overnight hospitalization at the current or last time step and had a mental health diagnosis at the current time step [24].

MDDSUIC ~ N(13.0, 5.4) = increase in probability if agent has a depression diagnosis.

MANIASUIC ~ N(11.1, 5.05) = increase in probability if agent has a mania diagnosis.

PSYCHOSUIC ~ N(8.9, 3.95) = increase in probability if agent has a psychosis diagnosis.

SUBSUIC ~ N(6.6, 3.15) = increase in probability if agent has an alcohol abuse or drug abuse

diagnosis.

OTHERMHSUIC ~ N(13.0, 5.4) = increase in probability if agent has another mental health

diagnosis including anxiety, PTST, IED, and ASB.

Otherwise, if an agent was treated in some other way for a mental health disorder, their probability was also increased[42].

OMHTREATSUIC ~ N(1.2, 0.3) = increase in probability if agent has a some other treatment.

Suicide was also influenced by alcohol and drug abuse if the agent did not have an overnight hospitalization, based on NCS-R data[15,16].

ALCABSUIC ~ N(5.4, 3.5) = increase in probability if agent has an alcohol diagnosis and no

overnight hospitalization.

DRUGABSUIC ~ N(2.3, 1.5) = increase in probability if agent has a drug diagnosis and no

overnight hospitalization.

The probabilities of suicide, gun-related suicide, and gun-related homicide were increased if the agent owned a firearm at the current time step [32,43]. The probability of gun-related homicide was also increased if the agent carried a firear at the current timestep[44].

OWN_SUIC ~ N(3.5, 0.4) = increase in probability if agent owns a firearm.

OWN_GUNSUIC ~ N(16.89, 4.13) = increase in probability if agent owns a firearm.

OWN_GUNHOM ~ N(1.72, 0.31) = increase in probability if agent owns a firearm.

CARRY_GUNHOM ~ N(3.54, 2.00) = increase in probability if agent carrys a firearm.

Final individual level probabilities were calculated as such:

1. P_HOM1 = [exp(logit(P_ HOM1))/(1 + exp(logit(P_ HOM1)))] ×

OUTPRISONHOM

1. P_GUNHOM1 = [exp(logit(P_ GUNHOM1))/(1 + exp(logit(P_ GUNHOM1)))]

× OWN_GUNHOM × CARRY_GUNHOM

1. P_SUIC1 = [exp(logit(P_ SUIC1))/(1 + exp(logit(P_ SUIC1)))] ×

OUTPRISONSUIC × SUICATTSUICINC × SUICATTSUICDEC × MDDSUIC × MANIASUIC × PSYCHOSUIC × SUBSUIC × OTHERMHSUIC × OMHTREATSUIC × ALCABSUIC × DRUGABSUIC × OWN_SUIC × DUIONSUICATT × DRUGARRONSUICATT × VIOLARRONSUICATT

1. P_GUNSUIC1 = [exp(logit(P_ SUIC1))/(1 + exp(logit(P_ SUIC1)))]

OWN_ GUNSUIC × CURSUICATT_GUNSUIC × EVERSUICATT_GUNSUIC × CURMHTREAT_GUNSUIC

1. P_ SUICATTEMPT = exp(logit(P_ SUICATTEMPT))/(1 + exp(logit(P_

SUICATTEMPT))) × DUIONSUICATT × DRUGARRONSUICATT × VIOLARRONSUICATT

Probabilities calculated from these individual-level models accounted for 75% of the agent’s final probability for homicide, and 90% for violent victimization and perpetration, suicide, and gun-related homicide and suicide. Another 15% of the final probability of homicide was based on violence exposure among the agent’s social network members.

We started with the individual-level probability of homicide as calculated above; then if one of the agent’s friends was a victim of violence at the last time step, the index agent’s probability of homicide increased by 50%; if two or more friends were victims of violence, the index agent’s probability of homicide doubled. Given the lack of empiric data on the influence of violence exposure in one’s social network in general population samples[13], these parameters were subjected to sensitivity analyses to ensure that they were not unduly influencing model results. The network-influenced probability of homicide was then:

P_HOM2 = probability of homicide at current time step

Finally, the remaining 10% of agent’s final probabilities of violence were calculated from the following multilevel logistic regression equations estimated using neighborhood-level exposures aggregated from the WTC study[33] and Census data[5], to predict homicide, gun-related homicide, suicide, gun-related suicide, and non-fatal violent victimization. Specifically,

1. logit(P_HOM3) = -13.2286+ (0.3708*HOODINC1)+ (0.5528*HOODINC2)+

(1.3641*PHOODVIOL)+ (0.2172*PLIGHTDRK)+ (8.598*PHEAVYDRK)+ (-0.2063*PBLACK)+ (-0.2181*PHISP)+ (-0.1254*PFOREIGNBORN)+ (-0.0062*PMANAGER)+ (0.0186*PYOUNGMALE)+ (-0.3148*PSTABLE)+ (0.4012*PUNEMPLOYED)+ (1.4733*PFEMHHKIDS)+ (-1.7818*PHOODOWN)+ (0.0104*PHOODMDD)+ (2.3281*PHOODDRGUSE() )+ (-0.8229*PHOODSUICTHT)+ (3.492*PHOODSUICATT)

1. logit(P_GUNHOM2) = -14.5552+ (0.16*HOODINC1)+ (0.4849*HOODINC2)+

(1.8193*PHOODVIOL)+ (0.5155*PLIGHTDRK)+ (10.8059*PHEAVYDRK)+ (-0.0038*PBLACK)+ (3.2445*PHISP)+ (-3.3961*PFOREIGNBORN)+ (4.5924*PMANAGER)+ (-0.2696*PYOUNGMALE)+ (-0.1741*PSTABLE)+ (-0.302*PUNEMPLOYED)+ (-0.0102*PFEMHHKIDS)+ (0.0257*PHOODOWN)+ (-0.5156*PHOODMDD)+ (0.6421*PHOODDRGUSE() )+ (0.4955*PHOODSUICTHT)+ (-0.1618*PHOODSUICATT)

1. logit(P_SUIC2) = -8.8298+ (0.2664*HOODINC1)+ (0.1073*HOODINC2)+

(-0.4413*PHOODVIOL)+ (-0.194*PLIGHTDRK)+ (0.0874*PHEAVYDRK)+ (-0.4504*PBLACK)+ (0.0741*PHISP)+ (-0.0051*PFOREIGNBORN)+ (0.0168*PMANAGER)+ (-10.1527*PYOUNGMALE)+ (-0.4124*PSTABLE)+ (6.7542*PUNEMPLOYED)+ (-2.468*PFEMHHKIDS)+ (-0.0188*PHOODOWN)+ (0.2346*PHOODMDD)+ (0.3204*PHOODDRGUSE() )+ (-1.0965*PHOODSUICTHT)+ (0.6323*PHOODSUICATT)

1. logit(P_GUNSUIC2) = -7.2037+ (-0.06*HOODINC1)+ (0.0326*HOODINC2)+

(0.1722*PHOODVIOL)+ (0.6818*PLIGHTDRK)+ (0.0575*PHEAVYDRK)+ (-0.4026*PBLACK)+ (-0.1708*PHISP)+ (-0.0152*PFOREIGNBORN)+ (-0.0996*PMANAGER)+ (-15.8128*PYOUNGMALE)+ (-2.534*PSTABLE)+ (0.4754*PUNEMPLOYED)+ (-0.0855*PFEMHHKIDS)+ (-0.012*PHOODOWN)+ (0.1449*PHOODMDD)+ (0.2734*PHOODDRGUSE() )+ (-0.9335*PHOODSUICTHT)+ (-0.7388*PHOODSUICATT)

1. logit(P_VIOLVICT2) = -6.4282+ (0.1665*HOODINC1)+(0.2936*HOODINC2)+

(1.2259*PBLACK)+ (0.114*PHISP)+ (17.6647*PHOODVIOL)+ (1.5847*PHOODMDD)+ (4.1229*PHOODDRGUSE() )+ (1.3553*PLIGHTDRK)+ (0.6964*PHEAVYDRK)+ (-0.0012*PHOODHOM)+ (0.0906*PHOODSUIC)+ (-0.0168*PHOODOWN)+ (-9.534*PHOODSUICATT)+ (9.9015*PHOODSUICTHT)

Where

P_HOM3= probability of homicide at current time step

P_GUNHOM2= probability of gun-related homicide at current time step

P_SUIC2= probability of suicide at current time step

P_GUNSUIC2= probability of gun-related suicide at current time step

P_VIOLVICT2 = probability of violent victimization at current time step

P_PERP2 = probability of violent perpetration at current time step

PFOREIGNBORN = proportion of agents residing in neighborhood who were born

outside of the United States

PMANAGER = proportion of agents residing in neighborhood were employed in

professional or managerial occupations

PYOUNGMALE = proportion of agents residing in neighborhood who were under the

age of 25 years and male

PSTABLE = proportion of agents residing in neighborhood who had been living in the

neighborhood for at least one year

PUNEMPLOYED = proportion of agents residing in neighborhood who were

unemployed

PFEMHHKIDS = proportion of agents residing in neighborhood who lived in female-

headed households with children under the age of 18 years

The equation for violent perpetration (which was not available for NYC specifically and thus could not be linked easily to relevant neighborhood data) was modified from the victimization equation. Specifically, the intercept of the equation was decreased to account for the lower probability of perpetration, but associations between neighborhood characteristics and perpetration were assumed to be the same as those estimated for victimization.

1. logit(P_VIOLPERP2) = -2.1427+ (0.1665*HOODINC1)+

(0.2936*HOODINC2)+ (1.2259*PBLACK)+ (0.114*PHISP)+ (17.6647*PHOODVIOL)+ (1.5847*PHOODMDD)+ (4.1229*PHOODDRGUSE() )+ (1.3553*PLIGHTDRK)+ (0.6964*PHEAVYDRK)+ (-0.0012*PHOODHOM)+ (0.0906*PHOODSUIC)+ (-0.0168*PHOODOWN)+ (-9.534*PHOODSUICATT)+ (9.9015*PHOODSUICTHT)

Thus, the final probabilities of agent homicide, gun-related homicide, suicide, gun-related suicide, violent victimization and perpetration at each time step were calculated as follows.

1. P_HOM2 = exp(logit(P_HOM2))/(1 + exp(logit(P_HOM2)))
2. P_HOM3 = exp(logit(P_HOM3))/(1 + exp(logit(P_HOM3)))
3. PROB_HOM = (0.75*P_HOM1) + (0.15*P_HOM2) + (0.10*P_HOM3)
4. P_ GUNHOM2 = exp(logit(P_ GUNHOM2))/(1 + exp(logit(P_ GUNHOM2)))
5. PROB_GUNHOM = (0.90*P_ GUNHOM1) + (0.1*P_ GUNHOM2)
6. P_ SUIC2 = exp(logit(P_ SUIC2))/(1 + exp(logit(P_ SUIC2)))
7. PROB_SUIC = (0.90*P_ SUIC1) + (0.1*P_ SUIC2)
8. P_ GUNSUIC2 = exp(logit(P_ GUNSUIC2))/(1 + exp(logit(P_ GUNSUIC2)))
9. PROB_GUNSUIC = (0.90*P_ GUNSUIC1) + (0.1*P_ GUNSUIC2)
10. P_ VIOLVICT2 = exp(logit(P_ VIOLVICT2))/(1 + exp(logit(P_ VIOLVICT2)))
11. PROB_VIOLVICT = (0.90*P_ VIOLVICT1) + (0.1*P_ VIOLVICT2)
12. P_ VIOLPERP2 = exp(logit(P_ VIOLPERP2))/(1 + exp(logit(P_ VIOLPERP2)))
13. PROB_VIOLPERP = (0.90*P_ VIOLPERP1) + (0.1*P_ VIOLPERP2)
14. Actual violent incidents, homicides, and suicides: Once potential perpetrators and victims

are identified in the ABM, an additional process occurs to determine if a violent incident actually takes place. Specifically, potential perpetrators search a 15-cell radius around their location for potential victims who have not already been victimized at that time step. If a police officer is present within a 2-cell radius of the potential victim the violent act is prevented; however, if no police officer is present within the designated radius, the perpetrator engages with the victim. A subset of violent incidents in the model were then determined to be homicides. Once a violent act was designated, the model checked to see if the violent victim was a potential homicide victim. If so, the act of violence was also a homicide, and the violent perpetrator was also a homicide perpetrator. The model then checked to see if either the perpetrator or victim owns, carries, or has a friend who own’s a gun. If the perpetrator or victim does have access to a gun in one of these ways, and the homicide victim also was a potential firearm-related homicide victim, then the homicide was designated a firearm-related homicide.

Some agents commit suicide based on their previously calculated probabilities. Once an agent has been assigned to commit suicide, the model checked to see if the suicide was also a potential firearm-related suicide. If so, the suicide was designated a firearm-related suicide.

1. Arrests and sentencing The ABM allowed for four types of arrests and seven types of

convictions. Agents were assigned probabilities at each time step of having a violent arrest, a drug-related arrest, an alcohol-related arrest (DWI/DUI), or another type of arrest. Probabilities for each of type of arrest were calculated from 2004-2011 National Survey on Drug Use and Health (NSDUH) data[40]. We used parametric equations from NSDUH for probabilities of arrests, and when possible calibrated rates to match rates of arrest in based on 2011-2014 New York State Division of Criminal Justice Services (DCJS) data for New York City [45]. Probabilities were calculated based on sociodemographic characteristics, history of: suicidal ideation and attempt, MDD, GAD, mental health treatment[46], each of the arrest types, and current alcohol and drug use and abuse[46–48]. Based on these probabilities, agents were set to have any of the four types of arrests.

Once an agent was given an arrest status, an agent was then given a probability of their arrest being a misdemeanor or felony based on 2011-2014 New York State Division of Criminal Justice Services (DCJS) data[45]. DCJS is a criminal justice support agency; one of its main functions is collecting and maintaining statewide crime data. Once it was determined if an agent was arrested on a felony or misdemeanor charge, they were assigned a probability of conviction based DCJS data, on their race, sex, age, and borough[45].

A probability of having a DUI/DWI arrest was only calculated among agents who at the current time step were light drinkers, heavy drinkers, or drug users.

1. logit(P_DUIARREST) = -7.148+ (1.000*MALE)+ (-0.485*AGE1)+ (-0.043*AGE2)+ (-0.443*AGE4_4)+ (-0.0894*WHITE)+ (0.112*HISP)+ (0.2192*OTHER_RACE)+ (0.078*HS)+ (-0.2101*MOREHS)+ (0.0991*INC2)+ (-0.113*INC3)+ (-0.2073*INC4)+ (-0.5743*MARRIED)+ (0.5397*DIVSEPWID)+ (0.1792*HISTGAD)+ (-0.0224*HISTMDD)+ (0.0878*HISTSUICATT)+ (-0.0602*HISTSUICTHT)+ (0.0333*LASTONHOSP)+ (0.1711*LASTMED)+ (0.111*LASTOTREAT)+ (0.0793*CURDRUGAB)+ (0.7257*CURALCAB)+ (0.2463*CURLIGHTDRK)+ (0.6253*CURHEAVYDRK)+ (0.2876*CURDRUGUSER)+ (0.759*LASTDRUGARREST)+ (0.3063*LASTVIOLARREST)+ (0.5906*LASTOTHERARREST)

The probability of a DUI/DWI arrest was also increased if agent had previously been arrested for an alcohol-related felony or alcohol-related misdemeanor at a previous time step, based on handgun purchasing data from the California Department of Justice.

DUIFEL ~ N(1.65, 0.82) = increase in probability if agent has previous DUI/DWI felony arrest.

DUIMISD ~ N(6.22, 1.12) = increase in probability if agent has previous DUI/DWI

misdemeanor arrest.

1. P_ DUIARREST = exp(logit(P_ DUIARREST))/(1 + exp(logit(P_ DUIARREST)))] × DUIFEL × DUIMISD

Where

P_ DUIARREST = probability of having a DUI/DWI arrest at the current time step

HISTDRUGARREST = dummy variable indicating if agent had a drug arrest at a previous

time step

HISTVIOLARREST = dummy variable indicating if agent had a violent arrest at a previous

time step

HISTOTHERARREST = dummy variable indicating if agent had another type of arrest (not

DUI/DWI, drug, or violent arrest) at a previous time step

If an agent was assigned to be arrested for a DUI/DWI, a probability was then calculated for the agent to determine if it was a felony charge based on New York State Division of Criminal Justice Services (DCJS) data.

1. P_ DUIFEL = -4.4432+ (0.3005*MALE)+ (0.1688*BLACK)+ (0.0142*HISP)+

(-0.3229*OTHER_RACE)+ (0.1486*AGE2)+ (0.3134*AGE3)+ (0.4275*AGE4)+ (1.6403*BRONX)+ (1.6126*BROOKLYN)+ (1.7897*MANHATTAN)+ (1.7744*QUEENS)

Where

P_ DUIFEL = probability of having a DUI/DWI felony charge

BRONX, BROOKLYN, MANHATTAN, QUEENS = dummy variables indicating

borough agent lives in (Staten Island is referent)

Based on an agent’s probability of having a DUI/DWI felony charge, an agent is assigned to either have a DUI/DWI felony or a DUI/DWI misdemeanor charge. Next, each agent has a probability of being convicted of the charge (felony or misdemeanor) based on sex, race, age and borough. Agents who were charged with a misdemeanor were either convicted of a misdemeanor or not convicted, and agents who were charged with a felony were then either convicted on a felony charge, or were not convicted.

The probability of having a drug arrest was calculated among agents who at the current time step were drug users, with a 10% chance of being calculated among non-drug users based on NSDUH data and rates were calibrated to UCR data.

1. logit(P_DRUGARREST) = -4.5851+ (0.7212*MALE)+ (0.3409*AGE1)+ (0.3218*AGE4_3)+ (0.9448*AGE4_4)+ (-0.3986*WHITE)+ (0.3902*BLACK)+ (-0.8849*OTHER_RACE)+ (0.0494*HS)+ (-0.4749*MOREHS)+ (0.0925*INC2)+ (-0.0848*INC3)+ (-0.1841*INC4)+ (-0.7413*MARRIED)+ (0.2009*DIVSEPWID)+ (0.0969*HISTGAD)+ (0.0661*HISTMDD)+ (-0.1724*HISTSUICATT)+ (-0.0951*HISTSUICTHT)+ (-0.083*LASTONHOSP)+ (0.279*LASTMED)+ (-0.2121*LASTOTREAT)+ (0.5602*CURDRUGAB)+ (0.0638*CURALCAB)+ (-0.1117*CURLIGHTDRK)+ (0.2029*CURHEAVYDRK)+ (0.9453*CURDRUGUSER)+ (0.8289*LASTDUI)+ (0.4281*LASTVIOLARREST)+ (0.7104*LASTOTHERARREST)

The probability of a drug arrest was also increased if the agent had previously been convicted of a drug-related felony or drug-related misdemeanor at a previous time step based on California Department of Justice.

DRUGFEL ~ N(4.26, 1.08) = increase in probability if agent has previous drug felony arrest.

DRUGMISD ~ N(1.36, 0.72) = increase in probability if agent has previous drug misdemeanor

arrest.

1. P_ DRUGARREST = exp(logit(P_ DRUGARREST))/(1 + exp(logit(P_ DRUGARREST)))] × DRUGFEL × DRUGMISD

If an agent was assigned to have a drug arrest, a probability was then calculated for the agent to determine if it was a felony charge based on DCJS data:

1. P_ DRUGFEL = -0.9154+ (-0.1837*MALE)+ (0.3063*BLACK)+

(0.5255*HISP)+ (-0.1511*OTHER_RACE)+ (0.1139*AGE2)+ (0.0813*AGE3)+ (0.0573*AGE4)+ (-0.3269*BRONX)+ (-0.5659*BROOKLYN)+ (-0.0372* MANHATTAN)+ (-0.4734*QUEENS);

Where

P_ DRUGARREST = probability of having a drug-related arrest

P_ DRUGFEL = probability of having a drug felony charge

Based on an agent’s probability of having a drug felony charge, an agent is assigned to either have a drug felony or a drug misdemeanor charge, and follows the same path as an agent charged with a DUI/DWI felony or misdemeanor, and can either be convicted with a misdemeanor or felony, or not be convicted.

A probability of having a violent arrest was only calculated among agents who at the current time step were violent perpetrators or violent victims. The equation was based on NSDUH and rates were calibrated to UCR data.

1. logit(P_VIOLARREST) = -5.194+ (1.4835*MALE)+ (0.1262*AGE1)+ (-0.030*AGE2)+ (-0.5034*AGE4_4)+ (-0.563*WHITE)+ (1.1522*BLACK)+ (0.2521*OTHER_RACE)+ (0.104*HS)+ (-0.5774*MOREHS)+ (0.1059*INC2)+ (-0.0715*INC3)+ (-0.391*INC4)+ (-0.1373*MARRIED)+ (0.1873*DIVSEPWID)+ (0.0387*HISTGAD)+ (0.1749*HISTMDD)+ (0.1895*HISTSUICATT)+ (-0.1016*HISTSUICTHT)+ (0.5651*LASTONHOSP)+ (0.0591*LASTMED)+ (0.3286*LASTOTREAT)+ (-0.0715*CURDRUGAB)+ (0.2735*CURALCAB)+ (0.0149*CURLIGHTDRK)+ (0.4432*CURHEAVYDRK)+ (0.2772*CURDRUGUSER)+ (0.2753*LASTDUI)+ (0.3283*LASTDRUGARREST)+ (1.2306*LASTOTHERARREST)

The probability of a violent arrest was also increased if the agent had previously been convicted of a violent felony at a previous time step as shown below based on CHAD. The probability was also increased if the agent had a history of violent perpetration, history of mental health disorders, or owned a firearm at the current time step[49,50].

VIOLFEL ~ N(2.804, 0.43) = increase in probability if agent has previous violent felony arrest.

1. P_ VIOLARREST = exp(logit(P_ VIOLARREST))/(1 + exp(logit(P_ VIOLARREST)))] × VIOLFEL

Where

P_ VIOLARREST = probability of having a violent-related arrest

If an agent was assigned to have a violent arrest, it was assigned to be a felony arrest charge. Next, each agent had a probability of being convicted of the felony charge based on sex, race, age and borough. Agents were then either convicted on a felony charge, or were not convicted. A probability was also calculated for all agents of having another type of arrest based on NSDUH data[51].

1. logit(P_OTHERARREST) = -4.9057+ (1.033*MALE)+ (0.6704*AGE1)+ (0.1402*AGE2)+ (-0.4083*AGE4_4)+ (-0.6504*WHITE)+ (0.3774*BLACK)+ (-0.4782*OTHER_RACE)+ (0.0395*HS)+ (-0.3945*MOREHS)+ (0.0518*INC2)+ (-0.064*INC3)+ (-0.3107*INC4)+ (-0.3553*MARRIED)+ (0.3222*DIVSEPWID)+ (0.0276*HISTGAD)+ (0.0362*HISTMDD)+ (-0.0817*HISTSUICATT)+ (0.0979*HISTSUICTHT)+ (0.2195*LASTONHOSP)+ (0.1137*LASTMED)+ (0.141*LASTOTREAT)+ (0.0202*CURDRUGAB)+ (0.1319*CURALCAB)+ (-0.1006*CURLIGHTDRK)+ (0.0963*CURHEAVYDRK)+ (0.5333*CURDRUGUSER)+ (0.6526*LASTDUI)+ (0.6779*LASTDRUGARREST)+ (1.2773*LASTVIOLARREST)

The probability of another arrest was also increased if the agent had previously been convicted of another type of felony at a previous time step as shown below based on CHAD. The probability was also increased if the agent had a history of violent perpetration, history of mental health disorders, or owned a firearm at the current time step [49,50].

OTHERFEL ~ N(3.01, 0.49) = increase in probability if agent has previous other felony arrest.

1. P_ OTHERARREST = exp(logit(P_ OTHERARREST))/(1 + exp(logit(P_ OTHERARREST)))] × OTHERFEL

Where

P_ OTHERARREST = probability of having an arrest in another category.

If an agent was assigned to have another arrest, it was assigned to be a felony arrest charge. Next, each agent had a probability of being convicted of the felony charge based on sex, race, age and borough. Agents were then either convicted on a felony charge, or were not convicted.

Agents who had a felony conviction were assigned a probability of incarceration based on NYC Department of Corrections[52] population level and Justice Atlas[53] neighborhood level data. The probabilities were calculated based on age, race, sex, and community district. Based on those probabilities some agents were incarcerated, meaning that agents were removed from participating in the model for a certain number of years (time steps). They were no longer able to move around the model, own or carry a firearm, be involved in violence, or be arrested for another crime.

The probability of sentence length was calculated form Survey of Inmates in State and Federal Correctional Facilities (SISCF) data[54]. and based on sex, age, race, education, drug use, history of violent victimization, number of times incarcerated before, kind of arrest, and having friends who were incarcerated. Agents could be removed from the ABM grid for 1, 2, 5, 10, or 20 years. Once their sentence expired, and if they did not die during that time period, they were placed back on the grid in the location they were removed from.

1. logit(P_1YRSENT) = -3.0299+ (-0.8197*MALE)+ (1.0717*AGE1)+

(0.7419*AGE2)+ (0.7324*AGE3)+ (0.551*AGE4)+ (0.2605*AGE5)+ (0.0964*WHITE)+ (0.1681*BLACK)+ (0.0933*HISP)+ (0.0623*HS)+ (0.0326*MOREHS)+ (-0.0988* CURDRUGUSER)+ (-0.0295*FRDPRISON)+ (-0.1133*HISTVIOLVICT)+ (-0.0696* PRISON1)+ (-0.0208*PRISON2)+ (0.0268*PRISON3P)+ (0.464*HISTDUI_FEL)+

(-0.4939*HISTDRUG_FEL)+ (-1.304*HISTVIOL_FEL)

1. logit(P_2YRSENT) = -0.4691+ (-0.5665*MALE)+ (0.7596*AGE1)+

(0.245*AGE2)+ (0.1981*AGE3)+ (-0.0414*AGE4)+ (-0.0145*AGE5)+ (0.00843*WHITE)+ (-0.1436*BLACK)+ (0.1427*HISP)+ (0.1316*HS)+ (0.2488*MOREHS)+ (0.0119*CURDRUGUSER)+ (-0.1045*FRDPRISON)+ (-0.2452* HISTVIOLVICT)+ (0.00458*PRISON1)+ (-0.1616*PRISON2)+ (0.1748*PRISON3P)+ (0.2973*HISTDUI_FEL)+ (-0.3033*HISTDRUG_FEL)+ (-1.3917*HISTVIOL_FEL)

1. logit(P_5YRSENT) = -0.2164+ (0.0114*MALE)+ (0.6603*AGE1)+

(0.3903*AGE2)+ (0.1379*AGE3)+ (-0.0218*AGE4)+ (-0.0351*AGE5)+ (-0.08*WHITE)+ (-0.2788*BLACK)+ (0.00317*HISP)+ (-0.00407*HS)+ (0.0652*MOREHS)+ (0.0484*CURDRUGUSER)+ (-0.0817*FRDPRISON)+ (-0.0328*HISTVIOLVICT)+ (0.062*PRISON1)+ (0.1366*PRISON2)+ (0.1327*PRISON3P)+ (0.2947*HISTDUI_FEL)+ (0.2549*HISTDRUG_FEL)+ (-0.5372*HISTVIOL_FEL)

1. logit(P_15YRSENT) = -0.2223+ (0.5363*MALE)+ (-0.6735*AGE1)+

(-0.2303*AGE2)+ (-0.2207*AGE3)+ (-0.109*AGE4)+ (-0.2673*AGE5)+ (0.0736* WHITE)+ (0.1171*BLACK)+ (-0.0926*HISP)+ (0.0359*HS)+ (-0.1211*MOREHS)+ (0.0839*CURDRUGUSER)+ (0.0351*FRDPRISON)+ (0.0887*HISTVIOLVICT)+ (-0.1288*PRISON1)+ (-0.0665*PRISON2)+ (-0.1488* PRISON3P)+ (-0.7034* HISTDUI_FEL)+ (0.3891*HISTDRUG_FEL)+ (0.6825*HISTVIOL_FEL)

1. logit(P_35YRSENT) = -1.5182+ (0.6791*MALE)+ (-1.6631*AGE1)+

(-0.6912*AGE2)+ (-0.2556*AGE3)+ (0.0791*AGE4)+ (0.0965*AGE5)+ (-0.0537*WHITE)+ (0.2703*BLACK)+ (-0.1*HISP)+ (-0.2448*HS)+ (-0.1576*MOREHS)+ (0.0279*CURDRUGUSER)+ (0.2139*FRDPRISON)+ (0.1854*HISTVIOLVICT)+ (0.146*PRISON1)+ (-0.0164*PRISON2)+ (-0.2153*PRISON3P)+ (-1.4182* HISTDUI_FEL)+ (-0.0479*HISTDRUG_FEL)+ (1.9467*HISTVIOL_FEL)

1. P_ 1YRSENT = exp(logit(P_1YRSENT))/(1 + exp(logit(P_1YRSENT)) +

exp(logit(P_2YRSENT)) + exp(logit(P_5YRSENT)) + exp(logit(P_15YRSENT)) + exp(logit(P_35YRSENT)))]

1. P_ 2YRSENT = exp(logit(P_1YRSENT))/(1 + exp(logit(P_1YRSENT)) +

exp(logit(P_2YRSENT)) + exp(logit(P_5YRSENT)) + exp(logit(P_15YRSENT)) + exp(logit(P_35YRSENT)))]

1. P_ 5YRSENT = exp(logit(P_1YRSENT))/(1 + exp(logit(P_1YRSENT)) +

exp(logit(P_2YRSENT)) + exp(logit(P_5YRSENT)) + exp(logit(P_15YRSENT)) + exp(logit(P_35YRSENT)))]

1. P_ 15YRSENT = exp(logit(P_1YRSENT))/(1 + exp(logit(P_1YRSENT))

+ exp(logit(P_2YRSENT)) + exp(logit(P_5YRSENT)) + exp(logit(P_15YRSENT)) + exp(logit(P_35YRSENT)))]

1. P_ 35YRSENT = exp(logit(P_1YRSENT))/(1 + exp(logit(P_1YRSENT))

+ exp(logit(P_2YRSENT)) + exp(logit(P_5YRSENT)) + exp(logit(P_15YRSENT)) + exp(logit(P_35YRSENT)))]

Where

P_ 1YRSENT, P_ 2YRSENT, P_ 5YRSENT, P_ 15YRSENT, P_ 35YRSENT = probability

of being a given sentence length at current time step

FRDPRISON = dummy variable for if an agent has had a friend who has been in prison

PRISON1, PRISON2, PRISON3P = dummy variables for number of times agent has been

incarcerated (1, 2, or 3+)

Depending on which sentence length they received, an agent was then removed from the model grid for that number of time steps. Once the time was up, they would be placed back on the grid in the cell they were removed from.

1. Updates to neighborhood characteristics: At each time step, the average levels of income,

racial composition, drinking behaviors, firearm ownership, violent victimization, homicide, and suicide were recalculated for each neighborhood to account for experiences of violence and changes in drinking behaviors among neighborhood residents, as well as the changing agent composition of neighborhoods as individuals move to new locations in the physical space.

**Figure A1.** Diagram of relations between agent, social network, and neighborhood characteristics in the agent-based model

**Figure A2**. Flow diagram illustrating steps in model initialization

**Figure A3**. Flow diagram illustrating processes occurring at each step of the model

s**Table A1.** Agent, social network, and neighborhood parameters, values, data sources, and update rules

| **Parameter** | **Values** | **Data source(s)** | **Update rules** | **Reference** |
| --- | --- | --- | --- | --- |
| *Agent characteristics* | | | |  |
| Age | 18-85 (in single years) | Age, sex, and race/ethnicity were jointly assigned based on joint distributions from the 2010 Census, Summary File 1. | Age increases by one year at each time step. | Census 2010 |
| Sex | Male; Female |  | -- | Census 2010 |
| Race/ethnicity | White non-Hispanic; Black non-Hispanic; Hispanic; Other non-Hispanic |  | -- | Census 2010 |
| Educational attainment | < High school; High school degree or equivalent; > High school | Educational attainment was assigned based on age category, sex, and race/ethnicity, using American Community Survey data, 5-year estimates from 2012-2016. | -- | American Community Survey, 2012-2016 |
| Household income | < $20,000; $20,000-$39,999; $40,000-$74,999; ≥ $75,000 | Household income was assigned based on race/ethnicity, using American Community Survey data, 5-year estimates from 2006-2010 | -- | American Community Survey, 2006-2010 |
| Marital Status | Never married; Married; Divorced, separated, widowed | Marital status was assigned based on age category, sex, and race/ethnicity, using American Community Survey data, 5-year estimates from 2006-2010 | Marriage status could change every ten years. Agents can transition between marital categories except for married or Div/Sep/Wid to Never Married | American Community Survey, 2006-2010 |
| Duration of residence in  neighborhood | 0-40 (in single years) | Initial duration of residence was assigned based on age, using data from the 2000 Census, Summary File 3. | Duration of residence increases by one year at each time step. When agent moves to a new neighborhood, duration of residence is reset to 0. | Census 2000 |
| Probability of dying | 0-1 | Mortality probabilities were assigned based on age category, sex, and race/ethnicity, based on year 2010 mortality data from the NYC Department of Health and Mental Hygiene. | Mortality probabilities are updated when agent moves into an older age category. | NYC DOHMH, 2010 |
| Probability of moving  to a new neighborhood | 0-1 | Calculated from Detroit Neighborhood Health Study, based on household income, duration of residence in current neighborhood, and violent victimization at last time step; also adjusted for violent perpetration at last time step. | Recalculated at each time step. | [27] |
| Probability of violent  victimization | 0-1  Includes: In the past year beaten by another, mugged/held up/ threatened with a weapon, raped, sexually assaulted | Calculated from National Comorbidity Survey, based on individual age, sex, race/ethnicity, marital status, education, income, past year violent perpetration, prior history of violence victimization and perpetration, History of mental health disorders (depression, GAD, IED, ASB, mania, PTSD, psychosis), hist. of overnight hospitalization, alcohol and drug use, alcohol and drug abuse diagnoses, firearm ownership and carrying status, and neighborhood characteristics. | Recalculated at each time step. | - |
| Probability of violent  perpetration | 0-1  Includes: threaten someone with a weapon other than a gun, threaten someone with a gun, hurt someone so badly that they needed medical attention, purposefully either seriously injure, torture, or kill another person | Calculated from National Comorbidity Survey, based on individual age, sex, race/ethnicity, marital status, education, income, past year violent victimization, prior history of violence victimization and perpetration, History of mental health disorders (depression, GAD, IED, ASB, mania, PTSD, psychosis), hist. of overnight hospitalization, alcohol and drug use, alcohol and drug abuse diagnoses, firearm ownership and carrying status, and neighborhood characteristics. | Recalculated at each time step. | -- |
| Witnessed violence | No; Yes | Determined by proximity to victim of violence. | Reassessed at each time step. | Emerges from model |
| Probability of mental illness: DEPRESSION | 0-1  DSM-IV Major Depressive Disorder with hierarchy (12 month) | Calculated from National Comorbidity Survey, based on individual age, sex, race/ethnicity, marital status, education, income, past year violent victimization and perpetration, prior history of violence victimization and perpetration, History of mental health disorders (GAD, IED, ASB, mania, PTSD, psychosis), hist. of overnight hospitalization, alcohol and drug use, alcohol and drug abuse diagnoses, firearm ownership and carrying status, past year medication use, other past year treatment, and neighborhood characteristics | Recalculated at each time step. | -- |
| Probability of mental illness: GENERAL ANXIETY DISORDER | 0-1  DSM-IV Generalized Anxiety Disorder with hierarchy (12 month) | Calculated from National Comorbidity Survey, based on individual age, sex, race/ethnicity, marital status, education, income, past year violent victimization and perpetration, prior history of violence victimization and perpetration, History of mental health disorders (Depression, IED, ASB, mania, PTSD, psychosis), hist. of overnight hospitalization, alcohol and drug use, alcohol and drug abuse diagnoses, firearm ownership and carrying status, past year medication use, other past year treatment, ever attempted suicide, any suicide ideation | Recalculated at each time step. | -- |
| Probability of mental illness: MANIA | 0-1  DSM-IV Mania (12 month) | Calculated from National Comorbidity Survey, based on individual age, sex, race/ethnicity, marital status, education, income, past year violent victimization and perpetration, prior history of violence victimization and perpetration, History of mental health disorders (Depression, IED, ASB, GAD, PTSD, psychosis), hist. of overnight hospitalization, alcohol and drug use, alcohol and drug abuse diagnoses, firearm ownership and carrying status, past year medication use, other past year treatment, ever attempted suicide, any suicide ideation | Recalculated at each time step. | -- |
| Probability of mental illness: ANTISOCIAL BEHAVIOR | 0-1  DSM-IV Oppositional Defiant Disorder (12 month) | Calculated from National Comorbidity Survey, based on individual age, sex, race/ethnicity, marital status, education, income, past year violent victimization and perpetration, prior history of violence victimization and perpetration, History of mental health disorders (Depression, IED, Mania, GAD, PTSD, psychosis), hist. of overnight hospitalization, alcohol and drug use, alcohol and drug abuse diagnoses, firearm ownership and carrying status, past year medication use, other past year treatment, ever attempted suicide, any suicide ideation | Recalculated at each time step. | -- |
| Probability of mental illness: PSYCHOSIS | 0-1  Includes if any of the following have happened in the past 12 months: seeing a vision, hearing voices, believe there was a plot against you, or believing that a force: inserted strange thoughts, stole thoughts out of your mind, tried to communicate with you through special signals | Calculated from National Comorbidity Survey, based on individual age, sex, race/ethnicity, marital status, education, income, past year violent victimization and perpetration, prior history of violence victimization and perpetration, History of mental health disorders (Depression, IED, Mania, GAD, PTSD, ASB), hist. of overnight hospitalization, alcohol and drug use, alcohol and drug abuse diagnoses, firearm ownership and carrying status, past year medication use, other past year treatment, ever attempted suicide, any suicide ideation | Recalculated at each time step. | -- |
| Probability of mental illness: PTSD | 0-1  DSM-IV Post Traumatic Stress Disorder (12 month) | Calculated from National Comorbidity Survey, based on individual age, sex, race/ethnicity, marital status, education, income, past year violent victimization and perpetration, prior history of violence victimization and perpetration, History of mental health disorders (Depression, IED, Mania, GAD, psychosis, ASB), hist. of overnight hospitalization, alcohol and drug use, alcohol and drug abuse diagnoses, firearm ownership and carrying status, past year medication use, other past year treatment, ever attempted suicide, any suicide ideation | Recalculated at each time step. | -- |
| Probability of mental illness: OVERNIGHT HOSPITALIZATION – assumed voluntary | 0-1  Been admitted for an overnight stay for problems with emotions, nerves or mental health | Calculated from National Comorbidity Survey, based on individual age, sex, race/ethnicity, marital status, education, income, past year violent victimization and perpetration, prior history of violence victimization and perpetration, History of mental health disorders (Depression, IED, Mania, GAD, psychosis, ASB, PTSD), alcohol and drug use, alcohol and drug abuse diagnoses, firearm ownership and carrying status, past year medication use, other past year treatment, ever attempted suicide before past year | Recalculated at each time step. | -- |
| Probability of mental illness: OVERNIGHT HOSPITALIZATION - involuntary | 0-1  Been admitted, involuntarily for an overnight stay for problems with emotions, nerves or mental health | Calculated as a subset from the overnight hospitalization parameter, based on age, race, and NYC borough based on the New York State Office of Mental Health. | Recalculated at each time step. | Patient Characteristics Survey [25] |
| Probability of mental illness: MEDICATION USE | 0-1  Took medication in the past year for problems with emotions, substance use, energy, concentration, sleep, or ability to cope with stress | Calculated from National Comorbidity Survey, based on individual age, sex, race/ethnicity, marital status, education, income, past year violent victimization and perpetration, prior history of violence victimization and perpetration, History of mental health disorders (Depression, IED, Mania, GAD, psychosis, ASB, PTSD), hist. of overnight hospitalization, alcohol and drug use, alcohol and drug abuse diagnoses, firearm ownership and carrying status, other past year treatment, ever attempted suicide before the past year | Recalculated at each time step. | -- |
| Probability of mental illness: OTHER TREATMENT | 0-1  Utilized a different treatment in the past year, including: internet support group, self-help group, hotline, counseling, social worker, psychologist, psychiatrist, other medical professional, religious advisor, healer | Calculated from National Comorbidity Survey, based on individual age, sex, race/ethnicity, marital status, education, income, past year violent victimization and perpetration, prior history of violence victimization and perpetration, History of mental health disorders (Depression, IED, Mania, GAD, psychosis, ASB, PTSD), hist. of overnight hospitalization, alcohol and drug use, alcohol and drug abuse diagnoses, firearm ownership and carrying status, past year medication use, ever attempted suicide before past year | Recalculated at each time step. | -- |
| Probability of mental illness: OUTPATIENT - involuntary | 0 -1 was involuntarily treated: medication or other form of treatment | Calculated from the New York State Office of Mental Health, based on sex and race. Taken as a subset from those who received *medication* or *other treatment* and shown above. | Recalculated at each time step. | New York State Assisted Outpatient Treatment Program Evaluation [26] |
| Probability of Intimate Partner Violence (IPV) Victimization | 0-1  Had any of the following done to you by a partner: Pushed, grabbed or shoved, threw something, slapped or hit, beat up, choked, burned or scalded, threatened with a knife or gun. | Calculated from National Comorbidity Survey, based on individual age, sex, race/ethnicity, marital status, education, income, past year violent victimization and perpetration, prior history of violence victimization and perpetration, History of mental health disorders (Depression, IED, Mania, GAD, psychosis, ASB, PTSD), hist. of overnight hospitalization, alcohol and drug use, alcohol and drug abuse diagnoses, firearm ownership and carrying status, past year medication use, history of IPV perpetration | Recalculated at each time step. | -- |
| Probability of Intimate Partner Violence (IPV) Perpetration | 0-1  Done any of the following to a partner: Pushed, grabbed or shoved, threw something, slapped or hit, beat up, choked, burned or scalded, threatened with a knife or gun. | Calculated from National Comorbidity Survey, based on individual age, sex, race/ethnicity, marital status, education, income, past year violent victimization and perpetration, prior history of violence victimization and perpetration, History of mental health disorders (Depression, IED, Mania, GAD, psychosis, ASB, PTSD), hist. of overnight hospitalization, alcohol and drug use, alcohol and drug abuse diagnoses, firearm ownership and carrying status, past year medication use, history of IPV victimization | Recalculated at each time step. | -- |
| Probability of Firearm Ownership | 0 -1  Own vs. not own a firearm | Calculated from National Comorbidity Survey, based on individual age, sex, race/ethnicity, marital status, education, income, past year violent victimization and perpetration, prior history of violence victimization and perpetration, History of mental health disorders (Depression, IED, Mania, GAD, psychosis, ASB, PTSD), hist. of overnight hospitalization, alcohol and drug use, alcohol and drug abuse diagnoses, firearm carrying status, ever attempted suicide, ever had suicide ideation | Recalculated at each time step. | -- |
| Probability of Carrying a Firearm | 0 -1  Carries vs. Does not Carry a firearm | Calculated from National Comorbidity Survey, based on individual age, sex, race/ethnicity, marital status, education, income, past year violent victimization and perpetration, prior history of violence victimization and perpetration, History of mental health disorders (Depression, IED, Mania, GAD, psychosis, ASB, PTSD), hist. of overnight hospitalization, alcohol and drug use, alcohol and drug abuse diagnoses, firearm ownership, ever attempted suicide, ever had suicide ideation | Recalculated at each time step. | -- |
| Probability of Suicide Ideation | 0 -1  Seriously thought about committing suicide | Calculated from National Comorbidity Survey, based on individual age, sex, race/ethnicity, marital status, education, income, past year violent victimization and perpetration, prior history of violence victimization and perpetration, History of mental health disorders (Depression, IED, Mania, GAD, psychosis, ASB, PTSD), hist. of overnight hospitalization, alcohol and drug use, alcohol and drug abuse diagnoses, firearm ownership, # of past suicide attempts, IPV victimization and perpetration | Recalculated at each time step. | -- |
| Probability of Suicide Attempt | 0 -1  Attempted suicide | Calculated from National Comorbidity Survey, based on individual age, sex, race/ethnicity, marital status, education, income, past year violent victimization and perpetration, prior history of violence victimization and perpetration, History of mental health disorders (Depression, IED, Mania, GAD, psychosis, ASB, PTSD), hist. of overnight hospitalization, alcohol and drug use, alcohol and drug abuse diagnoses, firearm ownership, # of past suicide attempts, IPV victimization and perpetration | Recalculated at each time step. | -- |
| Probability of Alcohol Use | 0 -1 light drinker, 0 -1 heavy drinker  No use, light/moderate user, heavy user | Calculated from National Comorbidity Survey, based on individual age, sex, race/ethnicity, marital status, education, income, past year violent victimization and perpetration, prior history of violence victimization and perpetration, History of mental health disorders (Depression, IED, Mania, GAD, psychosis, ASB, PTSD), hist. of overnight hospitalization, drug use, alcohol and drug abuse diagnoses, firearm ownership, # of past suicide attempts, IPV victimization and perpetration | Recalculated at each time step. | -- |
| Probability of Drug Use | 0 -1  Drug user v. Not a drug user | Calculated from National Comorbidity Survey, based on individual age, sex, race/ethnicity, marital status, education, income, past year violent victimization and perpetration, prior history of violence victimization and perpetration, History of mental health disorders (Depression, IED, Mania, GAD, psychosis, ASB, PTSD), hist. of overnight hospitalization, alcohol use, alcohol and drug abuse diagnoses, firearm ownership, # of past suicide attempts, IPV victimization and perpetration | Recalculated at each time step. | -- |
| Probability of Alcohol Abuse | 0 -1  Among current drinks (light or heavy) | Calculated from National Comorbidity Survey, based on individual age, sex, race/ethnicity, marital status, education, income, past year violent victimization and perpetration, prior history of violence victimization and perpetration, History of mental health disorders (Depression, IED, Mania, GAD, psychosis, ASB, PTSD), hist. of overnight hospitalization, level of alcohol use and drug use, alcohol and drug abuse diagnoses, firearm ownership, # of past suicide attempts, IPV victimization and perpetration | Recalculated at each time step. | -- |
| Probability of Drug abuse | 0 -1  Among current drug users | Calculated from National Comorbidity Survey, based on individual age, sex, race/ethnicity, marital status, education, income, past year violent victimization and perpetration, prior history of violence victimization and perpetration, History of mental health disorders (Depression, IED, Mania, GAD, psychosis, ASB, PTSD), hist. of overnight hospitalization, alcohol use, alcohol and drug abuse diagnoses, firearm ownership, # of past suicide attempts, IPV victimization and perpetration | Recalculated at each time step. | -- |
| Probability of Committing Suicide | 0 – 1 | Suicide probabilities were assigned based on age category, sex, and race/ethnicity, based on the average mortality data from 2008-2014 CDC WONDER Underlying Cause of Death, and alcohol and drug use based on National Survey on Drug Use and Health, and Neighborhood characteristics. Also influenced by firearm ownership based on a meta-Analysis conducted by Miller et al. Influenced by mental health diagnoses based on meta-analyses conducted by Cavanagh et al. | Recalculated at each time step. | NYC DOHMH, Miller et Al.[43], Cavanagh et al.[55] |
| Probability of Committing Suicide with a Firearm | 0 – 1 | Suicide probabilities were assigned based on age category, sex, and race/ethnicity, based on the average mortality data from 2008-2014 CDC WONDER Underlying Cause of Death, and alcohol and drug use based on National Survey on Drug Use and Health, and Neighborhood characteristics. Also influenced by firearm ownership based on a meta-Analysis conducted by Miller et al. Influenced by mental health diagnoses based on meta-analyses conducted by Cavanagh et al. Influenced by mental health treatment and past suicide attempt based on Kaplan et al. | Recalculated at each time step. | NYC DOHMH, Miller et Al.[43], Cavanagh et al.[55], Kaplan et al.[56] |
| Probability of being a Victim of Homicide | 0 – 1 | Homicide probabilities were assigned based on age category, sex, and race/ethnicity, based on the average mortality data from 2008-2014 CDC WONDER Underlying Cause of Death, and alcohol and drug use based on National Survey on Drug Use and Health, and Neighborhood characteristics. Influenced by social network as well, based on Tracy et Al. | Recalculated at each time step. | CDC WONDER, Tracy et al. [13] |
| Probability of being a Victim of Homicide with a Firearm | 0 – 1 | Homicide probabilities were assigned based on age category, sex, and race/ethnicity, based on the average mortality data from 2008-2014 CDC WONDER Underlying Cause of Death, and alcohol and drug use based on National Survey on Drug Use and Health, and Neighborhood characteristics. Influenced by social network as well, based on Tracy et Al. and Papachristos et al. | Recalculated at each time step. | CDC WONDER, Tracy et Al.[13], Papachristos et Al. [57] |
| Probability of having a Drug Related Arrest | 0 – 1 | Calculated from National Survey on Drug Use and Health based on individual age, sex, race/ethnicity, marital status, education, income, History of mental health disorders (Depression, GAD), hist. of overnight hospitalization, alcohol use, alcohol and drug abuse diagnoses, hist. or suicide attempt and ideation, hist. of medication, hist. of other treatment, drug use and abuse, alcohol use and abuse, history of violent, alcohol related, and other arrests. Also influenced by handgun purchasing data from the California Department of Justice: history of drug related felonies and misdemeanors.  Calibrated to rates from New York State Division of Criminal Justice Services 2011-2014 | Recalculated at each time step. | DCJS |
| Probability of having a DWI/DUI (alcohol related arrest) | 0 – 1 | Calculated from National Survey on Drug Use and Health based on individual age, sex, race/ethnicity, marital status, education, income, History of overnight hospitalization, alcohol use, alcohol and drug abuse diagnoses, hist. or suicide attempt and ideation, hist. of medication, hist. of other treatment, drug use and abuse, alcohol use and abuse, history of violent, drug-related, and other arrests. Also influenced by handgun purchasing data from the California Department of Justice: history of alcohol-related felonies and misdemeanors. Calibrated to rates from New York State Division of Criminal Justice Services 2011-2014 | Recalculated at each time step. | DCJS |
| Probability of having a Violence Related arrest | 0 – 1 | Calculated from National Survey on Drug Use and Health based on individual age, sex, race/ethnicity, marital status, education, income, History of mental health disorders (Depression, GAD), hist. of overnight hospitalization, alcohol use, alcohol and drug abuse diagnoses, hist. or suicide attempt and ideation, hist. of medication, hist. of other treatment, drug use and abuse, alcohol use and abuse, history of drug-related, alcohol-related, and other arrests. Influenced by gun ownership based on Cook and Ludwig, and by mental health based on Coker et Al. Also influenced by handgun purchasing data from the California Department of Justice: history of violent felonies.  Calibrated to rates from New York State Division of Criminal Justice Services 2011-2014 | Recalculated at each time step. | DCJS, Cook and Ludwig[50], Coker et al.[49] |
| Probability of having any other arrest | 0 – 1 | Calculated from National Survey on Drug Use and Health based on individual age, sex, race/ethnicity, marital status, education, income, History of mental health disorders (Depression, GAD), hist. of overnight hospitalization, alcohol use, alcohol and drug abuse diagnoses, hist. or suicide attempt and ideation, hist. of medication, hist. of other treatment, drug use and abuse, alcohol use and abuse, history of drug-related, alcohol-related, and violent arrests. Also influenced by handgun purchasing data from the California Department of Justice: history of other felonies. | Recalculated at each time step. | DCJS |
| Probability of having a drug-related misdemeanor conviction | 0 – 1 | Calculated from the New York State Division of Criminal Justice Services based on race, sex, age, and borough. | Recalculated at any time step where agent has been sentenced with a drug-related arrest | -- |
| Probability of having a drug-related felony conviction | 0 – 1 | Calculated from the New York State Division of Criminal Justice Services based on race, sex, age, and borough. | Recalculated at any time step where agent has been sentenced with a drug-related arrest | -- |
| Probability of having an alcohol-related misdemeanor conviction | 0 – 1 | Calculated from the New York State Division of Criminal Justice Services based on race, sex, age, and borough. | Recalculated at any time step where agent has been sentenced with an alcohol-related arrest | -- |
| Probability of having an alcohol-related felony conviction | 0 – 1 | Calculated from the New York State Division of Criminal Justice Services based on race, sex, age, and borough. | Recalculated at any time step where agent has been sentenced with an alcohol-related arrest | -- |
| Probability of having a violent felony conviction | 0 – 1 | Calculated from the New York State Division of Criminal Justice Services based on race, sex, age, and borough. | Recalculated at any time step where agent has been sentenced with a violent arrest | -- |
| Probability of having another type of felony conviction | 0 – 1 | Calculated from the New York State Division of Criminal Justice Services based on race, sex, age, and borough. | Recalculated at any time step where agent has been sentenced with another arrest | -- |
| Probability of going to prison | 0 – 1 | Calculated from NYC Department of Corrections statistics based on age, race, and sex. Also influenced by Justice Atlas data based on community district. | Recalculated at any time step where agent has been convicted of any type of felony | -- |
| Sentence length in prison | 1, 2, 5, 15, or 35 years | Calculated from Survey of Inmates in State and Federal Correctional Facilities data and based on sex, age, race, education, drug use, history of violent victimization, number of times incarcerated before, kind of arrest, and having friends who are incarcerated. | Recalculated any time step an agent is sent to prison | -- |
| *Social network characteristics* | | | | |
| Number of non-drinkers | 0-9 | Calculated as the number of social network members who were non-, light/moderate, and heavy drinkers, respectively. | Recalculated at each time step. | Emerges from model |
| Number of  light/moderate drinkers | 0-9 |  |  |  |
| Number of heavy  drinkers | 0-9 |  |  |  |
| Number of victims | 0, 1, 2 or more | Calculated as the number of social network members who were victimized at last time step. | Recalculated at each time step. | Emerges from model |
| Number of perpetrators | 0, 1, 2 or more | Calculated as the number of social network members who perpetrated violence at last time step. | Recalculated at each time step. | Emerges from model |
| Number of firearm owners | 0, 1, 2 or more | Calculated as the number of social network members who owned firearms at last time step. | Recalculated at each time step. | Emerges from model |
| *Neighborhood characteristics* | | | | |
| Average household  income | < $44,000; $44,000-$57,999; ≥ $58,000 | Calculated as average income of neighborhood residents. | Recalculated at each time step. | Emerges from model |
| Average violent  victimization | 0-1 | Calculated as proportion of neighborhood residents who were victimized at last time step. | Recalculated at each time step. | Emerges from model |
| Average depression | 0-1 | Calculated as proportion of neighborhood residents who were depressed at last time step. | Recalculated at each time step. | Emerges from model |
| Average drug use | 0-1 | Calculated as proportion of neighborhood residents who used marijuana at last time step. | Recalculated at each time step. | Emerges from model |
| Average alcohol use | 0-1 for light drinkers, 0-1 for heavy drinkers | Calculated as proportion of neighborhood residents who were light or heavy drinkers at last time step. | Recalculated at each time step. | Emerges from model |
| Average suicide attempt | 0-1 | Calculated as proportion of neighborhood residents who attempted suicide at last time step. | Recalculated at each time step. | Emerges from model |
| Average suicide ideation | 0-1 | Calculated as proportion of neighborhood residents who thought about suicide at last time step. | Recalculated at each time step. | Emerges from model |
| Average firearm ownership | 0-1 | Calculated as proportion of neighborhood residents who owned a firearm at last time step. | Recalculated at each time step. | Emerges from model |
| Racial composition | 0-1 for each of Black and Hispanic | Calculated as proportion of neighborhood residents who were Black race and Hispanic ethnicity at last time step, respectively. | Recalculated at each time step. | Emerges from model |
| Young males | 0-1 | Calculated as proportion of neighborhood residents who were male and aged 18-24 years. | Recalculated at each time step. | Emerges from model |
| Residential stability | 0-1 | Calculated as proportion of neighborhood residents who have lived in the neighborhood for at least one year. | Recalculated at each time step. | Emerges from model |
| Foreign-born | 0-1 | Proportion of neighborhood residents who were foreign-born (i.e., not born in the United States). | -- | American Community Survey, 2008-2012 |
| Professional  occupations | 0-1 | Proportion of neighborhood residents in professional or managerial occupations. | -- | American Community Survey, 2008-2012 |
| Unemployment | 0-1 | Proportion of neighborhood residents who were unemployed. | -- | American Community Survey, 2008-2012 |
| Female-headed  households with  children | 0-1 | Percent of neighborhood residents residing in female-headed households with children under 18 years old. | -- | American Community Survey, 2008-2012 |

**Table A2**. Agent-based model initialization parameters and default values

| **Parameter** | **Value** |
| --- | --- |
| Number of agents | 800,000 |
| Number of neighborhoods | 59 |
| Neighborhood influence on agent behaviors ^a^ | 0.10 |
| Social network influence on agent behaviors ^b^ | 0.15 |
| Cell radius searched by potential perpetrator for potential victims of violence | 15 |
| Cell radius in which police officers can prevent violence | 2 |

^a^ Proportion of the probability of agent behaviors that is determined by the agent’s neighborhood characteristics

^b^ Proportion of the probability of agent behaviors that is determined by the agent’s social network characteristics

**References:**

[1] Grimm V, Berger U, Bastiansen F, et al. A standard protocol for describing individual-based and agent-based models. Ecol Modell 2006;198:115–26.

[2] Grimm V, Berger U, DeAngelis DL, et al. The ODD protocol: A review and first update. Ecol Modell 2010;221:2760–8.

[3] Müller B, Bohn F, Dreßler G, et al. Describing human decisions in agent-based models – ODD + D, an extension of the ODD protocol. Environ Model Softw 2013;48:37–48.

[4] New York City Department of City Planning. Community Portal. Available at: https://www1.nyc.gov/site/planning/community/community-portal.page. AccessedFebruary 13, 2018.

[5] U.S. Census Bureau 2010. Summary Files 1.

[6] Infoshare Online. American Community Survey, 2006-2010 5-yr average. Available at: http://www.infoshare.org. AccessedJanuary 24, 2019.

[7] Infoshare Online. American Community Survey, 2012-2016 5-yr average. Available at: http://www.infoshare.org. AccessedJanuary 31, 2019.

[8] Marsden P V. Core Discussion Networks of Americans. Am Sociol Rev 1987;52:122–31.

[9] Boardman JD, Finch BK, Ellison CG, et al. Neighborhood disadvantage, stress, and drug use among adults. J Health Soc Behav 2001;42:151–65.

[10] Sampson RJ, Groves WB. Community Structure and Crime: Testing Social-Disorganization Theory. Am J Sociol 1989;94:774–802.

[11] Selner-O’Hagan MB, Kindlon DJ, Buka SL, et al. Assessing exposure to violence in urban youth. J Child Psychol Psychiatry Allied Discip 1998;39:215–24.

[12] FE Z. The Youth Violence Epidemic: Myth or Reality. Wake Forest Law Rev 1998;33.

[13] Tracy M, Braga AA, Papachristos A V. The Transmission of Gun and Other Weapon-Involved Violence Within Social Networks. Epidemiol Rev 2016;38:70–86.

[14] New York City Department of Health and Mental Hygiene. Summary of Vital Statistics 2010: The City of New York. Available at: https://www1.nyc.gov/assets/doh/downloads/pdf/vs/2010sum.pdf. AccessedJanuary 29, 2019.

[15] Kessler RC, Merikangas KR. The National Comorbidity Survey Replication (NCS-R). Int J Methods Psychiatr Res 2004;13:60–8.

[16] McGinty EE, Choksy S, Wintemute GJ. The Relationship between Controlled Substances and Violence. Epidemiol Rev 2016;38:5–31.

[17] Swanson JW, Easter MM, Robertson AG, et al. Gun violence, mental illness, and laws that prohibit gun possession: Evidence from two florida counties. Health Aff 2016;35:1067–75.

[18] Bovasso G. Assessing the risk of threats with guns in the general population. J Threat Assess Manag 2014;1:27–39.

[19] Almeling R, Gadarian SK. Public opinion on policy issues in genetics and genomics. Genet Med 2014;16:491–4.

[20] Blanco C, Krueger RF, Hasin DS, et al. Mapping common psychiatric disorders: structure and predictive validity in the national epidemiologic survey on alcohol and related conditions. JAMA Psychiatry 2013;70:199–208.

[21] Wintemute GJ. Association between firearm ownership, firearm-related risk and risk reduction behaviours and alcohol-related risk behaviours. Inj Prev 2011;17:422–7.

[22] Carter PM, Walton MA, Newton MF, et al. Firearm Possession Among Adolescents Presenting to an Urban Emergency Department for Assault. Pediatrics 2013;132:213–21.

[23] Latalova K, Kamaradova D, Prasko J. Violent victimization of adult patients with severe mental illness: A systematic review. Neuropsychiatr Dis Treat 2014;10:1925–39.

[24] Olfson M, Wall M, Wang S, et al. Short-Term suicide risk after psychiatric hospital discharge. JAMA Psychiatry 2016;73:1119–26.

[25] New York States Office of Mental Health. GUIDELINES FOR COMPLETING THE 2013 PATIENT CHARACTERISTICS SURVEY. 2013.

[26] Swartz M. New York State Assisted Outpatient Treatment Evaluations: Review of Major Findings. Durham, NC: 2009.

[27] Goldmann E, Aiello A, Uddin M, et al. Pervasive exposure to violence and posttraumatic stress disorder in a predominantly African American Urban Community: The Detroit neighborhood health study. J Trauma Stress 2011;24:747–51.

[28] Sharkey P. Stuck in Place: Urban Neighborhoods and the End of Progress Toward Racial Equality. University of Chicago Press; 2013.

[29] Van Dorn R, Volavka J, Johnson N. Mental disorder and violence: Is there a relationship beyond substance use? Soc Psychiatry Psychiatr Epidemiol 2012;47:487–503.

[30] Branas CC, Elliott MR, Richmond TS, et al. Alcohol consumption, alcohol outlets, and the risk of being assaulted with a gun. Alcohol Clin Exp Res 2009;33:906–15.

[31] Goldstick JE, Lipton RI, Carter P, et al. The effect of neighborhood context on the relationship between substance misuse and weapons aggression in urban adolescents seeking ed care. Subst Use Misuse 2015;50:674–84.

[32] Wiebe DJ. Homicide and suicide risks associated with firearms in the home: A national case-control study. Ann Emerg Med 2003;41:771–82.

[33] Galea S, Ahern J, Tracy M, et al. Longitudinal determinants of posttraumatic stress in a population-based cohort study. Epidemiology 2008;19:47–54.

[34] Centers for Disease Control and Prevention, National Center for Health Statistics. Underlying cause of death 1999-2017.

[35] Messner SF, Galea S, Tardiff KJ, et al. Policing, drugs, and the homicide decline in New York City in the 1990s. Criminology 2007;45:385–414.

[36] Kaplan MS, McFarland BH, Huguet N, et al. Acute alcohol intoxication and suicide: A gender-stratified analysis of the National Violent Death Reporting System. Inj Prev 2012;19:38–43.

[37] Zlodre J, Fazel S. All-cause and external mortality in released prisoners: Systematic review and meta-analysis. Am J Public Health 2012;102.

[38] Spaulding AC, Seals RM, McCallum VA, et al. Prisoner survival inside and outside of the institution: Implications for health-care planning. Am J Epidemiol 2011;173:479–87.

[39] Bureau of Justice Statistics. Deaths In Custody Statistical Tables: State prison deaths, 2001-2007. n.d.

[40] Substance Abuse and Mental Health Services Administration. Results from the 2008 National Survey on Drug Use and Health: National Findings. Rockville, MD.: 2009.

[41] Olfson M, Wall M, Wang S, et al. Suicide following deliberate self-harm. Am J Psychiatry 2017;174:765–74.

[42] Kung HC, Pearson JL, Wei R. Substance use, firearm availability, depressive symptoms, and mental health service utilization among white and African American suicide decedents aged 15 to 64 years. Ann Epidemiol 2005;15:614–21.

[43] Miller M, Swanson SA, Azrael D. Are We Missing Something Pertinent? A Bias Analysis of Unmeasured Confounding in the Firearm-Suicide Literature. Epidemiol Rev 2016;38:62–9.

[44] Branas CC, Richmond TS, Culhane DP, et al. Investigating the link between gun possession and gun assault. Am J Public Health 2009;99:2034–40.

[45] New York State Department of Criminal Justice Services. Computerized Criminal History File: New York City Dispositions 2011-2017. 2019.

[46] Shaffer HJ, Nelson SE, LaPlante DA, et al. The Epidemiology of Psychiatric Disorders Among Repeat DUI Offenders Accepting a Treatment-Sentencing Option. J Consult Clin Psychol 2007;75:795–804.

[47] Wintemute GJ. Alcohol misuse, firearm violence perpetration, and public policy in the United States. Prev Med (Baltim) 2015;79:15–21.

[48] Osilla KC, Paddock SM, Leininger TJ, et al. A pilot study comparing in-person and web-based motivational interviewing among adults with a first-time DUI offense. Addict Sci Clin Pract 2015;10:18.

[49] Coker KL, Smith PH, Westphal A, et al. Crime and psychiatric disorders among youth in the US population: An analysis of the national comorbidity survey-adolescent supplement. J Am Acad Child Adolesc Psychiatry 2014;53:888–98.

[50] Cook PJ, Ludwig J, Travis J. Guns in America: National Survey on Private Ownership and Use of Firearms. Washington, DC: 1997.

[51] Substance Abuse and Mental Health Services Administration. Key substance use and mental health indicators in the United States: Results from the 2016 National Survey on Drug Use and Health. Rockville, MD: 2019.

[52] Department of Correction. Prison Population Demographics Q3 2016. Available at: http://www1.nyc.gov/site/doc/about/doc-statistics.page. AccessedOctober 7, 2016.

[53] Cardora E. Justice Atlas of Sentencing and Corrections. Available at: https://www.justiceatlas.org/. AccessedMay 24, 2018.

[54] Bureau of Justice Statistics. Survey of Inmates in State Correctional Facilities (SISCF), 2004. n.d.

[55] Cavanagh JTO, Carson AJ, Sharpe M, et al. Psychological autopsy studies of suicide: A systematic review. Psychol Med 2003;33:395–405.

[56] Kaplan MS, McFarland BH, Huguet N. Characteristics of adult male and female firearm suicide decedents: Findings from the National Violent Death Reporting System. Inj Prev 2009;15:322–7.

[57] Papachristos A V., Wildeman C, Roberto E. Tragic, but not random: The social contagion of nonfatal gunshot injuries. Soc Sci Med 2015;125:139–50.
