# Supplementary material for: Would restricting firearm purchases due to alcohol- and drug-related misdemeanor offenses reduce firearm homicide and suicide? An agent-based simulation
Source: Inj Epidemiol. 2022 Jun 9;9:17. doi: 10.1186/s40621-022-00381-x (PMC9185952; doi:10.1186/s40621-022-00381-x)
Supplement: Supplementary file 2 — Additional file 2. eTable 1 Sensitivity analysis reducing firearm ownership: simulation of gun-related deaths among the total agent population. eTable 2 Sensitivity analysis reducing firearm ownership: simulation of gun-related deaths among the high-risk groups. eTable 3 Sensitivity analysis decreasing social network influence: simulation of gun-related deaths among the total agent population. eTable 4 Sensitivity analysis decreasing social network influence: simulation of gun-related deaths among the high-risk groups. eTable 5 Sensitivity analysis increasing social network influence: simulation of gun-related deaths among the total agent population. eTable 6 Sensitivity analysis increasing social network influence: simulation of gun-related deaths among the high-risk groups [file 40621_2022_381_MOESM2_ESM.docx]

eTable 1. Sensitivity Analysis, reduce firearm ownership to 11%: Simulation of gun-related homicide and suicide in New York City after implementation of substance-related ownership disqualifications, among the total agent population.

| **Intervention** | **Gun Related Homicide** | | | | | | **Gun Related Suicide** | | | | | |
| --- | --- | --- | --- | --- | --- | --- | --- | --- | --- | --- | --- | --- |
|  | Rate/100,000 (95% CI) | | | % Decrease (95% CI) | | | Rate/100,000 (95% CI) | | | % Decrease (95% CI) | | |
|  | Mean | LL | UL | Mean | LL | UL | Mean | LL | UL | Mean | LL | UL |
| **Baseline** | **4.01** | **3.99** | **4.03** |  |  |  | **0.94** | **0.93** | **0.96** |  |  |  |
| **Alcohol misdemeanor conv. Last yr, 5 yr duration** | 3.98 | 3.95 | 4.00 | 0.79 | 0.22 | 1.35 | 0.91 | 0.90 | 0.92 | 3.30 | 2.01 | 4.60 |
| **Alcohol misdemeanor conv. Last yr, 10 yr duration** | 3.96 | 3.94 | 3.99 | 1.08 | 0.51 | 1.66 | 0.90 | 0.89 | 0.91 | 4.44 | 3.23 | 5.64 |
| **2+ Alcohol mis conv. Last 5 yrs, 10 yr duration** | 3.98 | 3.96 | 4.01 | 0.56 | -0.08 | 1.21 | 0.93 | 0.92 | 0.95 | 1.03 | -0.24 | 2.31 |
| **Any Alcohol last yr, 5 yr duration** | 3.96 | 3.93 | 3.99 | 1.17 | 0.53 | 1.82 | 0.91 | 0.90 | 0.92 | 3.38 | 2.02 | 4.73 |
| **Any Alcohol last yr, 10 yr duration** | 3.95 | 3.92 | 3.97 | 1.52 | 0.93 | 2.11 | 0.91 | 0.90 | 0.92 | 3.40 | 2.15 | 4.64 |
| **2+ Alcohol last 5 yrs, 10 yr duration** | 3.99 | 3.96 | 4.01 | 0.46 | -0.13 | 1.05 | 0.94 | 0.92 | 0.95 | 0.70 | -0.70 | 2.10 |
| **Drug misdemeanor conv. Last yr, 5 yr duration** | 3.93 | 3.90 | 3.96 | 1.98 | 1.27 | 2.68 | 0.90 | 0.88 | 0.91 | 4.74 | 3.24 | 6.25 |
| **Drug misdemeanor conv. Last yr, 10 yr duration** | 3.90 | 3.88 | 3.92 | 2.63 | 2.07 | 3.20 | 0.91 | 0.90 | 0.92 | 3.63 | 2.51 | 4.76 |
| **2+ Drug misdemeanor conv. Last 5 yrs, 10 yr duration** | 3.98 | 3.96 | 4.01 | 0.64 | 0.03 | 1.25 | 0.94 | 0.93 | 0.96 | 0.06 | -1.26 | 1.38 |
| **Any Drug arrest last yr, 5 yr duration** | 3.85 | 3.82 | 3.87 | 4.03 | 3.40 | 4.66 | 0.88 | 0.87 | 0.89 | 6.77 | 5.63 | 7.92 |
| **Any Drug arrest last yr, 10 yr duration** | 3.82 | 3.80 | 3.85 | 4.63 | 4.00 | 5.27 | 0.87 | 0.86 | 0.88 | 7.70 | 6.53 | 8.87 |
| **2+ Drug arrest last 5 yrs, 10 yr duration** | 3.95 | 3.93 | 3.98 | 1.39 | 0.77 | 2.01 | 0.92 | 0.91 | 0.93 | 2.57 | 1.43 | 3.70 |

eTable 2. Sensitivity Analysis, reduce firearm ownership to 11%: Simulation of gun-related homicide and suicide in New York City after implementation of substance-related ownership disqualifications, among high-risk groups.

| **Intervention** | **Gun Related Homicide** | | | | | | **Gun Related Suicide** | | | | | |
| --- | --- | --- | --- | --- | --- | --- | --- | --- | --- | --- | --- | --- |
|  | Rate/100,000 (95% CI) | | | % Decrease (95% CI) | | | Rate/100,000 (95% CI) | | | % Decrease (95% CI) | | |
|  | Mean | LL | UL | Mean | LL | UL | Mean | LL | UL | Mean | LL | UL |
| **Alcohol Misdemeanor** | | | | | | | | | | | | |
| **Base: Alcohol mis conviction ever** | **8.08** | **7.94** | **8.22** |  |  |  | **2.01** | **1.94** | **2.09** |  |  |  |
| **Alcohol mis conv. ever, 5yr duration** | 7.50 | 7.36 | 7.65 | 7.17 | 5.40 | 8.94 | 1.54 | 1.48 | 1.60 | 23.42 | 20.37 | 26.48 |
| **Alcohol mis conv. ever, 10yr duration** | 7.39 | 7.25 | 7.53 | 8.54 | 6.79 | 10.28 | 1.46 | 1.39 | 1.53 | 27.43 | 24.10 | 30.76 |
| **Base: 2+ Alcohol mis conv. in 5 years, ever** | **8.55** | **7.68** | **9.42** |  |  |  | **5.71** | **5.02** | **6.41** |  |  |  |
| **2+ Alcohol mis conv. in 5 years, ever** | 6.24 | 5.54 | 6.95 | 26.99 | 18.76 | 35.21 | 3.65 | 3.08 | 4.22 | 36.17 | 26.20 | 46.13 |
| **Alcohol Arrest** | | | | | | | | | | | | |
| **Base: Any Alcohol ever** | **6.40** | **6.21** | **6.59** |  |  |  | **2.91** | **2.78** | **3.04** |  |  |  |
| **Any Alcohol ever, 5yr duration** | 4.90 | 4.72 | 5.09 | 23.34 | 20.52 | 26.16 | 1.96 | 1.85 | 2.07 | 32.78 | 29.08 | 36.47 |
| **Any Alcohol ever, 10yr duration** | 4.81 | 4.66 | 4.96 | 24.84 | 22.53 | 27.15 | 1.81 | 1.71 | 1.90 | 37.91 | 34.62 | 41.21 |
| **Base: 2+ Alcohol in 5 years, ever** | **8.41** | **7.63** | **9.18** |  |  |  | **5.41** | **4.79** | **6.02** |  |  |  |
| **2+ Alcohol in 5 years, ever** | 6.35 | 5.71 | 6.99 | 24.48 | 16.82 | 32.15 | 2.83 | 2.47 | 3.20 | 47.58 | 40.83 | 54.33 |
| **Drug Misdemeanor** | | | | | | | | | | | | |
| **Base: Drug mis conv. ever** | **9.34** | **9.27** | **9.40** |  |  |  | **1.52** | **1.49** | **1.55** |  |  |  |
| **Drug mis conv. ever, 5yr duration** | 9.06 | 8.98 | 9.14 | 2.95 | 2.12 | 3.79 | 1.40 | 1.36 | 1.43 | 8.20 | 5.91 | 10.48 |
| **Drug mis conv. ever, 10yr duration** | 9.00 | 8.94 | 9.07 | 3.59 | 2.90 | 4.28 | 1.40 | 1.37 | 1.42 | 8.20 | 6.46 | 9.94 |
| **Base: 2+ Drug mis conv. in 5 years, ever** | **13.29** | **12.81** | **13.77** |  |  |  | **3.17** | **2.94** | **3.41** |  |  |  |
| **2+ Drug mis conv. in 5 years, ever** | 12.95 | 12.49 | 13.40 | 2.55 | -0.88 | 5.97 | 3.06 | 2.85 | 3.26 | 3.71 | -2.61 | 10.03 |
| **Drug Arrest** | | | | | | | | | | | | |
| **Base: Any Drug arrest ever** | **8.47** | **8.39** | **8.55** |  |  |  | **1.77** | **1.74** | **1.81** |  |  |  |
| **Any Drug arrest ever, 5yr duration** | 7.64 | 7.55 | 7.73 | 9.83 | 8.76 | 10.89 | 1.44 | 1.40 | 1.47 | 18.89 | 16.92 | 20.85 |
| **Any Drug arrest ever, 10yr duration** | 7.48 | 7.40 | 7.57 | 11.71 | 10.70 | 12.72 | 1.38 | 1.34 | 1.41 | 22.41 | 20.30 | 24.51 |
| **Base: 2+ Drug arrest in 5 years, ever** | **13.52** | **13.30** | **13.75** |  |  |  | **2.38** | **2.28** | **2.47** |  |  |  |
| **2+ Drug arrest in 5 years, ever** | 12.90 | 12.68 | 13.11 | 4.63 | 3.00 | 6.25 | 2.05 | 1.96 | 2.13 | 13.77 | 10.17 | 17.37 |

eTable 3. Sensitivity Analysis, adjust social network influence to 10%: Simulation of gun-related homicide and suicide in New York City after implementation of substance-related ownership disqualifications, among the total agent population.

| **Intervention** | **Gun Related Homicide** | | | | | | **Gun Related Suicide** | | | | | |
| --- | --- | --- | --- | --- | --- | --- | --- | --- | --- | --- | --- | --- |
|  | Rate/100,000 (95% CI) | | | % Decrease (95% CI) | | | Rate/100,000 (95% CI) | | | % Decrease (95% CI) | | |
|  | Mean | LL | UL | Mean | LL | UL | Mean | LL | UL | Mean | LL | UL |
| **Baseline** | **4.09** | **4.07** | **4.12** |  |  |  | **1.01** | **0.99** | **1.02** |  |  |  |
| **DUI misd conv. last yr, 5 yr duration** | 4.06 | 4.03 | 4.08 | 0.94 | 0.31 | 1.58 | 0.98 | 0.96 | 0.99 | 2.78 | 1.48 | 4.08 |
| **DUI misd conv. last yr, 10 yr duration** | 4.05 | 4.02 | 4.08 | 1.01 | 0.32 | 1.70 | 0.97 | 0.96 | 0.98 | 3.45 | 2.35 | 4.56 |
| **2+ DUI mis conv. last 5 yrs, 10 yr duration** | 4.08 | 4.05 | 4.10 | 0.47 | -0.14 | 1.07 | 1.01 | 1.00 | 1.02 | -0.44 | -1.69 | 0.81 |
| **Any DUI last yr, 5 yr duration** | 4.06 | 4.04 | 4.08 | 0.93 | 0.42 | 1.44 | 0.97 | 0.96 | 0.98 | 3.41 | 2.13 | 4.70 |
| **Any DUI last yr, 10 yr duration** | 4.05 | 4.02 | 4.08 | 1.08 | 0.42 | 1.74 | 0.97 | 0.96 | 0.98 | 3.68 | 2.45 | 4.92 |
| **2+ DUI last 5 yrs, 10 yr duration** | 4.06 | 4.04 | 4.09 | 0.80 | 0.20 | 1.41 | 1.00 | 0.99 | 1.01 | 0.75 | -0.41 | 1.90 |
| **Drug misd conv. last yr, 5 yr duration** | 4.00 | 3.98 | 4.03 | 2.20 | 1.63 | 2.76 | 0.96 | 0.95 | 0.97 | 4.28 | 3.03 | 5.54 |
| **Drug misd conv. last yr, 10 yr duration** | 3.99 | 3.96 | 4.01 | 2.66 | 2.02 | 3.29 | 0.95 | 0.94 | 0.96 | 5.17 | 4.03 | 6.31 |
| **2+ Drug misd conv. last 5 yrs, 10 yr duration** | 4.07 | 4.04 | 4.10 | 0.66 | -0.03 | 1.35 | 1.00 | 0.98 | 1.01 | 0.94 | -0.34 | 2.22 |
| **Any Drug arrest last yr, 5 yr duration** | 3.90 | 3.88 | 3.93 | 4.63 | 4.01 | 5.25 | 0.93 | 0.91 | 0.94 | 7.83 | 6.62 | 9.04 |
| **Any Drug arrest last yr, 10 yr duration** | 3.88 | 3.86 | 3.91 | 5.19 | 4.62 | 5.75 | 0.91 | 0.90 | 0.93 | 9.24 | 7.97 | 10.52 |
| **2+ Drug arrest last 5 yrs, 10 yr duration** | 4.05 | 4.02 | 4.07 | 1.20 | 0.69 | 1.71 | 0.99 | 0.98 | 1.00 | 1.66 | 0.34 | 2.99 |

eTable 4. Sensitivity Analysis, adjust social network influence to 10%: Simulation of gun-related homicide and suicide in New York City after implementation of substance-related ownership disqualifications, among high-risk groups.

| **Intervention** | **Gun Related Homicide** | | | | | | **Gun Related Suicide** | | | | | |
| --- | --- | --- | --- | --- | --- | --- | --- | --- | --- | --- | --- | --- |
|  | Rate/100,000 (95% CI) | | | % Decrease (95% CI) | | | Rate/100,000 (95% CI) | | | % Decrease (95% CI) | | |
|  | Mean | LL | UL | Mean | LL | UL | Mean | LL | UL | Mean | LL | UL |
| **Alcohol Misdemeanor** | | | | | | | | | | | | |
| **Base: Alcohol mis conviction ever** | **8.45** | **8.28** | **8.62** |  |  |  | **2.01** | **1.93** | **2.08** |  |  |  |
| **Alcohol mis conv. ever, 5yr duration** | 7.76 | 7.59 | 7.92 | 8.21 | 6.29 | 10.13 | 1.60 | 1.53 | 1.68 | 20.11 | 16.23 | 23.99 |
| **Alcohol mis conv. ever, 10yr duration** | 7.73 | 7.57 | 7.89 | 8.55 | 6.68 | 10.42 | 1.51 | 1.44 | 1.58 | 24.71 | 21.32 | 28.10 |
| **Base: 2+ Alcohol mis conv. in 5 years, ever** | **8.48** | **7.70** | **9.25** |  |  |  | **4.51** | **3.96** | **5.07** |  |  |  |
| **2+ Alcohol mis conv. in 5 years, ever** | 5.73 | 5.02 | 6.44 | 32.38 | 24.06 | 40.71 | 3.12 | 2.64 | 3.61 | 30.75 | 19.94 | 41.55 |
| **Alcohol Arrest** | | | | | | | | | | | | |
| **Base: Any Alcohol ever** | **6.00** | **5.81** | **6.19** |  |  |  | **2.83** | **2.72** | **2.95** |  |  |  |
| **Any Alcohol ever, 5yr duration** | 4.38 | 4.24 | 4.53 | 26.97 | 24.51 | 29.43 | 1.93 | 1.84 | 2.02 | 31.91 | 28.77 | 35.05 |
| **Any Alcohol ever, 10yr duration** | 4.29 | 4.14 | 4.44 | 28.47 | 25.95 | 30.99 | 1.80 | 1.70 | 1.91 | 36.29 | 32.60 | 39.98 |
| **Base: 2+ Alcohol in 5 years, ever** | **8.37** | **7.66** | **9.07** |  |  |  | **4.24** | **3.73** | **4.76** |  |  |  |
| **2+ Alcohol in 5 years, ever** | 5.26 | 4.58 | 5.94 | 37.14 | 29.00 | 45.28 | 2.88 | 2.47 | 3.30 | 32.04 | 22.28 | 41.81 |
| **Drug Misdemeanor** | | | | | | | | | | | | |
| **Base: Drug mis conv. ever** | **9.50** | **9.42** | **9.58** |  |  |  | **1.58** | **1.55** | **1.61** |  |  |  |
| **Drug mis conv. ever, 5yr duration** | 9.16 | 9.09 | 9.23 | 3.61 | 2.87 | 4.34 | 1.45 | 1.42 | 1.48 | 8.24 | 6.38 | 10.11 |
| **Drug mis conv. ever, 10yr duration** | 9.13 | 9.06 | 9.21 | 3.89 | 3.13 | 4.65 | 1.42 | 1.39 | 1.45 | 9.99 | 8.24 | 11.75 |
| **Base: 2+ Drug mis conv. in 5 years, ever** | **12.54** | **12.11** | **12.97** |  |  |  | **3.06** | **2.86** | **3.26** |  |  |  |
| **2+ Drug mis conv. in 5 years, ever** | 10.96 | 10.57 | 11.36 | 12.57 | 9.42 | 15.73 | 2.62 | 2.42 | 2.81 | 14.56 | 8.17 | 20.95 |
| **Drug Arrest** | | | | | | | | | | | | |
| **Base: Any Drug arrest ever** | **8.35** | **8.26** | **8.43** |  |  |  | **1.84** | **1.80** | **1.88** |  |  |  |
| **Any Drug arrest ever, 5yr duration** | 7.23 | 7.14 | 7.31 | 13.38 | 12.35 | 14.40 | 1.42 | 1.38 | 1.45 | 22.82 | 20.94 | 24.70 |
| **Any Drug arrest ever, 10yr duration** | 7.18 | 7.11 | 7.26 | 13.94 | 13.03 | 14.85 | 1.35 | 1.31 | 1.39 | 26.65 | 24.58 | 28.72 |
| **Base: 2+ Drug arrest in 5 years, ever** | **13.20** | **12.98** | **13.42** |  |  |  | **2.27** | **2.18** | **2.36** |  |  |  |
| **2+ Drug arrest in 5 years, ever** | 12.22 | 12.01 | 12.43 | 7.44 | 5.83 | 9.04 | 2.09 | 2.00 | 2.18 | 7.76 | 3.74 | 11.79 |

eTable 5. Sensitivity Analysis, adjust social network influence to 25%: Simulation of gun-related homicide and suicide in New York City after implementation of substance-related ownership disqualifications, among the total agent population.

| **Intervention** | **Gun Related Homicide** | | | | | | **Gun Related Suicide** | | | | | |
| --- | --- | --- | --- | --- | --- | --- | --- | --- | --- | --- | --- | --- |
|  | Rate/100,000 (95% CI) | | | % Decrease (95% CI) | | | Rate/100,000 (95% CI) | | | % Decrease (95% CI) | | |
|  | Mean | LL | UL | Mean | LL | UL | Mean | LL | UL | Mean | LL | UL |
| **Baseline** | **4.02** | **4.00** | **4.04** |  |  |  | **1.01** | **1.00** | **1.03** |  |  |  |
| **DUI misd conv. last yr, 5 yr duration** | 3.97 | 3.95 | 4.00 | 1.20 | 0.57 | 1.84 | 0.97 | 0.96 | 0.99 | 4.11 | 2.71 | 5.52 |
| **DUI misd conv. last yr, 10 yr duration** | 3.95 | 3.93 | 3.98 | 1.66 | 1.01 | 2.32 | 0.97 | 0.96 | 0.98 | 4.48 | 3.32 | 5.63 |
| **2+ DUI mis conv. last 5 yrs, 10 yr duration** | 4.00 | 3.98 | 4.03 | 0.50 | -0.13 | 1.12 | 1.01 | 0.99 | 1.02 | 0.56 | -0.77 | 1.89 |
| **Any DUI last yr, 5 yr duration** | 3.96 | 3.93 | 3.98 | 1.56 | 0.89 | 2.22 | 0.98 | 0.96 | 0.99 | 3.71 | 2.41 | 5.01 |
| **Any DUI last yr, 10 yr duration** | 3.95 | 3.93 | 3.98 | 1.66 | 1.07 | 2.25 | 0.97 | 0.96 | 0.98 | 4.11 | 2.92 | 5.30 |
| **2+ DUI last 5 yrs, 10 yr duration** | 4.00 | 3.97 | 4.02 | 0.62 | -0.01 | 1.25 | 1.00 | 0.99 | 1.02 | 0.96 | -0.38 | 2.31 |
| **Drug misd conv. last yr, 5 yr duration** | 3.94 | 3.92 | 3.97 | 1.99 | 1.37 | 2.62 | 0.97 | 0.96 | 0.98 | 4.36 | 3.18 | 5.54 |
| **Drug misd conv. last yr, 10 yr duration** | 3.93 | 3.91 | 3.95 | 2.21 | 1.64 | 2.79 | 0.96 | 0.95 | 0.97 | 5.35 | 4.12 | 6.58 |
| **2+ Drug misd conv. last 5 yrs, 10 yr duration** | 4.00 | 3.98 | 4.02 | 0.48 | -0.10 | 1.05 | 1.00 | 0.99 | 1.02 | 0.87 | -0.46 | 2.19 |
| **Any Drug arrest last yr, 5 yr duration** | 3.85 | 3.82 | 3.88 | 4.20 | 3.53 | 4.87 | 0.93 | 0.92 | 0.95 | 7.75 | 6.60 | 8.91 |
| **Any Drug arrest last yr, 10 yr duration** | 3.85 | 3.82 | 3.87 | 4.34 | 3.78 | 4.91 | 0.92 | 0.90 | 0.93 | 9.57 | 8.43 | 10.71 |
| **2+ Drug arrest last 5 yrs, 10 yr duration** | 3.98 | 3.96 | 4.00 | 0.99 | 0.40 | 1.58 | 0.98 | 0.97 | 1.00 | 2.86 | 1.50 | 4.23 |

eTable 6. Sensitivity Analysis, adjust social network influence to 25%: Simulation of gun-related homicide and suicide in New York City after implementation of substance-related ownership disqualifications, among high-risk groups.

| **Intervention** | **Gun Related Homicide** | | | | | | **Gun Related Suicide** | | | | | |
| --- | --- | --- | --- | --- | --- | --- | --- | --- | --- | --- | --- | --- |
|  | Rate/100,000 (95% CI) | | | % Decrease (95% CI) | | | Rate/100,000 (95% CI) | | | % Decrease (95% CI) | | |
|  | Mean | LL | UL | Mean | LL | UL | Mean | LL | UL | Mean | LL | UL |
| **Alcohol Misdemeanor** | | | | | | | | | | | | |
| **Base: Alcohol mis conviction ever** | **8.35** | **8.21** | **8.49** |  |  |  | **2.05** | **1.97** | **2.12** |  |  |  |
| **Alcohol mis conv. ever, 5yr duration** | 7.50 | 7.34 | 7.66 | 10.23 | 8.32 | 12.15 | 1.58 | 1.51 | 1.65 | 22.95 | 19.56 | 26.34 |
| **Alcohol mis conv. ever, 10yr duration** | 7.42 | 7.29 | 7.55 | 11.14 | 9.57 | 12.71 | 1.49 | 1.42 | 1.55 | 27.38 | 24.09 | 30.67 |
| **Base: 2+ Alcohol mis conv. in 5 years, ever** | **8.43** | **7.42** | **9.44** |  |  |  | **4.72** | **4.14** | **5.30** |  |  |  |
| **2+ Alcohol mis conv. in 5 years, ever** | 4.79 | 4.19 | 5.39 | 43.19 | 36.05 | 50.33 | 2.99 | 2.46 | 3.51 | 36.65 | 25.51 | 47.78 |
| **Alcohol Arrest** | | | | | | | | | | | | |
| **Base: Any Alcohol ever** | **5.96** | **5.78** | **6.14** |  |  |  | **2.87** | **2.74** | **3.00** |  |  |  |
| **Any Alcohol ever, 5yr duration** | 4.61 | 4.47 | 4.76 | 22.63 | 20.20 | 25.06 | 1.89 | 1.79 | 1.99 | 34.16 | 30.72 | 37.60 |
| **Any Alcohol ever, 10yr duration** | 4.26 | 4.12 | 4.41 | 28.46 | 26.01 | 30.90 | 1.81 | 1.72 | 1.90 | 36.98 | 33.75 | 40.21 |
| **Base: 2+ Alcohol in 5 years, ever** | **8.39** | **7.53** | **9.25** |  |  |  | **4.42** | **3.88** | **4.95** |  |  |  |
| **2+ Alcohol in 5 years, ever** | 5.17 | 4.53 | 5.81 | 38.37 | 30.76 | 45.98 | 2.95 | 2.54 | 3.36 | 33.25 | 23.96 | 42.53 |
| **Drug Misdemeanor** | | | | | | | | | | | | |
| **Base: Drug mis conv. ever** | **9.28** | **9.21** | **9.34** |  |  |  | **1.58** | **1.55** | **1.61** |  |  |  |
| **Drug mis conv. ever, 5yr duration** | 8.98 | 8.91 | 9.06 | 3.14 | 2.31 | 3.96 | 1.43 | 1.40 | 1.45 | 9.74 | 7.98 | 11.51 |
| **Drug mis conv. ever, 10yr duration** | 8.93 | 8.86 | 8.99 | 3.77 | 3.08 | 4.46 | 1.41 | 1.39 | 1.44 | 10.49 | 8.62 | 12.36 |
| **Base: 2+ Drug mis conv. in 5 years, ever** | **11.95** | **11.51** | **12.39** |  |  |  | **2.85** | **2.66** | **3.04** |  |  |  |
| **2+ Drug mis conv. in 5 years, ever** | 11.54 | 11.09 | 11.99 | 3.41 | -0.37 | 7.19 | 2.63 | 2.42 | 2.84 | 7.68 | 0.34 | 15.03 |
| **Drug Arrest** | | | | | | | | | | | | |
| **Base: Any Drug arrest ever** | **8.17** | **8.09** | **8.25** |  |  |  | **1.81** | **1.77** | **1.85** |  |  |  |
| **Any Drug arrest ever, 5yr duration** | 7.33 | 7.25 | 7.41 | 10.28 | 9.29 | 11.26 | 1.47 | 1.43 | 1.51 | 18.91 | 16.81 | 21.00 |
| **Any Drug arrest ever, 10yr duration** | 7.16 | 7.08 | 7.23 | 12.41 | 11.46 | 13.35 | 1.36 | 1.32 | 1.39 | 25.08 | 23.03 | 27.13 |
| **Base: 2+ Drug arrest in 5 years, ever** | **12.93** | **12.70** | **13.15** |  |  |  | **2.27** | **2.19** | **2.36** |  |  |  |
| **2+ Drug arrest in 5 years, ever** | 12.32 | 12.12 | 12.52 | 4.68 | 3.13 | 6.22 | 1.96 | 1.87 | 2.04 | 13.87 | 10.10 | 17.64 |
